# *N*-Skatyltryptamines—Dual 5-HT_6_R/D_2_R Ligands with Antipsychotic and Procognitive Potential

**DOI:** 10.3390/molecules26154605

**Published:** 2021-07-29

**Authors:** Agata Hogendorf, Adam S. Hogendorf, Rafał Kurczab, Grzegorz Satała, Bernadeta Szewczyk, Paulina Cieślik, Gniewomir Latacz, Jadwiga Handzlik, Tomasz Lenda, Katarzyna Kaczorowska, Jakub Staroń, Ryszard Bugno, Beata Duszyńska, Andrzej J. Bojarski

**Affiliations:** 1 Department of Medicinal Chemistry, Maj Institute of Pharmacology, Polish Academy of Sciences, 12 Smętna Street, 31-343 Kraków, Poland; agatah@if-pan.krakow.pl (A.H.); ahogen@if-pan.krakow.pl (A.S.H.); kurczab@if-pan.krakow.pl (R.K.); satala@if-pan.krakow.pl (G.S.); k.kaczor@if-pan.krakow.pl (K.K.); staron@if-pan.krakow.pl (J.S.); bugno@if-pan.krakow.pl (R.B.); duszyn@if-pan.krakow.pl (B.D.); 2 Department of Neurobiology, Maj Institute of Pharmacology, Polish Academy of Sciences, 12 Smętna Street, 31-343 Kraków, Poland; szewczyk@if-pan.krakow.pl (B.S.); cieslik@if-pan.krakow.pl (P.C.); 3 Department of Technology and Biotechnology of Drugs, Jagiellonian University Medical College, Medyczna 9, 30-688 Kraków, Poland; glatacz@cm-uj.krakow.pl (G.L.); j.handzlik@uj.edu.pl (J.H.); 4 Department of Neuropsychopharmacology, Maj Institute of Pharmacology, Polish Academy of Sciences, 12 Smętna Street, 31-343 Kraków, Poland; lenda@if-pan.krakow.pl

**Keywords:** *N*-skatyltryptamine, tryptamine, D_2_/5-HT_6_R receptor agonist/antagonist, antipsychotic, precognitive, halogen bond, serotonin dual ligands

## Abstract

A series of *N*-skatyltryptamines was synthesized and their affinities for serotonin and dopamine receptors were determined. Compounds exhibited activity toward 5-HT_1A_, 5-HT_2A_, 5-HT_6_, and D_2_ receptors. Substitution patterns resulting in affinity/activity switches were identified and studied using homology modeling. Chosen hits were screened to determine their metabolism, permeability, hepatotoxicity, and CYP inhibition. Several D_2_ receptor antagonists with additional 5-HT_6_R antagonist and agonist properties were identified. The former combination resembled known antipsychotic agents, while the latter was particularly interesting due to the fact that it has not been studied before. Selective 5-HT_6_R antagonists have been shown previously to produce procognitive and promnesic effects in several rodent models. Administration of 5-HT_6_R agonists was more ambiguous—in naive animals, it did not alter memory or produce slight amnesic effects, while in rodent models of memory impairment, they ameliorated the condition just like antagonists. Using the identified hit compounds **15** and **18**, we tried to sort out the difference between ligands exhibiting the D_2_R antagonist function combined with 5-HT_6_R agonism, and mixed D_2_/5-HT_6_R antagonists in murine models of psychosis.

## 1. Introduction

The 5-HT_6_ receptor has gained the attention of the pharma industry as a putative target for novel cognitive enhancers in dementia [1,2,3] as well as an important co-target in the design of psychotropic drugs. The high affinity of several typical and atypical antipsychotics and tricyclic antidepressants for 5-HT_6_, taken together with its sole CNS distribution, notably in the hippocampus, nucleus accumbens, prefrontal cortex, and striatum, has prompted intensive research, which partially revealed the physiological function of the receptor, in particular, its association with GABAergic and cholinergic transmission [4,5].

5-HT_6_R blockade has been proposed as a treatment of Alzheimer’s disease (AD) symptoms, however, the strategy ultimately failed, with no drugs on the market so far despite intensive clinical trial campaigns led by several companies [6,7,8,9]. Given the lack of efficacy of selective 5-HT_6_R antagonists in AD, several polypharmacological approaches have been proposed instead [10,11,12,13,14,15].

Although 5-HT_6_R blockade on its own as a procognitive treatment in dementia failed, it is still a matter of debate whether the 5-HT_6_R component contributes to a beneficial profile of certain psychotropic drugs [16]. Cognitive decline has been an unresolved symptom of schizophrenia, which prohibits the patients from a return to work and social functioning. Co-administration of a 5-HT_6_ antagonist AVN-211 in patients with schizophrenia stabilized on antipsychotic treatment has resulted in an improvement in Positive and Negative Syndrome Scale score over the placebo group [17]. Interestingly, while only antagonists of 5-HT_6_R display memory-enhancing properties per se, both agonists and antagonists reverse the cognitive deficits in rodent models of schizophrenia [18,19,20].

NMDA antagonist administration is a well-established screening model of cognitive deficits resembling the symptoms of schizophrenia [21,22]. Typically, the impairment is induced by phencyclidine (PCP) or dizocilpine (MK-801) [23]. The clinical candidate LU AE58054, a 5-HT_6_R antagonist, has been shown to reverse the cognitive impairment induced by subchronic phencyclidine in NORT in rats [24]. Administration of MK-801 resulted in an increase in locomotion, increased pre-pulse inhibition, stereotypy, deficits in spatial memory, and impaired novel object recognition [25]. The effects of the administration of a selective 5-HT_6_R antagonist and agonist on MK-801 induced memory impairment in the NOR task have recently been published [20]. It has been shown that, counterintuitively, both ameliorated the MK-801 effects in NORT, which could have been related to the restoration of physiological BDNF levels. Tryptamines represent an important group of compounds exhibiting various biological activities, especially renowned as serotonin receptor ligands. A SAR study of benzyl-5-methoxytryptamines, a chemotype that was investigated due to the structural resemblance to the ultrapotent 5-HT ligands: *N*-benzyl-2,5-dimethoxyphenylethylamines, was published in 2015 by Nichols et al. [26]. Compounds were evaluated as 5-HT_2A_R ligands, but also screened against several other 5-HT receptors, revealing their promiscuous nature including affinity for 5-HT_6_R. Surprisingly, so far, little effort has been put to explore the chemical space of the *N*-arylmethyl-arylethylamines beyond the works that focused on benzyl derivatives of tryptamines and phenylethylamines as 5-HT_2A_R agonists. In this work, we present a part of our search for non-sulfonyl 5-HT_6_ receptor ligands within the aforementioned scaffold. Several mixed D_2_/5-HT_6_R ligands exhibiting different functional profiles have been discovered, and their ADMET properties explored.

While studying the literature, we did not come across any reports of mixed D_2_ antagonist–5-HT_6_ agonists in animal models of schizophrenia-related cognitive decline. A compound with such a functional profile–**18** was thus compared with a mixed D_2_/5-HT_6_R antagonist in screening models.

## 2. Results

### 2.1. Chemistry

**Scheme 1:**
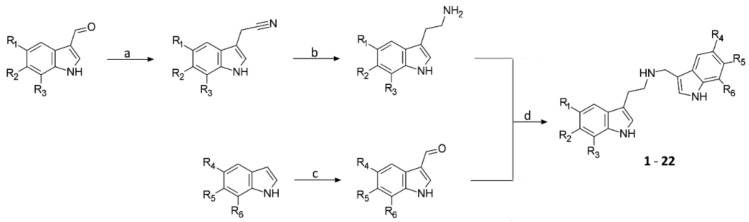
**a:** 1. NaBH_4_, HCONH_2_, MeOH 2. NaCN; **b**: AlCl_3_, LiAlH_4_, Et_2_O; **c**: POCl_3_, DMF; **d**: NaBH_4_, MeOH.

*N*-skatyltryptamines were synthesized using the procedure shown in Scheme 1. Several monosubstituted aldehydes were commercially available, and the others were synthesized via Vilsmeier–Haack formylation starting from appropriate monosubstituted indoles. The aldehydes were converted into the nitriles in a two-step, one-pot process involving reduction with NaBH_4_ and substitution with NaCN, supposedly going through 3-methylidene-3*H*-indol-1-ium species [27]. It was observed that LiAlH_4_ reduction of halogenated indole-3-carbonitriles resulted in partial dehalogenation of indole. Due to slight differences in the polarity of tryptamine and halotryptamines resulting in poor chromatography separation, a milder method was used instead. Indole-3-carbonitriles were converted into the corresponding primary amines using a mixture of AlCl_3_ and LiAlH_4_. The reductive amination with NaBH_4_ after imine formation allowed us to obtain the final products of *N*-skatyltryptamines in good yields. The spectral data and chromatograms can be found in Supplementary Materials.

### 2.2. Structure–Affinity Relationship

Compound **1**, [2-(1*H*-indol-3-yl)ethyl][(1*H*-indol-3-yl)methyl]amine, exhibited high affinity for 5-HT_2A_ and medium for the 5-HT_6_ receptor (*K*_i 5-HT2A_ = 20 nM and *K*_i 5-HT6_ = 172 nM, Table 1–preferred substitution patterns shown in color). Modification of position 5 of the tryptamine motif resulted in a decreased 5-HT_2A_R affinity (entries **2**–**4**), while 5-HT_6_ affinity was slightly increased. 5-Methoxytryptamine derivative **3** was also bound 5-HT_1A_R with considerable potency (*K*_i_ = 156 nM). Compound **5** with the chlorine atom in R2 showed a moderate affinity for 5-HT_6_R and D_2_R (*K*_i_ = 187 nM and 155 nM, respectively). A halogen atom (F, Cl or Br) introduced in position R3 resulted in compounds selective toward the 5-HT_6_ receptor (**6**: *K*_i_ = 168 nM, **7**: *K*_i_ = 133 nM, **8**: *K*_i_ = 161 nM). Position R4 substituted with a hydroxyl group or a halogen atom (F, Cl, I) gave compounds with low affinity for 5-HT_6_R (entries **9**–**12**). Compound **13** with a fluorine atom in R5 showed low affinity for 5-HT_6_R (*K*_i_ = 225 nM) and D_2_R (*K*_i_ = 299 nM). Substitution of R5 with a chlorine or a bromine atom increased the affinity for 5-HT_6_R (**14**: *K*_i_ = 90 nM, **15**: *K*_i_ = 102 nM); in addition, compound **14** was also bound to D_2_R (*K*_i_ = 124 nM). Introducing the halogen atom in position R6 resulted in compounds that exhibited a high affinity only for the D_2_ receptor (**16**: *K*_i_ = 91 nM, **17**: *K*_i_ = 51 nM, **18**: *K*_i_ = 55 nM). Benzyloxy substitution in R6 (**19**) gave compounds with weak affinity for 5-HT_6_R (*K*_i_ = 303 nM). Disubstituted compounds were synthesized combining a 5-methoxytryptamine fragment with broad serotonin receptor affinity with an aldehyde component bearing a halogen atom that was thought to trigger D_2_R antagonism, while not interfering with 5-HT_6_R binding. The resulting compounds were thus substituted at both indole fragments with a methoxy at R1 and a chlorine at either R5 or R6 and exhibited high binding affinity for 5-HT_6_R (*K*_i_ = 98 nM and *K*_i_ = 53 nM, respectively) as for 5-HT_1A_R (*K*_i_ = 79 nM and *K*_i_ = 187 nM, respectively), but did not bind to D_2_R.

### 2.3. Functional Assays toward 5-HT_6_R and D_2_R

For the compounds with the highest binding affinity for 5-HT_6_R and D_2_R, intrinsic function toward 5-HT_6_R in agonistic and antagonistic mode and for D_2_R in antagonistic mode were evaluated (Table 2). Substitution in position R6 acted as an agonism switch for 5-HT_6_R, with compound **18** exhibiting EC_50 5-HT6_ = 77 nM. Switching from bromine to chlorine, and substitution with a methoxy group in position 5 of the tryptamine motif in compound **22** resulted in enhanced potency (EC_50 5-HT6_ = 26 nM), while the substitution pattern was R1 = methoxy, and R6 = chlorine comparable potency (**21**, EC_50 5-HT6_ = 22 nM).

For the D_2_ receptor, only the antagonistic function was observed for all the tested entries. The most efficacious were *N*-skatyltryptamines with halogens in positions R2 (**5**, EC_50_ = 104 nM) and R5 (**13** and **15**, EC_50_ = 91 nM and EC_50_ = 109 nM), respectively.

### 2.4. Molecular Modeling

**Figure 1:**
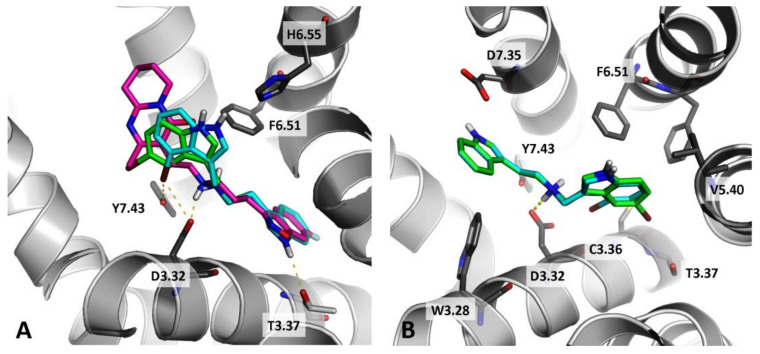
Illustration of the binding modes of selected compounds in the binding site of D_2_ and 5-HT_6_Rs. Comparison of binding modes of compound **15** (green), **18** (cyan) vs. risperidone (magenta) in D_2_R (**A**), and **15** vs. **18** in 5-HT_6_R (**B**).

Functional assays showed that small structural changes caused significant differences in intrisic function, thus the binding mode of compounds **15** and **18** was investigated to find the plausible molecular recognition site. Structures of compounds were docked to 5-HT_6_R homology models built on the β2 adrenergic receptor crystal structure as a template and to the D_2_ receptor (PDB ID: 6CM4). The QPLD was used to obtain ligand–receptor complexes, since this algorithm describes the anisotropy of the electron density of halogen atoms. In the acquired complexes, the bromine substituent played different roles in binding to the D_2_ and 5-HT_6_ receptors. Analysis of the binding modes showed that in the D_2_ binding site (Figure 1A), the bromine can be involved in the weak halogen bonds with D3.32 and Y7.43 side chains only for the 6-Br derivative. Both derivatives showed a similar binding mode to the pose of the risperidone from the crystal structure of the D_2_R complex (Figure 1A). However, the bromine in the 5-HT_6_R (Figure 1B) did not show any specific interactions, and it seems that in both derivatives, bromine plays a steric function (fitting to the shape of the binding site). These observations are in line with the in vitro data.

The structure–5-HT_6_R function relationship could not be analyzed based on the acquired complexes. The agonist–antagonist switch observed for the **15** and **18** pair could have been caused by the stabilization of the “flipped” ligand pose (i.e., with the skatyl fragment pointing down to the intracellular part of the receptor), presumably resulting in receptor antagonism. This, however, is a hypothesis that would need extensive, additional studies to confirm.

### 2.5. In Vitro ADMETox Studies

#### 2.5.1. Permeability Assay

The most common screening method to define compound permeability is the parallel artificial membrane permeability assay (PAMPA). This test allows one to determine the compounds’ passive penetration through the bilayer artificial membranes that mimic the barrier between the intestine wall and blood. Caffeine was used as a reference compound with high *Pe* value (*Pe =* 15.1 × 10^−6^ cm/s) and norfloxacin as a compound with very poor *Pe* value (*Pe* = 0.56 × 10^−6^ cm/s). All tested chlorine derivatives (**5**, **14**, and **17**) exhibited medium permeability coefficients (*Pe*) compared to caffeine (Table 3). However, compounds with the bromine substituent, **15** and **18**, showed rather low *Pe* value but still higher than norfloxacin (*Pe =* 2.42 × 10^−6^ cm/s and *Pe =* 3.90 × 10^−6^ cm/s for **15** and **18**, respectively).

#### 2.5.2. Metabolic Stability

To determine the most probable metabolite structures, compounds **5**, **14**, **15**, **17**, and **18** were incubated with MLMs (mouse liver microsomes) for prolonged time and mass spectra of the obtained mixtures were recorded. In the next step, MetaSite 6.01 software was applied to visualize the obtained in vitro data. In silico predictions indicated that the most probable biotransformation for *N*-skatyltryptamines is hydroxylation, which was in agreement with the in vitro experiment. The molecular masses and predicted products of metabolism are shown in Table 4. Almost all compounds were converted into two hydroxylated metabolites, except of compound **5**, which showed only one hydroxylated metabolite.

**Figure 2:**
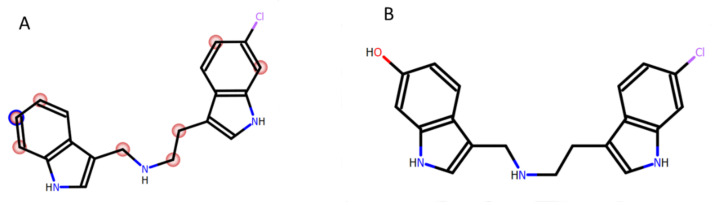
(**A**) Prediction of the sites of metabolism by MetaSite 6.01. Blue circle marked on the functional group structures indicates the highest biotransformation probability. The fading red color shows decreased probability of metabolism. (**B**) The most probable structure of **5** main metabolite.

**Figure 3:**
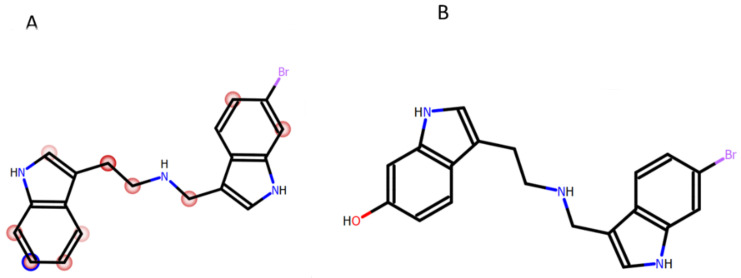
(**A**) Prediction of the sites of metabolism by MetaSite 6.01. Blue circle marked on the functional group structures indicates the highest biotransformation probability. The fading red color shows decreased metabolism probability. (**B**) The most probable structure of **15** main metabolite.

The hydroxylation of **5** was predicted to occur in position R5 (the aldehyde component derived indole fragment). For compounds with the unsubstituted tryptamine part of the molecule, the addition of hydroxyl group is indicated in the R2 position (Figure 2 and Figure 3). The remaining structures of predicted metabolites can be found in Supplementary Materials.

#### 2.5.3. CYP450 Inhibition

**Figure 4:**
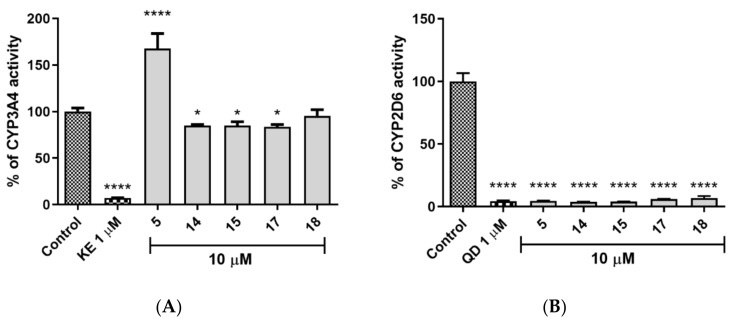
(**A**) Effect of reference inhibitor ketoconazole and **5**, **14**, **15**, **17**, **18** on CYP3A4 activity. Statistical significance (**** *p* < 0.0001, * *p* < 0.05) was analyzed by Graph Pad Prism 8.0.1 software using one-way ANOVA and Bonferroni’s multiple comparison post-test. The compounds were examined in triplicate. (**B**) Effect of reference inhibitor quinidine and **5**, **14**, **15**, **17**, and **18** on CYP2D6 activity. Statistical significance (**** *p* < 0.0001) was analyzed by Graph Pad Prism 8.0.1 software using one-way ANOVA and Bonferroni’s multiple comparison post-test. The compounds were examined in triplicate.

CYP2D6 inhibition is quite common for CNS drugs including antipsychotics such as sertindole [28]. The influence of compounds **5**, **14**, **15**, **17**, and **18** on CYP450 isoforms CYP3A4 and CYP2D6 were evaluated (Figure 4). In comparison to ketoconazole, which was used as the reference inhibitor of CYP3A4, compounds **14**, **15**, **17**, and **18** only slightly inhibited the activity of this isoform. In contrast, **5** acted as an inducer of CYP3A4. Regarding CYP2D6, all the tested compounds completely inhibited the activity of this isoform, which may result in potential drug–drug interactions.

#### 2.5.4. Hepatotoxicity

**Figure 5:**
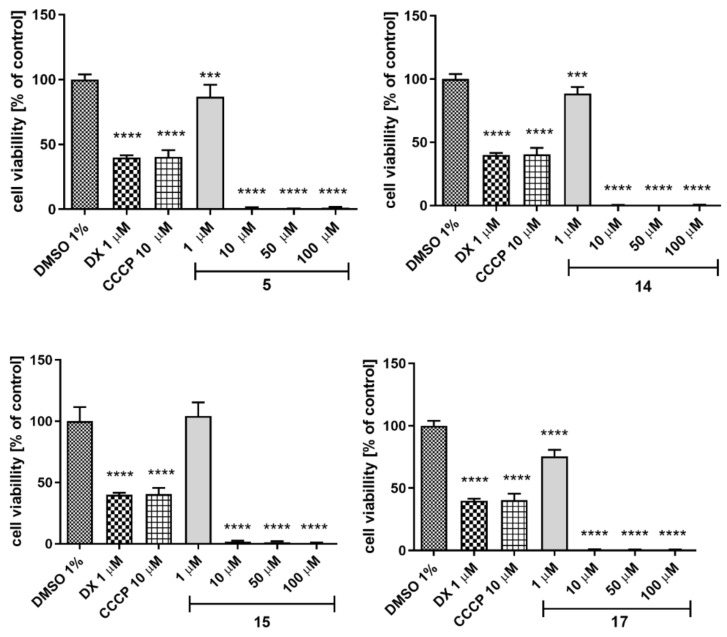

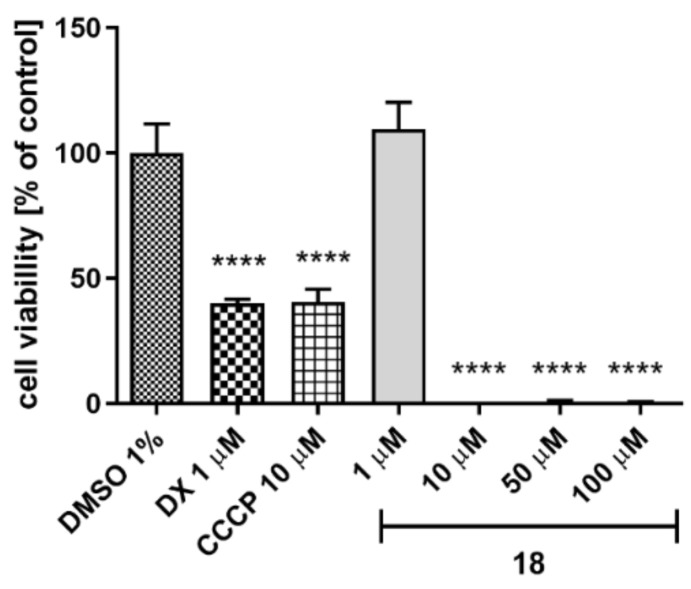
The effect of tested compounds (**5**, **14**, **15**, **17**, and **18**) on the viability of the HepG2 cell line. DMSO 1% in cell growth media (vehicle) was used as the control. Reference cytostatic drug doxorubicin (DX, 1 µM) and mitochondrial toxin CCCP were used as positive controls. Statistical significance (**** *p* < 0.0001, *** *p* < 0.001) was analyzed by Graph Pad Prism 8.0.1 software using one-way ANOVA and Bonferroni’s multiple comparison post-test. The compounds were examined in quadruplicate.

To investigate the hepatotoxicity of the new 5-HT_6_/D_2_ ligands, a cell-based assay using the HepG2 line was conducted. Compounds **5**, **14**, and **17** at 1 μM concentration showed a slight antiproliferative effect, where the cell viabilities were decreased to up to ~80% of the control (Figure 5). For bromo-derivatives **15** and **18** at 1 μM concentration, a proliferative effect was observed, but it was not statistically significant. All the higher concentrations of all compounds (10 μM, 50 μM, 100 μM) caused total cell death, clearly pointing to hepatotoxicity.

### 2.6. In Vivo Behavioral Tests

#### 2.6.1. MK-801-Induced Hyperactivity in Mice

**Figure 6:**
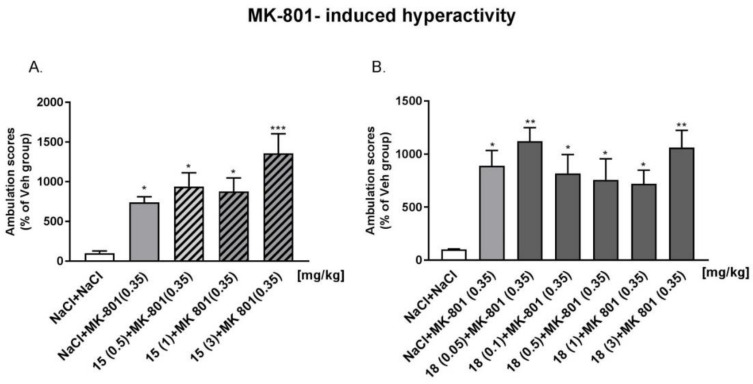
(**A**) Effect of compound **15** (0.5, 1, 3 mg/kg) and (**B**) **18** (0.05, 0.1, 0.5, 1, 3 mg/kg on MK-801–induced hyperactivity in Albino Swiss mice. The test compounds were given 30 min before MK-801 administration, which was given 30 min before the test. Locomotor activity was monitored over a 60 min session immediately following an injection of MK-801. The data are presented as mean ± SEM, *n* = 5–8 mice per group. Data were analyzed with one-way ANOVA and Newman–Keuls post-hoc. **15**: F(4, 29) = 5.293, *p* = 0.0025; **18**: F(6,43) = 3.653, *p* = 0.005; * *p* < 0.05; ** *p* < 0.01; *** *p* < 0.001 vs. NaCl + NaCl (Veh group).

Agitation, which is characteristic for schizophrenia-like behavior, can be modeled by the administration of NMDA antagonist MK-801. The potential antipsychotic activity of compounds **15** and **18** was thus evaluated in a MK-801-induced hyperactivity model in mice. The administration of MK-801 (0.35 mg/kg) significantly increased the activity of the mice compared to the control group (*p* < 0.05) in all doses. None of the tested compounds reversed MK-801-elevated activity (Figure 6).

#### 2.6.2. Novel Object Recognition (NOR) Test

**Figure 7:**
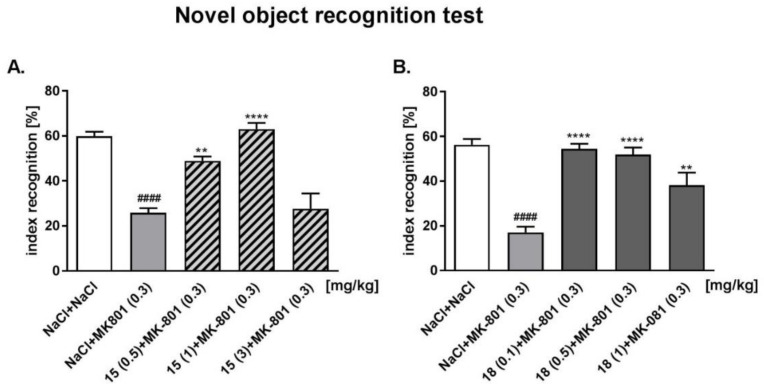
Novel object recognition test in mice. Effectivity of **15** (**A**) and **18** (**B**). Bars represent the means ± SEM, *n* = 5–10. Data were analyzed with one-way ANOVA and Newman–Keuls post-hoc. **15**: F(4,33) = 18.88, *p* < 0.0001; **18**: F(4,35) = 12.45, *p* < 0.0001; #### *p* < 0.0001 vs. Con; ** *p* < 0.01, **** *p* < 0.0001.

The effect of acute treatment with compounds **15** and **18** on the cognitive function in the novel object recognition test in mice was checked (Figure 7). Compound **15** reversed memory impairment induced by MK-801 (0.3 mg/kg) at doses of 0.5 and 1 mg/kg (*p* < 0.01, *p* < 0.0001), but not at 3 mg/kg. Compound **18** reversed memory impairment induced by MK-801 (0.3 mg/kg) at all tested doses (0.1; 0.5; 1 mg/kg); *p* < 0.0001, *p* < 0.0001, *p* < 0.01.

#### 2.6.3. Effect of Compound **15** and **18** on Spontaneous Activity of Mice

Compound **15** administered at the doses of 0.5, 1, and 3 mg/kg did not affect the locomotor activity of mice (Table 5, *p* > 0.05). Similarly, compound **18** administered at the doses of 0.05, 0.1, 0.5, 1, and 3 mg/kg did not influence the spontaneous locomotor activity of mice (*p* < 0.05).

Mice were placed separately into activity cages for an acclimatization period of 30 min, then they were injected i.p. with compound **15** (0.5, 1, and 3 mg/kg) or compound **18** (0.05, 0.1, 0.5, 1, and 3 mg/kg, respectively. After a further 30 min, they were injected with saline (10 mL/kg). From this point on, the ambulation scores were measured for 60 min. The data are presented as mean ± SEM, *n* = 5 mice per group. Data were analyzed with one-way ANOVA and Dunnett’s post-hoc. **15**: F(3,16) = 1.225, *p* = 0.333; **18**: F(5,24) = 0.791, *p* = 0.567.

## 3. Discussion

There have been reports of potent 5-HT receptor ligands belonging to the class of *N*-arylmethyl arylethylamines dating back to 1994 [29]. The exploration of this chemical was fueled by the discovery of ultrapotent 5-HT_2A_R agonists with hallucinogenic activity: *N*-benzyl[2-(2,5-dimethoxyphenyl) ethyl]amines. Despite relatively very high selectivity, the so called NBOMe compounds also bound to 5-HT_2B_, 5-HT_2C_, 5HT_6_, and opioid and histamine receptors [30]. The next important entry was the work of Nichols et al. on *N*-benzyl-5-methoxytryptamines, which exhibited high affinity for 5-HT_2A_R as well as for 5-HT_2B_ and a selectivity over 5-HT_1A_, 5-HT_1B_, 5-HT_1D_, 5-HT_1E_, 5-HT_3_, 5-HT_5A_, and 5-HT_7_ receptors. Three entries: **5b**, **5i**, and **5l** showed remarkable affinity for 5-HT_6_R (25, 27, and 10 nM, respectively).

**Figure 8:**
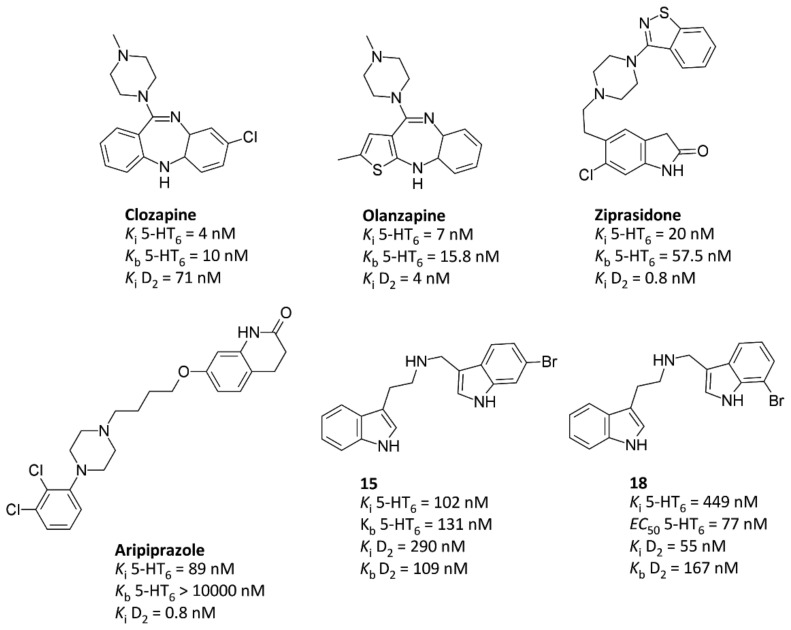
Atypical antipsychotics and the structures of compounds **15** and **18** from this work. Binding affinities of antipsychotic drugs and their antagonist function were taken from the work of Dupuis et al. (2008) [31].

In the presented work, a series of *N*-skatyltryptamines were synthesized and their affinities for 5-HT_1A_, 5-HT_2A_, 5-HT_6_, 5-HT_7_, and D_2_ receptors were determined. The study commenced with the synthesis of **1**, which turned out to be a 5-HT_2A_R partial agonist. Halogen substitution was applied to the parent compound **1** in order to search for positions enabling contacts with halogen bond acceptors within the receptors. Thus, selective D_2_R antagonists were discovered, along with mixed 5-HT_1A_R, 5-HT_6_R, and D_2_R antagonists and dual D_2_R, 5-HT_6_R ligands. 5-HT_2A_R affinity was observed only in the derivatives of 5-methoxytryptamine. The substitution with a halogen atom in position 6 or 7 (R5 and R6, respectively) of the indole within the skatyl fragment dramatically enhanced D_2_R affinity, while switching between the agonistic (with halogen at R6) and antagonistic (with halogen at R5) function at 5-HT_6_R. Disubstituted derivatives **21** and **22** acted as potent and selective 5-HT_6_R agonists. The ADMET study showed that despite expectations, free tryptamine fragments were not detected after prolonged incubation with mouse liver microsomes, with hydroxylation being the most prevalent metabolic pathway. The PAMPA model revealed a rather high passive permeability of the studied compounds. Although useful as molecular probes, *N*-skatyltryptamines turned out to be hepatotoxic, thus not suitable to further development as pharmaceutical drugs. The tested compounds were also strong inhibitors of CYP2D6 isoforms, indicating possible drug–drug interactions. The pharmacological profile of compounds **15** and **18** resembled that of atypical antipsychotics (Figure 8).

This pair of isomers differed by 5-HT_6_R function with **15** being an antagonist and **18** an agonist. It was thus a good opportunity to settle how a combination of D_2_R antagonist and 5-HT_6_R agonist or antagonist differed in behavioral models of psychosis and cognitive impairment. Contrary to antipsychotic drugs clozapine or haloperidol [32], **15** and **18** did not reverse the locomotor activity elevated by the administration of MK-801. The exacerbation of hyperactivity at 3 mg/kg of **15** was not statistically significant. In the novel object recognition test, compound **15** significantly reversed memory impairment after MK-801 administration at 0.5 and 1 mg/kg, while **18** at all doses. Both compounds did not affect spontaneous activity of mice (i.e., did not induce sedation). It is likely that at the tested doses, the 5-HT_6_R mediated effect was more apparent than the D_2_R blockade. However, at the 0.3–3 mg/kg range, in the behavioral models used, we could not spot the difference between the 5-HT_6_R agonist and antagonist, with both producing a procognitive effect.

The observed lack of difference in the action of **15** and **18** in the MK-801 impaired NORT may look bizarre. However, there have been reports of paradoxical actions of glutamatergic drugs such as different outcomes in healthy individuals and patients [33,34]. The observed similar effects of compounds **15** and **18** in NORT could possibly be related to the restored BDNF levels described by Rychtyk et al. [20]. The authors showed that both WAY-181187, which is a selective 5-HT_6_R agonist and SB-742457, a selective antagonist, alleviated the MK-801-induced inhibition of hippocampal BDNF signaling. There are, however, many other possible explanations since the mechanism of 5-HT_6_R ligand cognition enhancement remains elusive. Another proposed hypothesis postulates that agonists activate 5-TH_6_ receptors located directly on cholinergic and/or glutamatergic neurons, while antagonists act probably on 5-HT_6_ receptors located on GABAergic interneurons [35].

## 4. Materials and Methods

### 4.1. Chemistry

Materials. All organic reagents were purchased from Merck and Combi-Blocks and were used without purification. Solvents and inorganic reagents were acquired from Chempur. The reaction progress was monitored by TLC on Merck Silica Gel 60 F 254 on aluminum plates. Column chromatography was performed on Merck Silica Gel 60 (0.063–0.200 mm; 70–230 mesh ASTM).

Analytical methods. UPLC/MS analysis was performed on a Waters TQD spectrometer combined with UPLC Acquity H-Class with a PDA eLambda detector. A Waters Acquity UPLC BEH C18 1.7 μm 2.1 × 50 mm chromatographic column was used at 40 °C, 0.3 mL/min flow rate, and 1.0 μL injection volume (the samples were dissolved in LCMS grade acetonitrile, typically at a concentration of 0.1–1 mg/mL prior to injection). All mass spectra were recorded under electrospray ionization in positive mode (ESI+) and chromatograms were recorded with UV detection in the range of 190–300 nm. The gradient conditions used were: 80% phase A (water +0.1% formic acid) and 20% phase B (acetonitrile +0.1% formic acid) to 100% phase B (acetonitrile +0.1% formic acid) at 3.0 min, kept for 3.5 min, then to initial conditions until 4.0 min, and kept for an additional 2.0 min. Total time of analysis was 6.0 min.

Purity analysis. ^1^H and ^13^C NMR spectra were recorded on a Bruker Avance III HD 500 NMR spectrometer. All samples were dissolved in DMSO-*d*_6_ with TMS as the internal standard. The spectral data of the compounds refer to their free bases or salts.

All presented compounds were of at least 95% purity as determined by LCMS. Syntheses and characterization details for intermediate products and final compounds as well as the spectral data for all compounds are included in the Supplementary Materials.

Software. Marvin Sketch was used to draw the chemical structures, substructures, and reactions, Marvin 19.8.0, ChemAxon. Instant JChem was used for structure searching and chemical database access, Instant JChem 20.20.0, ChemAxon (www.chemaxon.com). Mnova was used to visualize, process, analyze, and report the ^1^H and ^13^C NMR spectra. Mendeley was used for citations (Mendeley Desktop 1.19.4, www.mendeley.com).

General procedure 1 for the synthesis of substituted N-skatyltryptamines (**1**–**23**):

To a solution of appropriate tryptamine (2.85 mmol) in 10 mL of methanol there was added a substituted aldehyde (3 mmol) in one portion. The formation of imine was monitored by TLC. After completion of the reaction, NaBH_4_ (3.3 mmol) was added in small portions. The mixture was left overnight and then tested by TLC or LCMS. When no imine was observed, 20 mL of water was added and the product was extracted three times with 20 mL of ethyl acetate or chloroform. The organic phases were combined, washed two times with 20 mL of water and once with brine, dried over anhydride magnesium sulfate, and concentrated on a rotavap. The final product was purified via flash chromatography using ethyl acetate:methanol:triethylamine 9:1:0.03 (*v*/*v*/*v*).

### 4.2. In Vitro Pharmacology

#### 4.2.1. Radioligand Binding Assay

Cell Culture. HEK293 cells (ATCC) with the stable expression of human serotonin 5-HT_1A_R, 5-HT_6_, and 5-HT_7b_R or dopamine D_2L_R (obtained using of Lipofectamine 2000, Invitrogen) or CHO-K1 cells with plasmid containing the sequence coding for the human serotonin 5-HT_2A_ receptor (PerkinElmer) were maintained at 37 °C in a humidified atmosphere with 5% CO_2_ and were grown in Dulbecco’s modified Eagle’s medium containing 10% dialyzed fetal bovine serum and 500 μg/mL G418 sulfate. For the membrane preparations, cells were subcultured into 150 cm^2^ cell culture flasks, grown to 90% confluence, washed twice with phosphate buffered saline (PBS) prewarmed to 37 °C, pelleted by centrifugation (200 g) in PBS containing 0.1 mM EDTA and 1 mM dithiothreitol, and stored at −80 °C.

General. To determine affinity of all synthesized compounds for following receptors: 5-HT_1A_, 5-HT_2A_, 5-HT_6_, 5-HT_7_, and D_2_ radioligand binding assays were conducted. The assays were performed via the displacement of the respective radioligands from the cloned human receptors, all stably expressed in HEK-293 cells (except for 5-HT_2A_, which was expressed in CHO cells). The experiments were conducted using 1.5 nM [^3^H]-8-OH-DPAT (135.2 Ci/mmol) for 5-HT_1A_R, 2 nM [^3^H]-ketanserin (53.4 Ci/ mmol) for 5-HT_2A_R, 2 nM [^3^H]-LSD (83.6 Ci/mmol) for 5-HT_6_R, 0.6 nM [^3^H]-5-CT (39.2 Ci/mmol) for 5-HT_7_R, and [^3^H]-raclopride (74.4 Ci/mmol) for D_2_R (PerkinElmer, USA). Non-specific binding was defined using 10 mM of 5-HT in the 5-HT_1A_R and 5-HT_7_R binding experiments, whereas 20 mM of mianserin, 10 mM of methiothepine, or 1 mM of (+)-butaclamol was used in the 5-HT_2A_R, 5-HT_6_R, and D_2L_R assays, respectively. Each compound was tested in triplicate at 7–8 concentrations (10^−11^–10^−4^ M). The inhibition constants (*K*_i_) were calculated using the Cheng–Prusoff equation [36] and the results were expressed as the means of at least two independent experiments.

#### 4.2.2. D_2_R Functional Assay

*HEK293* cell line with stable expression of human D_2_ (prepared with the use of Lipofectamine 2000) was maintained at 37 °C in a humidified atmosphere with 5% CO_2_ and was grown in Dulbecco’s modified Eagle medium containing 10% dialyzed fetal bovine serum and 500 µg/mL G418 sulfate. For functional experiments, cells were subcultured in 25 cm^2^ flasks, grown to 90% confluence, washed twice with prewarmed to 37 °C phosphate buffered saline (PBS), and centrifuged for 5 min (160× *g*). The supernatant was aspirated, and the cell pellet was resuspended in stimulation buffer (1 × HBSS, 5 mM HEPES, 0.5 mM IBMX, 0.1% BSA).

The functional properties of compounds were evaluated using the LANCE Ultra cAMP Detection Kit (PerkinElmer). D_2_ receptors in HEK293 cells are coupled to G_i_ subtype and decreased cAMP production. Cells were stimulated with 1 μM of forskolin (EC_90_). Each compound was tested in triplicate at eight concentrations (10^−11^–10^−4^ M).

For quantification of cAMP levels, cells (5 µL) were incubated with 5 µL mixture of compounds (tested ligand and forskolin with 100 nM quinpirole for antagonist binding mode) for 30 min at room temperature in 384-well white opaque microtiter plate (PerkinElmer). After incubation, the reaction was stopped and cells were lysed by the addition of 10 µL working solution (5 µL Eu-cAMP and 5 µL ULight-anti-cAMP). The assay plate was incubated for 1 h at room temperature. Time-resolved fluorescence resonance energy transfer (TR-FRET) signal was detected by an Infinite M1000 Pro (Tecan) using instrument settings from the LANCE Ultra cAMP Detection Kit manual.

#### 4.2.3. 5-HT_6_R Functional Assays

The properties of compounds to inhibit cAMP production induced by a 5-HT_6_R agonist 5-CT (1000 nM) was evaluated. Compounds were tested in triplicate at eight concentrations (10^−11^–10^−4^ M). The level of cAMP was measured using frozen recombinant 1321N1 cells expressing the Human Serotonin 5-HT_6_R (PerkinElmer). Total cAMP was measured using the LANCE cAMP Detection Kit (PerkinElmer), according to the manufacturer’s instructions. For quantification of cAMP levels, 2000 cells/well (5 mL) were incubated with a mixture of compounds (5 mL) for 30 min at room temperature in a 384-well white opaque microtiter plate. After incubation, the reaction was stopped and cells were lysed by the addition of 10 mL of working solution (5 mL Eu-cAMP and 5 mL ULight-anti-cAMP) for 1 h at room temperature. Time-resolved fluorescence resonance energy transfer (TR-FRET) was detected by an Infinite M1000 Pro (Tecan) using instrument settings from the LANCE cAMP Detection Kit manual. *K*_b_ values were calculated from the Cheng–Prusoff equation specific for the analysis of functional inhibition curves: *K*_b_ = IC_50_/(1 + A/EC_50_) where A represents the agonist concentration; IC_50_ is the concentration of antagonist producing a 50% reduction in the response to agonist; and EC_50_ is the agonist concentration that causes half of the maximal response. The agonistic properties of compounds were determined with an analogous procedure, but without stimulation with 5-CT [10,36].

### 4.3. Molecular Modeling

Molecular Docking. The 5-HT_6_R homology models built on the β2 receptor template (PDB ID: 4LDE) were used in this study [37]. The structure of D_2_R in complex with antagonist risperidone (PDB code6CM4) was retrieved from the Protein Data Bank.

The three-dimensional structures of the ligands were obtained using LigPrep and the appropriate ionization states at pH = 7.4 ± 1.0 were assigned using Epik. The Protein Preparation Wizard was used to assign the bond orders and appropriate amino acid ionization states and to check for steric clashes. The receptor grid was generated (OPLS3 force field) by centering the grid box with a size of 12 Å on the D3.32 side chain. Docking was performed by the quantum-polarized ligand docking (QPLD) procedure implemented in the Schrodinger Suite. QPLD involves the QM-derived ligand atomic charges in the protein environment at the B3PW91 level with conjunction with the ccpVTZ basis set for Cl and Br and the cc-pVTZ-pp basis set for I-containing ligands. Only the best ten poses per ligand returned by the procedure were considered.

### 4.4. In Vitro ADMETox Studies

Materials. The following compounds: caffeine, carbonyl cyanide 3-chlorophenylhydrazone, doxorubicin, ketoconazole, norfloxacin and quinidine were used in this study as the references and purchased from Sigma-Aldrich (St. Louis, MO, USA).

#### 4.4.1. PAMPA

The permeability of compounds was determined by the Pre-coated PAMPA Plate System Gentest™ (Corning, Tewksbury, MA, USA) similarly to a previously described methodology [38,39]. Caffeine (well-permeable reference), norfloxacin (low-permeable reference), and tested compounds were dissolved in PBS buffer (pH = 7.4) and added to the donor wells (300 µL/well) in a final concentration of 200 µM. PBS in a portion of 200 µL/well was added to the wells. Experiments were conducted in triplicate. Incubation of the PAMPA Plate System took 5 h at room temperature. After that time, 50 µL was aspirated from each well and diluted with 50 µL of solution of internal standard (IS). UPLCMS analyses allowed us to estimate the compounds’ concentration in the acceptor and donor wells. The permeability coefficients were determined according to formulas provided by the manufacturer [40].

#### 4.4.2. Metabolic Stability

The murine liver microsomes (MLMs) were purchased from Sigma-Aldrich (St. Louis, MO, USA). To determine metabolic pathways, the tested compounds (50 µM) were incubated with 100 mM Tris-HCl buffer at 37 °C with MLMs (1 mg/mL) and NADPH Regeneration System for 2 h. Reactions were quenched with cold, pure methanol and mixtures were centrifuged 14,000 g for 15 min. The supernatants were analyzed using LCMS. The additional MS ion fragmentation of the product and the substrate were performed to determine the most probable structure of the metabolite.

In silico prediction of metabolic biotransformations was performed by MetaSite 6.0.1 (Molecular Discovery Ltd., Hertfordshire, UK) [41]. The most probable metabolic sites of the tested compounds were determined by a computational liver model of metabolism.

#### 4.4.3. CYP450 Inhibition

The experiments were provided using commercially available luminescent CYP3A4 P450-Glo™ and CYP2D6 P450-Glo™ tests purchased from Promega (Madison, WI, USA). The enzymatic reactions were conducted in polystyrene, flat-bottom Nunc™ MicroWell™ 96-well microplates (Thermo Scientific, Waltham, MA, USA). The assays were carried out according to the procedures provided by the manufacturer, as described previously [38,41,42]. Compounds were tested in triplicate at the final concentrations in a range from 0.01 to 25 µM for both isoforms of CYP450. The references of CYP3A4 and CYP2D6 inhibitors (ketoconazole and quinidine, respectively) were tested in a range from 0.001 to 10 µM. Tested compounds were incubated in 100 mM Tris-HCl buffer separately with CYP3A4 and CYP2D6 membranes and the NADPH Regeneration System for 30 min at room temperature in triplicate. The bioluminescent signal was measured after the addition of the Luciferin Detection Reagent by using a microplate reader EnSpire PerkinElmer (Waltham, MA, USA). Both reagents (NADPH Regeneration System and Luciferin Detection Reagent) were purchased from Promega (Madison, WI, USA).

#### 4.4.4. Hepatotoxicity Assay

To estimate the hepatotoxicity of compounds, the hepatoma HepG2 (ATCC^®^ HB-8065TM) cell line was used according to previously described protocols [39,42]. The CellTiter 96^®^ AQueous Non-Radioactive Cell Proliferation Assay was obtained from Promega (Madison, WI, USA). The compounds were tested in quadruplicate at four concentrations (1–100 µM) for 72 h.

### 4.5. Behavioral Tests

Animals. Male CD1 mice (Charles River, Germany) weighing 20–25 g at the time of arrival were used in behavioral experiments. Animals were kept under standard laboratory conditions (12:12 light: dark cycle, 22 ± 2 °C) with free access to food and water. Animal welfare has been regularly controlled by a veterinarian and animal welfare committee. After two weeks of acclimatization and handling, the experiments began. Experimental groups consisted of four to 10 animals, depending on the procedure. Drugs were administered intraperitoneally (i.p.) at a volume of 10 mL/kg. Experimental assessments were performed by an observer who was blinded to the treatment conditions. All procedures were conducted in accordance with the European Communities Council Directive of 22 September 2010 (2010/63/EU) and Polish legislation acts concerning animal experimentation and were approved by the II Local Ethics Committee by the Maj Institute of Pharmacology, Polish Academy of Sciences in Krakow (272/2019).

Drugs. MK-801 was purchased from Tocris Bioscience, Bristol, UK. MK-801 was dissolved in 0.9% NaCl. Compounds **15** and **18** were dissolved in 0.9% NaCl. All compounds were administered intraperitoneally (i.p.) in a volume of 10 mL/kg. Vehicle-treated animals received appropriate solvents. Vehicle was administered to animals in any case when drug administration was omitted (e.g., control or MK-801-treated groups).

#### 4.5.1. Novel Object Recognition Test

This procedure was adapted from Nilsson et al. (2007) [43] and performed as described in a previous paper by Cieslik et al. (2018) [44]. Habituation, training, and test trials were performed in a black plastic rectangular arena (40 × 30 × 35 cm) illuminated with a light intensity of 335 lux. During the habituation trial (two consecutive days), each animal was allowed to explore the arena for 10 min. The next day, during the training trial (T1), mice were placed in the arena and presented with two identical objects (red glass cylinder; 6.5 cm in diameter and 4.5 cm high) for 5 min. After 1 h, animals were placed back into the arena for a 5 min test trial, during which one of the previously presented familiar objects was replaced with a novel object (a transparent glass elongated sphere-like object with an orange cap; 5.5 cm in diameter and 8.5 cm high). Time spent exploring (i.e., sniffing or touching) the familiar (T_familiar_) and novel (T_novel_) objects was measured by a trained observer, and the recognition index [%] was calculated for each mouse [(T_novel_ − T_familiar_)/(T_familiar_ + T_novel_)] × 100. Compounds were administered 30 min before MK-801 (0.3 mg/kg), which was administered 30 min before training trial.

#### 4.5.2. MK-801-Induced Hyperactivity

The locomotor activity was recorded individually for each animal in locomotor activity cages (according to Rorick-Kehn et al., 2007a,b) [45,46], with modifications (Wieronska et al., 2012) [47]. The mice were placed individually into activity cages (13 × 23 × 15 cm; Opto-M3; Columbus Instruments) for an acclimatization period of 30 min; then they were injected i.p. with compound **15** (0.5, 1, 3 mg/kg) or compound **18** (0.05, 0.1, 0.5, 1, 3 mg/kg) and placed again in the same cages. After 30 min, all of the mice were injected i.p. with MK-801 at 0.35 mg/kg and once again placed in the same cage. From then on, the ambulation scores were counted for 60 min. All of the groups were compared with the MK-801 control group. The experiment also included a control group treated with NaCl only.

#### 4.5.3. Locomotor Activity of Mice

The locomotor activity was recorded individually for each animal in the OPTO-M3 locomotor activity cages described above. Each cage was surrounded with an array of photocell beams. Interruptions of photo beams resulted in horizontal activity defined as ambulation scores. Mice were placed separately into activity cages for an acclimatization period of 30 min, then were injected i.p. with compound **15** (0.5, 1, 3 mg/kg) compound **18** (0.05, 0.1, 0.5, 1, 3 mg/kg. After a further 30 min, they were injected with saline (10 mL/kg). From this point on, the ambulation scores were measured for 60 min.

Data Analysis. The data are presented as the means ± SEM. Statistical analysis of the data was performed using Prism 8. One-way ANOVA, followed by the Newman–Keuls post-hoc comparison test, was used in the analysis of the dose-dependent studies of compounds **15** and **18**. A *p*-value of < 0.05 was considered as statistically significant.

## 5. Conclusions

A further optimization of substituents might yield compounds with enhanced binding/function for the discussed receptors and more optimal profile. On the other hand, the therapeutic potential of *N*-skatyltryptamines seems doubtful due to the discovered hepatotoxicity. A clogP = 3.80 (ChemAxon) was calculated for the unsubstituted derivative **1**, placing the lipophilicity at the border of the preferred range. The discovered series may serve as a pool of new tool compounds with useful receptor profiles for CNS studies.

## Figures and Tables

**Table 1:** Binding affinities *K*_i_ of the synthesized compounds. Substitutions, which resulted in an increased affinity were marked in the Markush structure with arcs: 5-HT_2A_R—red, 5-HT_6_R—green, D_2_R—blue.

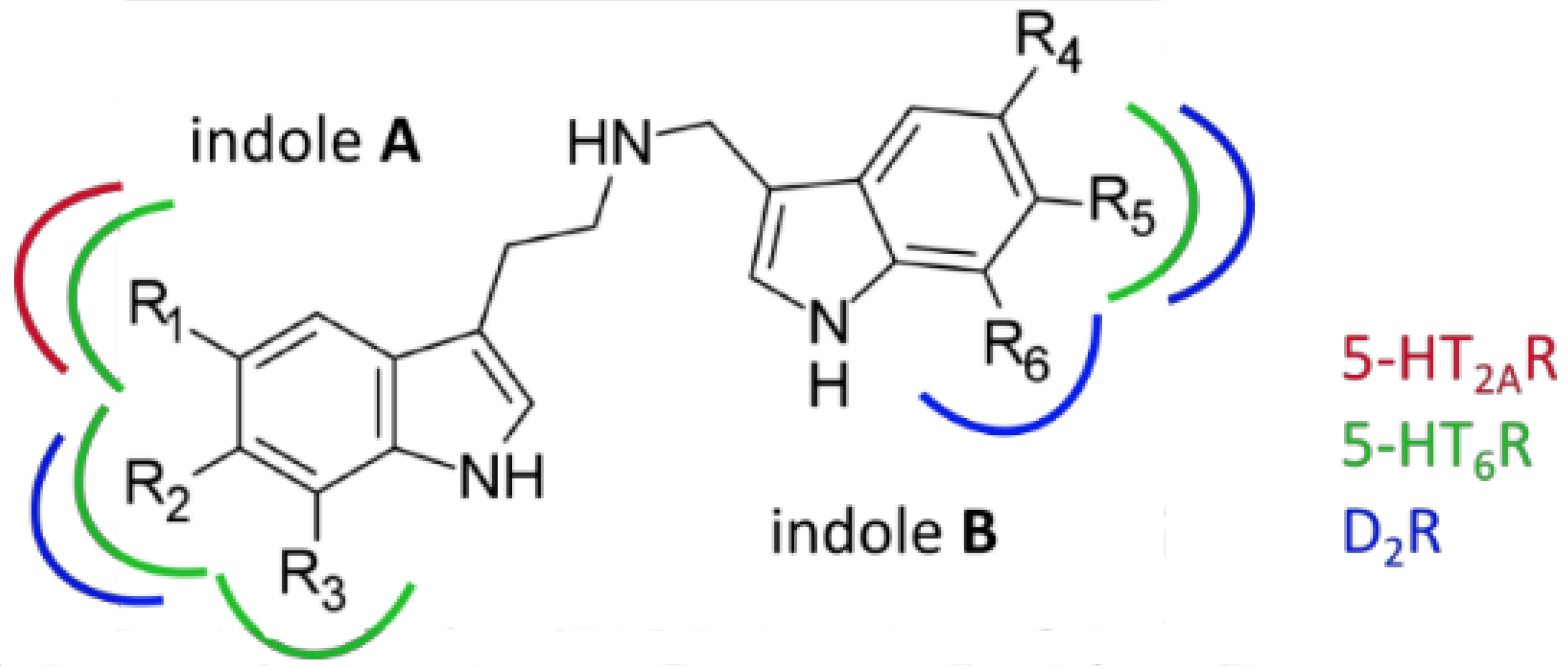											
							p*K*_i_ [nM]				
ID	R1	R2	R3	R4	R5	R6	5-HT_1A_	5-HT_2A_	5-HT_6_	5-HT_7_	D_2_
Trp	H	H	H	-	-	-	7.16	6.51	6.95	7.22	<5.00
5-MeOTrp	MeO	H	H	-	-	-	9.05	7.35	7.92	6.32	6.18
5-Cl-Trp	Cl	H	H	-	-	-	8.80	7.50	8.42	8.80	6.61
**1**	H	H	H	H	H	H	6.82	8.70	7.76	5.54	7.22
**2**	Me	H	H	H	H	H	7.16	7.25	7.89	6.53	<5.00
**3**	OMe	H	H	H	H	H	7.81	8.08	7.93	7.25	6.49
**4**	Cl	H	H	H	H	H	n.d.	n.d.	7.97	n.d.	6.61
**5**	H	Cl	H	H	H	H	6.48	6.93	7.78	5.80	7.81
**6**	H	H	F	H	H	H	6.56	7.42	7.77	5.99	6.53
**7**	H	H	Cl	H	H	H	6.29	7.20	7.88	5.60	5.93
**8**	H	H	Br	H	H	H	6.22	7.11	7.79	5.69	6.52
**9**	H	H	H	OH	H	H	6.54	6.71	6.65	6.29	6.12
**10**	H	H	H	F	H	H	7.31	6.46	7.39	6.32	6.07
**11**	H	H	H	Cl	H	H	7.17	6.49	7.29	6.42	6.68
**12**	H	H	H	I	H	H	7.31	6.63	7.34	6.29	6.68
**13**	H	H	H	H	F	H	6.87	6.59	7.65	5.74	7.52
**14**	H	H	H	H	Cl	H	6.97	7.33	8.05	5.80	7.91
**15**	H	H	H	H	Br	H	7.01	6.39	7.99	6.33	7.54
**16**	H	H	H	H	H	F	6.25	6.91	7.50	5.89	8.04
**17**	H	H	H	H	H	Cl	7.55	6.41	7.44	6.97	8.29
**18**	H	H	H	H	H	Br	7.52	6.40	7.35	6.88	8.26
**19**	H	H	H	H	H	OBn	7.13	6.64	7.52	5.95	6.39
**20**	OMe	H	H	H	H	Cl	8.10	7.51	8.01	7.53	6.63
**21**	OMe	H	H	H	Cl	H	7.73	7.76	8.28	7.09	6.81
**22**	H	Cl	H	H	H	Cl	6.25	6.39	7.44	5.88	6.30

n.d.—not determined.

**Table 2:** Determined EC_50_ and *K*_b_ values for the selected compounds.

ID	R_1_	R_2_	R_3_	R_4_	R_5_	R_6_	5-HT_6_ EC_50_	5-HT_6_ *K*_b_	D_2_ *K*_b_
							[nM]		
**1**	H	H	H	H	H	H	n.d.	151	n.d.
**5**	H	Cl	H	H	H	H	157	136	104
**13**	H	H	H	H	F	H	n.d.	30	91
**14**	H	H	H	H	Cl	H	n.d.	112	290
**15**	H	H	H	H	**Br**	H	n.d.	**131**	**109**
**17**	H	H	H	H	H	Cl	n.d.	234	215
**18**	H	H	H	H	H	**Br**	**77**	10,000	**167**
**21**	OMe	H	H	H	H	Cl	22	10,000	n.d.
**22**	OMe	H	H	H	Cl	H	26	n.d.	n.d.

n.d.–not determined.

**Table 3:** Permeability coefficients for the tested compounds.

Compound	PAMPA Pe * [10^−6^ cm/s] ± SD
Norfloxacin	0.56 ± 0.13
Caffeine	15.1 ± 0.40
**5**	7.09 ± 1.24
**14**	8.34 ± 1.33
**15**	2.42 ± 0.92
**17**	8.42 ± 0.27
**18**	3.90 ± 0.56

* tested in triplicate.

**Table 4:** Molecular masses of the tested compound and retention times and molecular mass of the metabolite and the most probable metabolic pathways of the hit compounds.

Compound	Molecular Mass (*m*/*z*)	Retention Time (min)	Molecular Mass of the Metabolite (*m*/*z*)	Metabolic Pathway
**5**	324.25	4.17	**M1** 340.26	hydroxylation
**14**	324.31	4.054.38	**M1** 340.26 **M2** 340.33	hydroxylation hydroxylation
**15**	370.13	4.124.34	**M1** 386.15 **M2** 384.09	hydroxylation hydroxylation
**17**	324.25	3.954.27	**M1** 340.26 **M2** 340.26	hydroxylation hydroxylation
**18**	368.14	4.004.27	**M1** 384.09 **M2** 386.08	hydroxylation hydroxylation

**Table 5:** Effect of compound **15** and **18** on the spontaneous activity of mice.

Compounds	Doses (mg/kg)	Ambulation Scores ± SEM
NaCl	-	100.0 ± 11.81
**15**	0.5	134.9 ± 11.13
**15**	1	124.7 ± 21.75
**15**	3	91.8 ± 24.61
NaCl	-	100.0 ± 8.95
**18**	0.05	113.6 ± 34.00
**18**	0.1	131.8 ± 19.50
**18**	0.5	116.7 ± 13.23
**18**	1	85.31 ± 15.86
**18**	3	87.79 ± 20.89

## Supplementary Materials

The following are available online: Syntheses and characterization details for final products, ^1^H and ^13^C NMR spectra, LC-MS spectra, conditions of radioligand binding assay, predicted metabolites, and retention times of tested compounds and its metabolites.

## Sample Availability

Samples of compounds **1**–**22** are available from the authors.
