# Supplementary material for: N-Skatyltryptamines—Dual 5-HT6R/D2R Ligands with Antipsychotic and Procognitive Potential
Source: Molecules. 2021 Jul 29;26(15):4605. doi: 10.3390/molecules26154605 (PMC8347595; doi:10.3390/molecules26154605)
Supplement: Supplementary file 1 [file molecules-26-04605-s001.zip › molecules-1263641-supplementary.pdf]

Supplementary materials

N-Skatyltryptamines – Dual 5-HT$_6$R/D$_2$R Ligands with Antipsychotic and Procognitive Potential

Agata Hogendorf [1], Adam S. Hogendorf [1], Rafał Kurczab [1], Grzegorz Satała [1], Bernadeta Szewczyk [2], Paulina Cieślik [2], Gniewomir Latacz [3], Jadwiga Handzlik [3], Tomasz Lenda [4], Katarzyna Kaczorowska [1], Jakub Staroń [1], Ryszard Bugno [1], Beata Duszyńska [1] and Andrzej J. Bojarski. [1,*]

[1] Department of Medicinal Chemistry, Maj Institute of Pharmacology, Polish Academy of Sciences, 12 Smętna Street, 30-343 Kraków, Poland; agatah@if-pan.krakow.pl (A.H.), ahogen@if-pan.krakow.pl (A.S.H.); kurczab@if-pan.krakow.pl (R.K.); satala@if-pan.krakow.pl (G.S.); k.kaczor@if-pan.krakow.pl (K.K.); staron@if-pan.krakow.pl (J.S.); bugno@if-pan.krakow.pl (R.B.); duszyn@if-pan.krakow.pl (B.D.).

[2] Department of Neurobiology, Maj Institute of Pharmacology Polish Academy of Sciences, 12 Smętna Street, 30-343 Kraków, Poland; szewczyk@if-pan.krakow.pl (B.S.); cieslik@if-pan.krakow.pl (P.C.).

[3] Department of Technology and Biotechnology of Drugs, Jagiellonian University Medical College, Medyczna 9, PL 30-688 Kraków, Poland; glatacz@cm-uj.krakow.pl (G.L.); j.handzlik@uj.edu.pl (J.H.).

[4] Department of Neuropsychopharmacology, Maj Institute of Pharmacology, Polish Academy of Sciences, 12 Smętna Street, 30-343 Kraków, Poland; lenda@if-pan.krakow.pl (T.L).

* Correspondence: bojarski@if-pan.krakow.pl

Table of contents

1. Chemical characterization of synthesized compounds 2
2. In vitro pharmacology 56
   2.1 Radioligand binding assay 56
3 Metabolic stability 57

1. Chemical characterization of synthesized compounds.

[2-(1*H*-indol-3-yl)ethyl][(1*H*-indol-3-yl)methyl]amine (1)

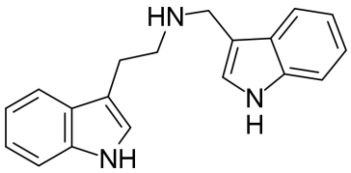

The product was obtained as a white solid. Yield: 48%.
LCMS [M+1] = 290,11 (290,17 calculated).

^{1}H NMR (500 MHz, DMSO-d_6) δ 11.44 (d, *J* = 2.6 Hz, 1H), 11.00 (d, *J* = 2.4 Hz, 1H), 9.29 (d, *J* = 7.1 Hz, 2H), 7.79 (dq, *J* = 7.9, 0.9 Hz, 1H), 7.63 (d, *J* = 2.6 Hz, 1H), 7.55 (dt, *J* = 7.9, 0.9 Hz, 1H), 7.43 (dt, *J* = 8.1, 0.9 Hz, 1H), 7.36 (dt, *J* = 8.2, 0.9 Hz, 1H), 7.20 (d, *J* = 2.4 Hz, 1H), 7.14 (ddd, *J* = 8.1, 7.0, 1.2 Hz, 1H), 7.07 (dtd, *J* = 8.0, 6.9, 1.1 Hz, 2H), 6.98 (ddd, *J* = 8.0, 7.0, 1.0 Hz, 1H), 4.34 (t, *J* = 5.4 Hz, 2H), 3.15 (d, *J* = 8.3 Hz, 4H), 2.50 (p, *J* = 1.8 Hz, 2H).
^{13}C NMR (126 MHz, DMSO-d_6) δ 136.24, 135.94, 135.88, 127.41, 126.75, 123.16, 121.57, 121.14, 119.14, 118.67, 118.42, 118.18, 111.70, 111.53, 109.51, 104.95, 46.38, 41.09, 25.50, 21.67.

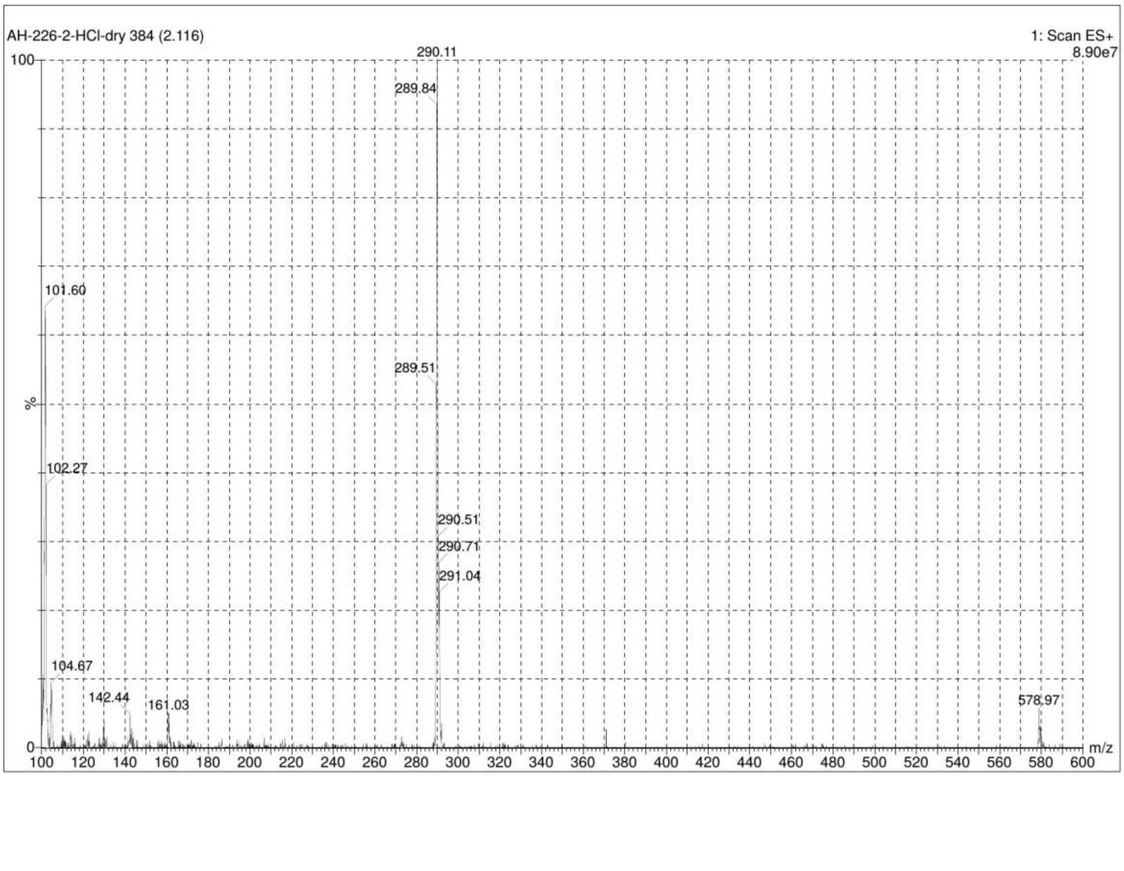

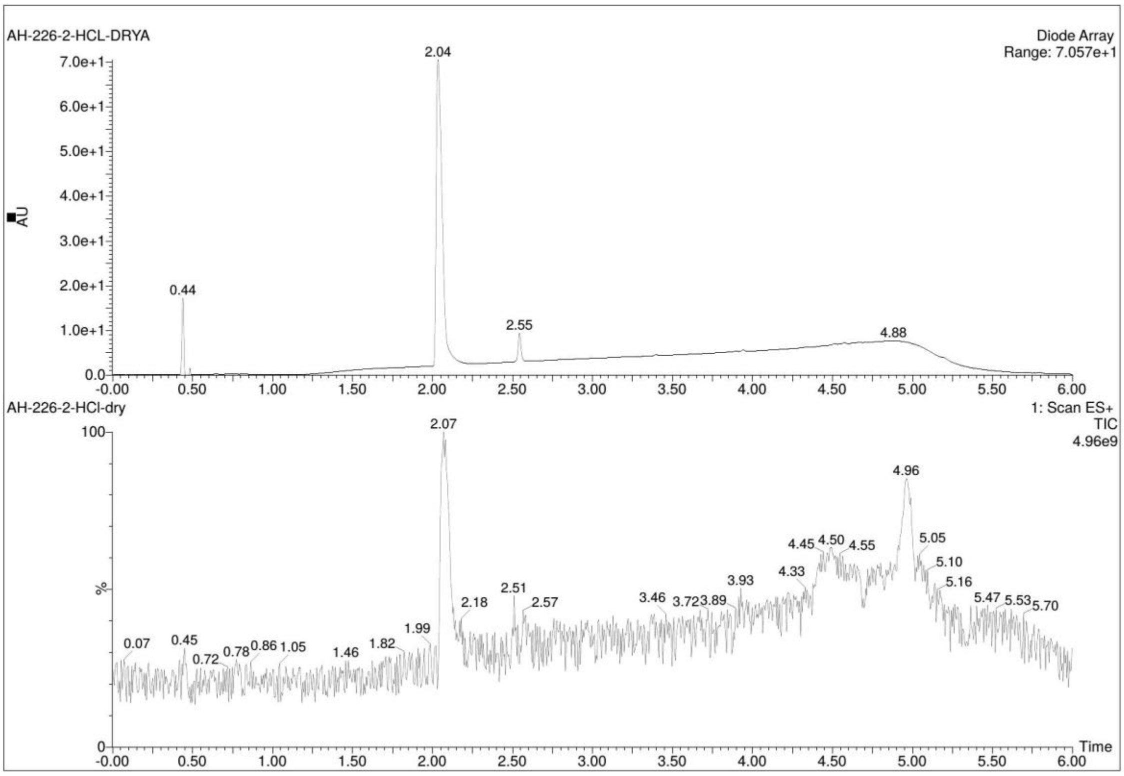
AH-226-2-HCL-DRYA
Diode Array
Range: 7.057e+1
AU
AH-226-2-HCl-dry
1: Scan ES+
TIC
4.96e9
%
Time

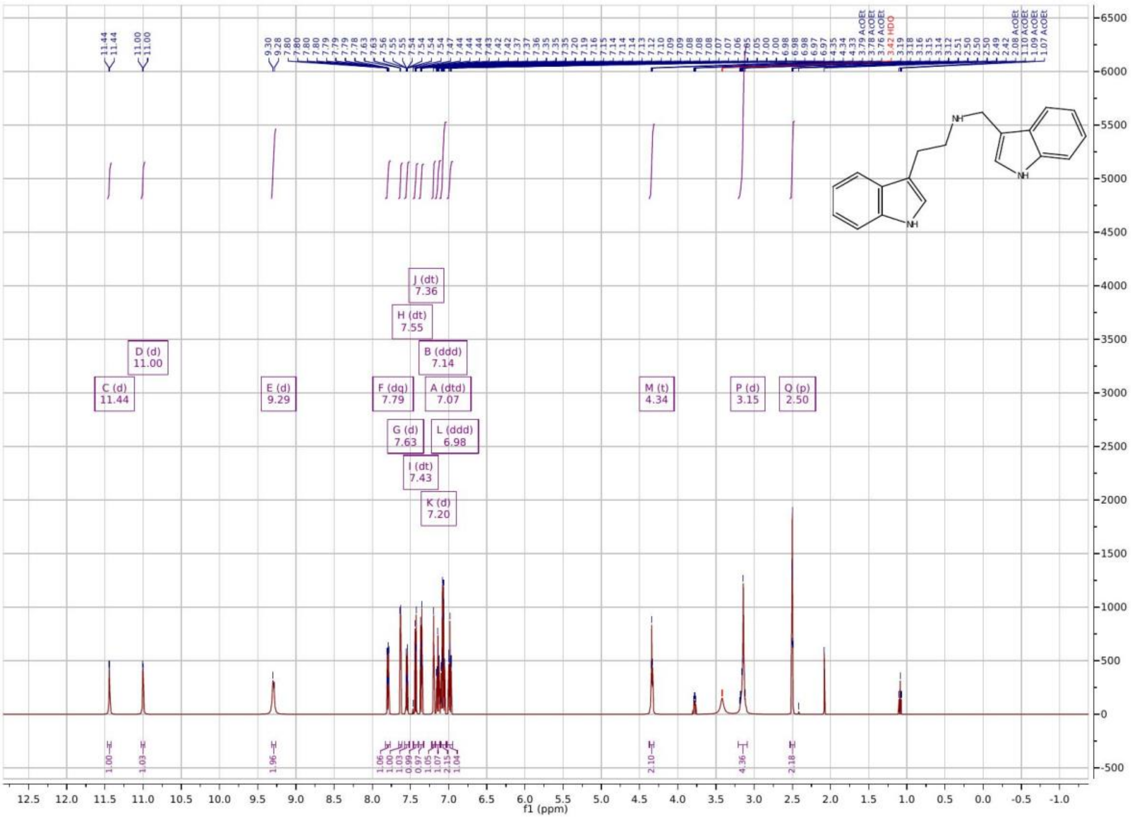
C (d)
11.44
D (d)
11.00
E (d)
9.29
F (dq)
7.79
G (d)
7.63
H (dt)
7.55
I (dt)
7.43
J (dt)
7.36
K (d)
7.20
B (ddd)
7.14
A (dtd)
7.07
L (ddd)
6.98
M (t)
4.34
P (d)
3.15
Q (p)
2.50
NH
f1 (ppm)

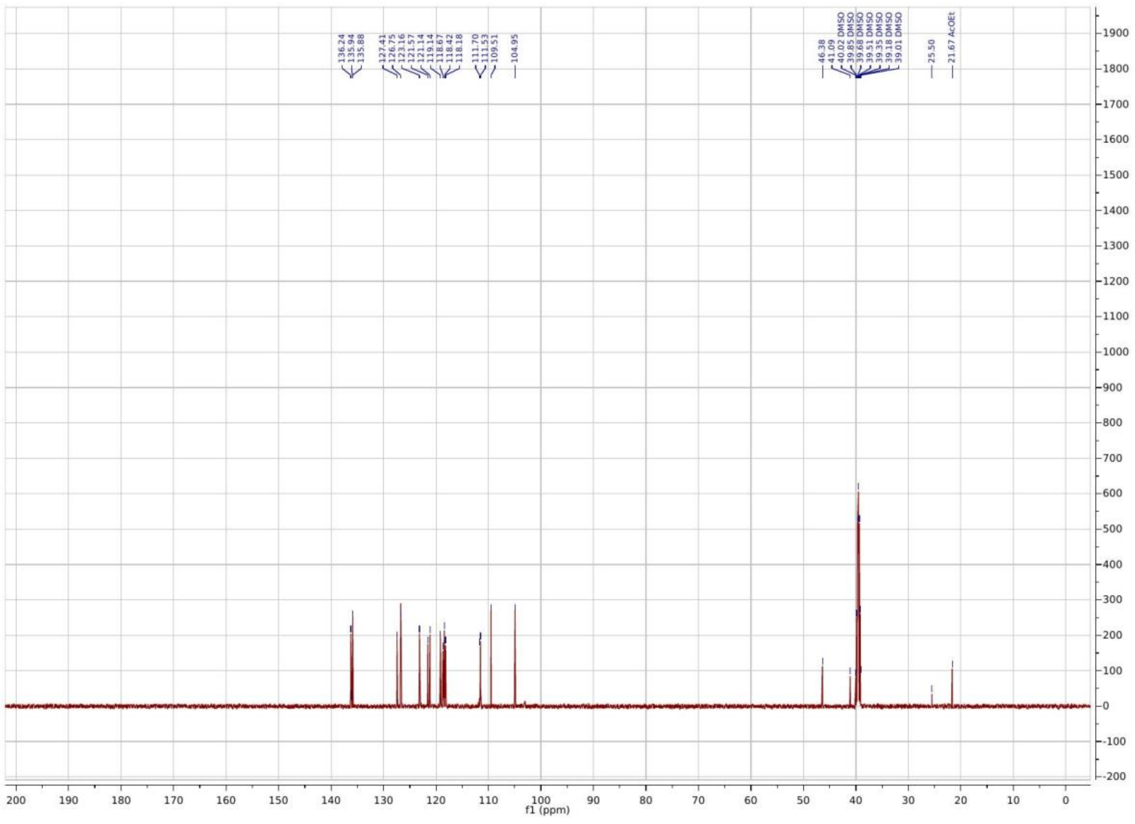

[(1*H*-indol-3-yl)methyl][2-(5-methyl-1*H*-indol-3-yl)ethyl]amine (2)

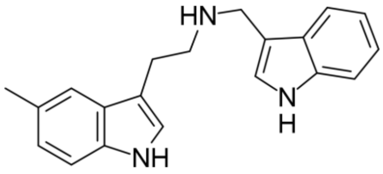

The product was obtained as a beige solid. Yield: 50%.
LCMS [M+1] = 304,09 (304,18 calculated).

^{1}H NMR (500 MHz, DMSO-d_6) δ 11.43 (s, 1H), 10.83 (s, 1H), 7.76 (dt, *J* = 7.9, 0.9 Hz, 1H), 7.55 (d, *J* = 2.6 Hz, 1H), 7.44 (dt, *J* = 8.1, 0.9 Hz, 1H), 7.27 – 7.21 (m, 2H), 7.18 – 7.11 (m, 2H), 7.07 (ddd, *J* = 7.9, 7.0, 1.0 Hz, 1H), 6.90 (dd, *J* = 8.4, 1.6 Hz, 1H), 4.37 (s, 2H), 3.20 – 3.13 (m, 2H), 3.04 (dd, *J* = 9.9, 6.4 Hz, 2H), 2.36 (s, 3H).
^{13}C NMR (126 MHz, DMSO-d_6) δ 164.79, 135.94, 134.63, 127.23, 126.95, 126.88, 126.78, 123.26, 122.76, 121.60, 119.17, 118.58, 117.66, 111.76, 111.26, 108.95, 105.10, 46.40, 41.15, 21.74, 21.27.

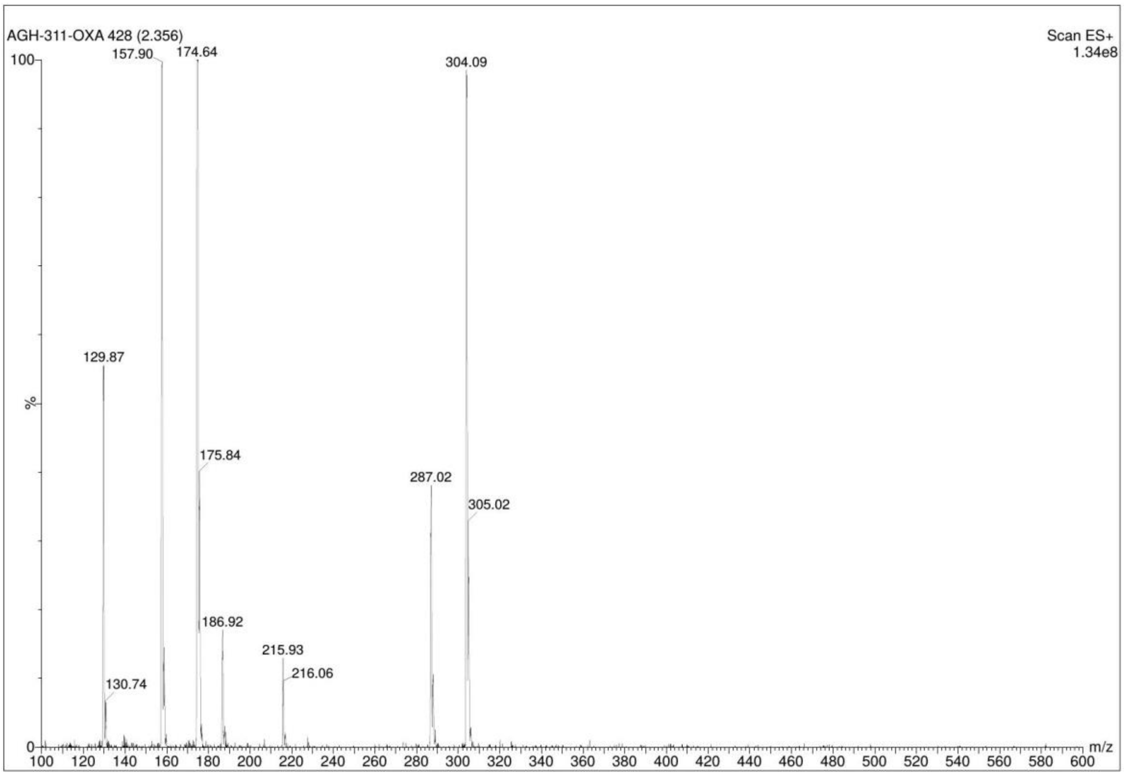

AGH-311-OXA 428 (2.356)
Scan ES+
1.34e8
100
%
0
157.90
174.64
304.09
129.87
175.84
287.02
305.02
186.92
215.93
216.06
130.74
m/z
100 120 140 160 180 200 220 240 260 280 300 320 340 360 380 400 420 440 460 480 500 520 540 560 580 600

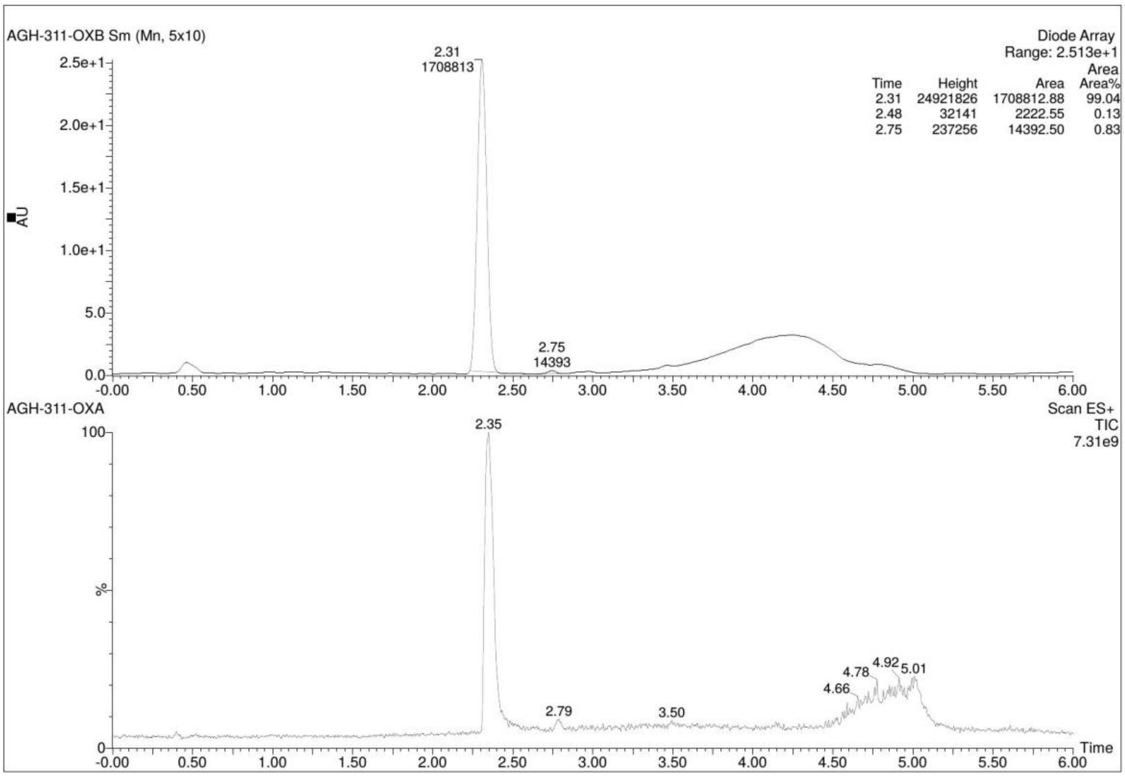

AGH-311-OXB Sm (Mn, 5x10)
Diode Array
Range: 2.513e+1
Time Height Area Area%
2.31 24921826 1708812.88 99.04
2.48 32141 2222.55 0.13
2.75 237256 14392.50 0.83
2.31
1708813
2.75
14393
AU
2.5e+1 2.0e+1 1.5e+1 1.0e+1 5.0 0.0
AGH-311-OXA
Scan ES+
TIC
7.31e9
2.35
2.79
3.50
4.66
4.78
4.92
5.01
%
100
0
-0.00 0.50 1.00 1.50 2.00 2.50 3.00 3.50 4.00 4.50 5.00 5.50 6.00
Time

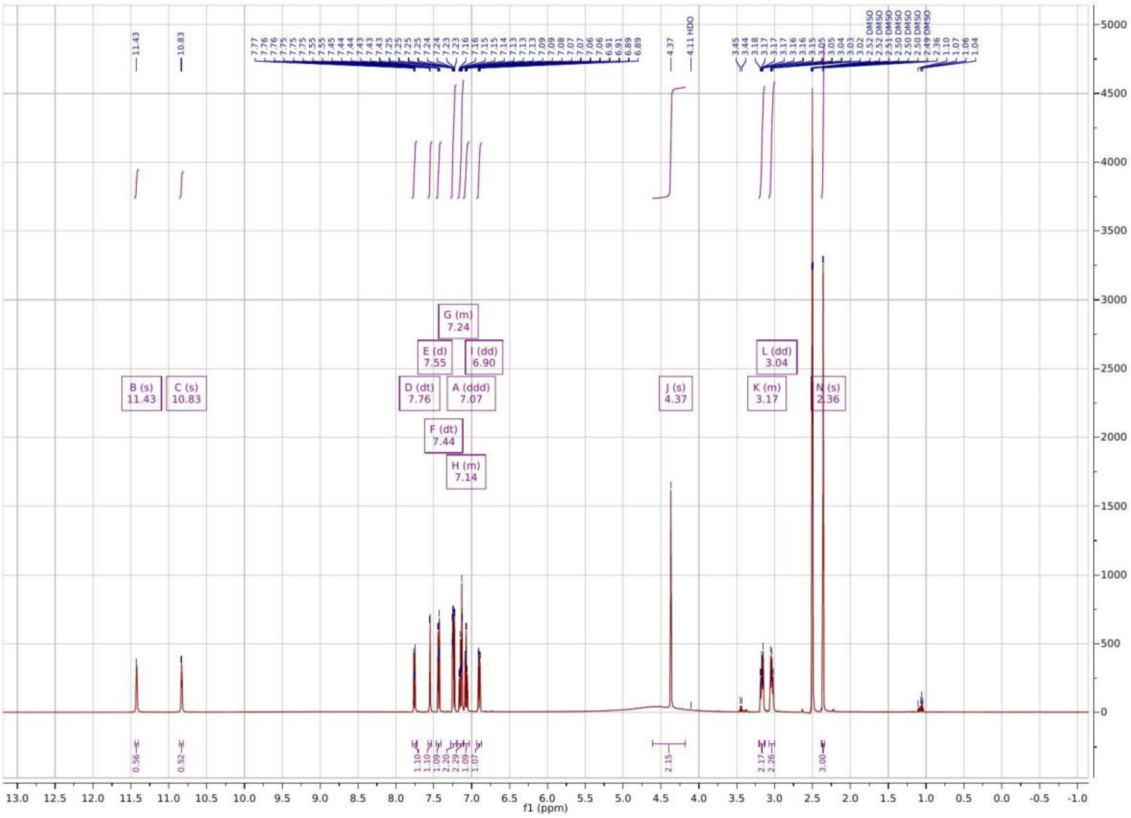

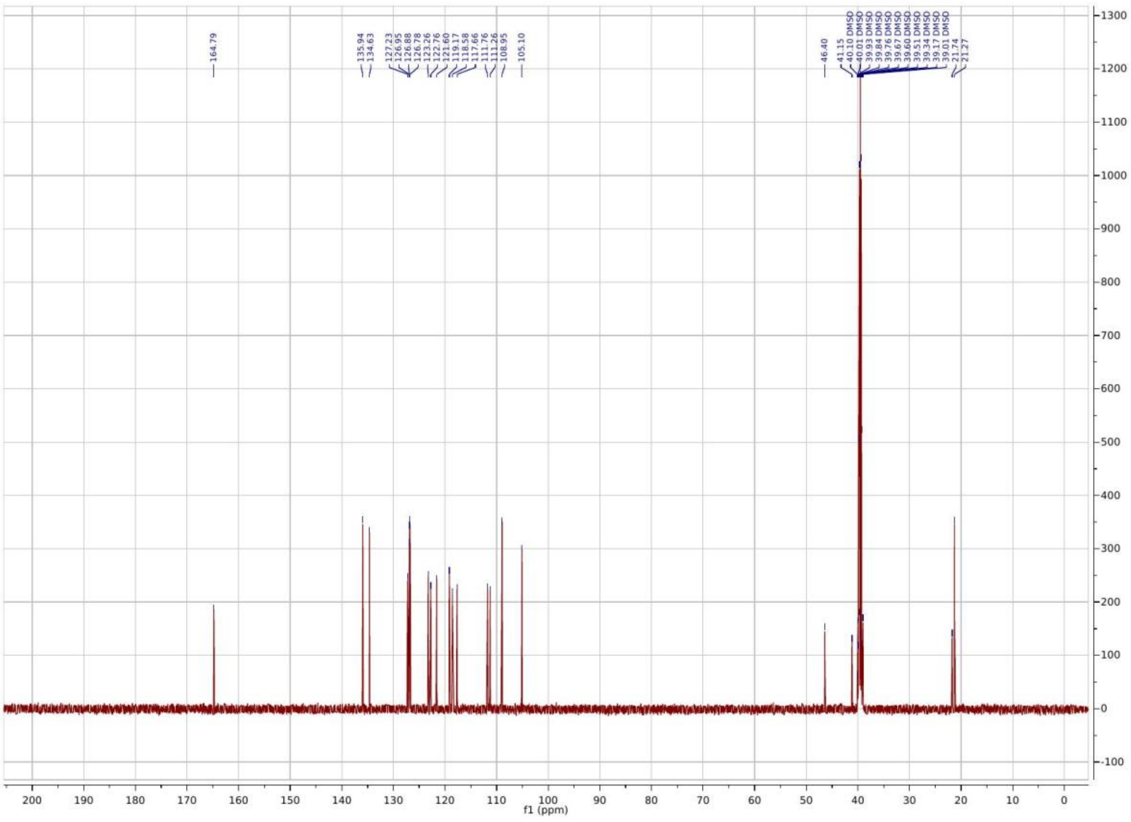

[(1*H*-indol-3-yl)methyl][2-(5-methoxy-1*H*-indol-3-yl)ethyl]amine (3)

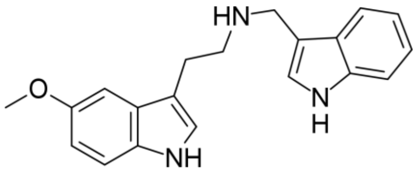

The product was obtained as a off-white solid. Yield: 63%.
LCMS [M+1] = 320,09 (320,18 calculated).

^{1}H NMR (500 MHz, DMSO-d_6) δ 11.42 (s, 1H), 10.81 (s, 1H), 7.75 (dq, *J* = 7.9, 0.9 Hz, 1H), 7.55 (d, *J* = 2.6 Hz, 1H), 7.43 (dt, *J* = 8.1, 0.9 Hz, 1H), 7.24 (d, *J* = 8.7 Hz, 1H), 7.20 – 7.10 (m, 2H), 7.07 (ddd, *J* = 8.0, 7.0, 1.1 Hz, 1H), 7.00 (d, *J* = 2.4 Hz, 1H), 6.73 (dd, *J* = 8.8, 2.4 Hz, 1H), 4.37 (s, 2H), 3.74 (s, 3H), 3.21 – 3.14 (m, 2H), 3.04 (dd, *J* = 9.8, 6.5 Hz, 2H).
^{13}C NMR (126 MHz, DMSO-d_6) δ 164.82, 153.13, 135.95, 131.38, 127.23, 127.06, 126.78, 123.86, 121.61, 119.18, 118.57, 112.23, 111.76, 111.31, 109.25, 105.10, 99.97, 55.38, 46.33, 41.16, 21.76.

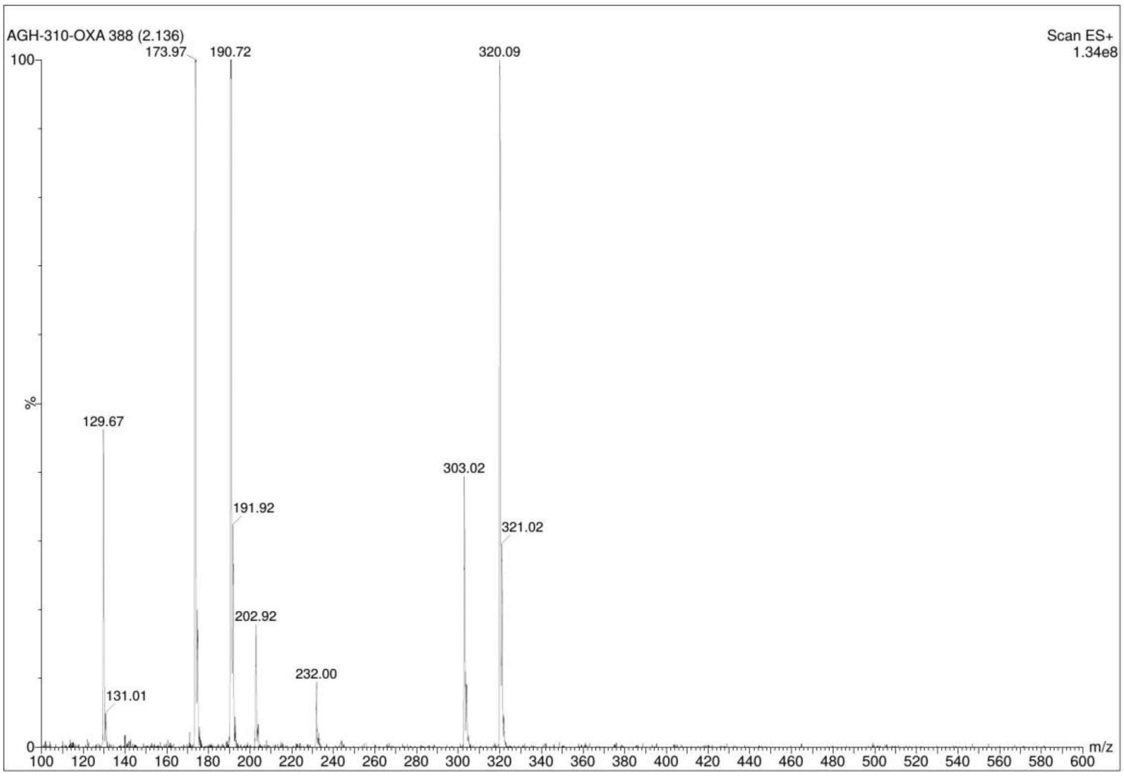

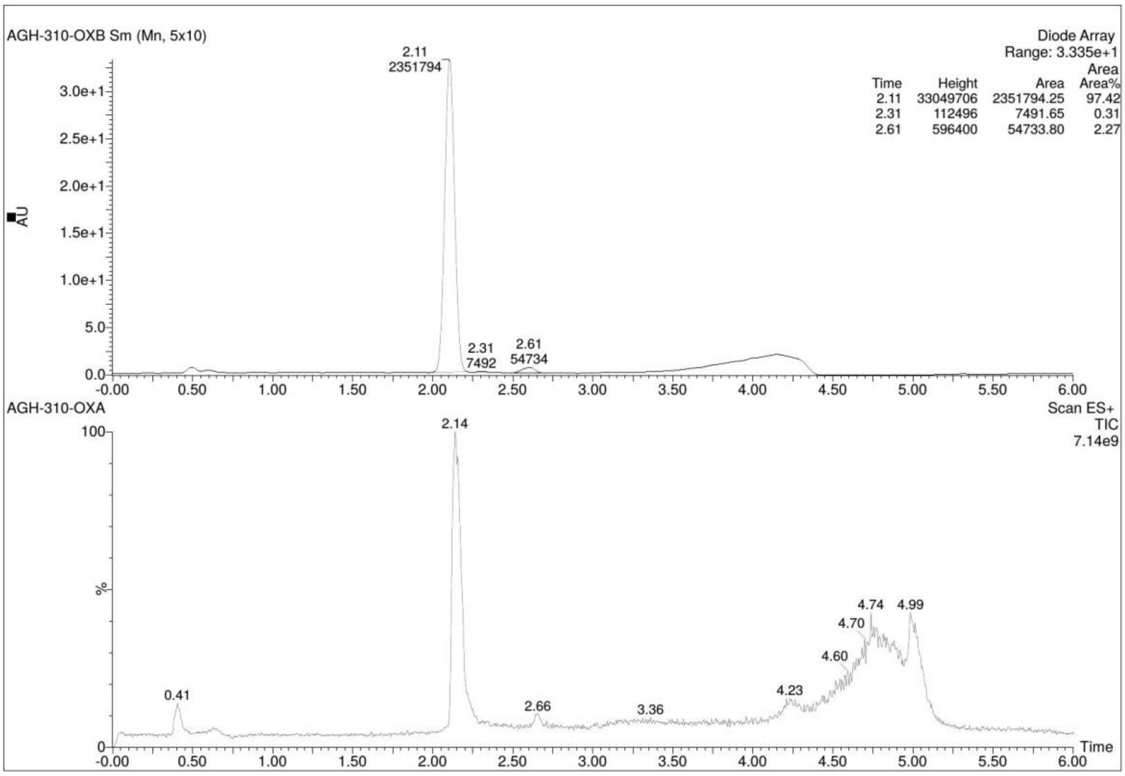

AGH-310-OXB Sm (Mn, 5x10)
Diode Array
Range: 3.335e+1
AU
Time Height Area Area%
2.11 33049706 2351794.25 97.42
2.31 112496 7491.65 0.31
2.61 596400 54733.80 2.27
2.11
2351794
2.31
7492
2.61
54734
AGH-310-OXA
Scan ES+
TIC
7.14e9
2.14
0.41
2.66
3.36
4.23
4.60
4.70
4.74
4.99
%
Time

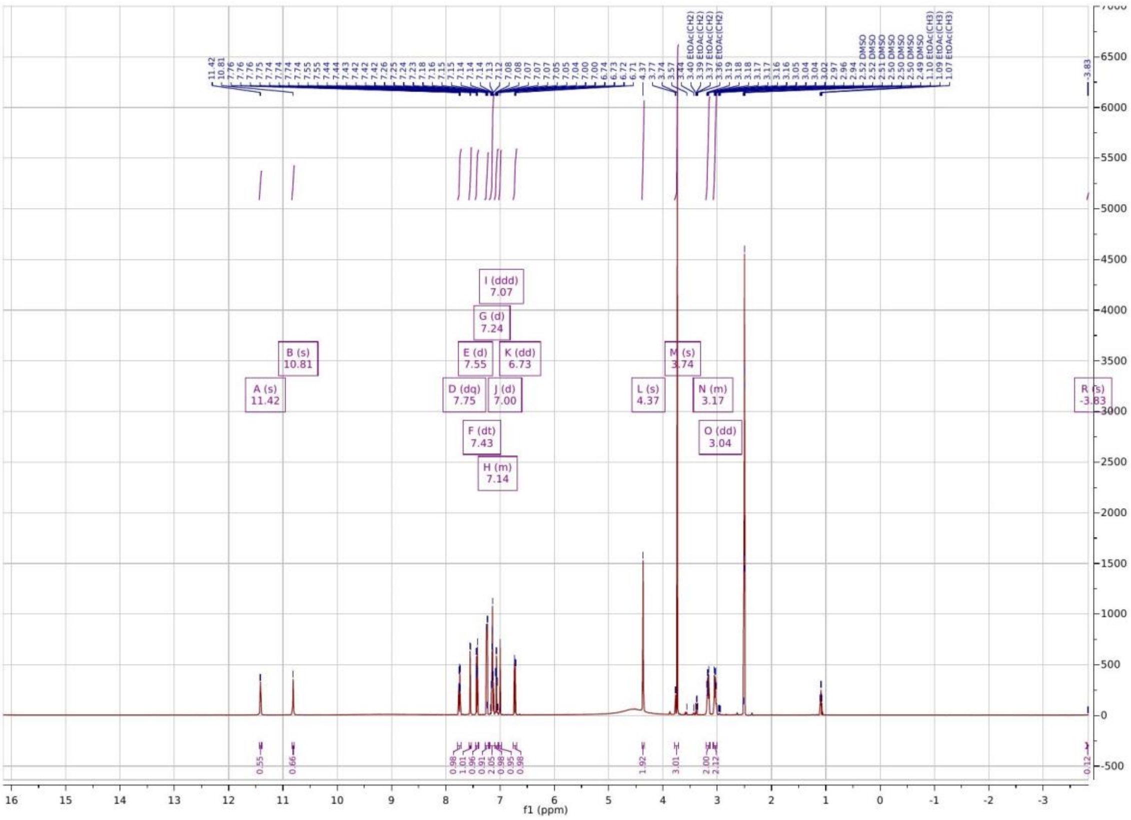

A (s)
11.42
B (s)
10.81
D (dq)
7.75
E (d)
7.55
F (dt)
7.43
G (d)
7.24
H (m)
7.14
I (ddd)
7.07
J (d)
7.00
K (dd)
6.73
L (s)
4.37
M (s)
3.74
N (m)
3.17
O (dd)
3.04
R (s)
-3.83
f1 (ppm)

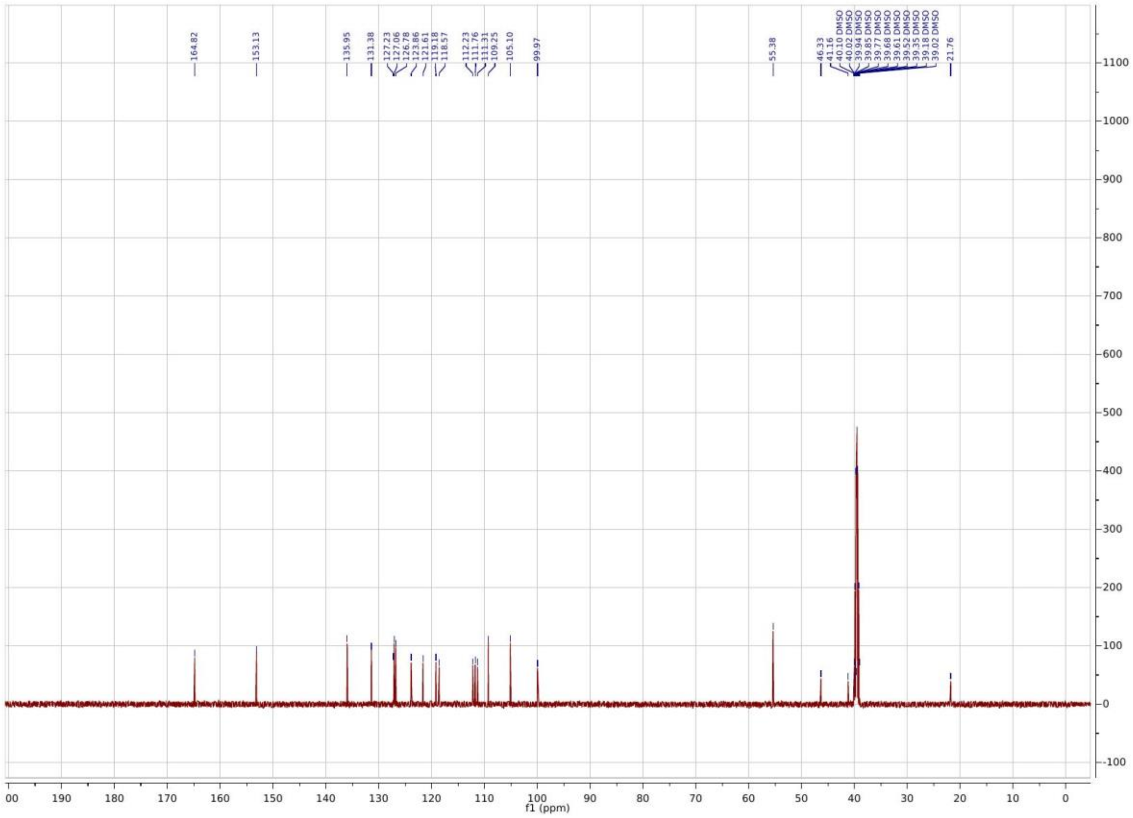

[2-(5-chloro-1*H*-indol-3-yl)ethyl][(1*H*-indol-3-yl)methyl]amine (4)

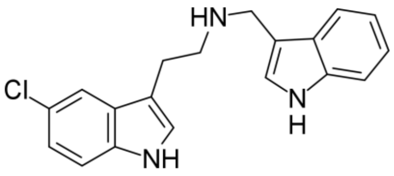

The product was obtained as a white solid. Yield: 68%.
LCMS [M+1] = 323,77 (324,13 calculated).

^{1}H NMR (500 MHz, DMSO-d_6) δ 11.41 (d, *J* = 2.6 Hz, 1H), 11.22 (d, *J* = 2.4 Hz, 1H), 9.16 (s, 2H), 7.79 (dt, *J* = 7.9, 1.0 Hz, 1H), 7.62 (dd, *J* = 13.2, 2.3 Hz, 2H), 7.43 (dt, *J* = 8.1, 1.0 Hz, 1H), 7.41 – 7.34 (m, 1H), 7.30 (d, *J* = 2.4 Hz, 1H), 7.14 (ddd, *J* = 8.2, 7.0, 1.2 Hz, 1H), 7.11 – 7.03 (m, 2H), 4.34 (t, *J* = 5.3 Hz, 2H), 3.18 – 3.06 (m, 4H).
^{13}C NMR (126 MHz, DMSO-d_6) δ 135.88, 134.71, 127.93, 127.37, 126.72, 125.23, 123.23, 121.60, 121.11, 119.15, 118.65, 117.52, 113.08, 111.72, 109.49, 104.98, 46.37, 41.11, 21.47.

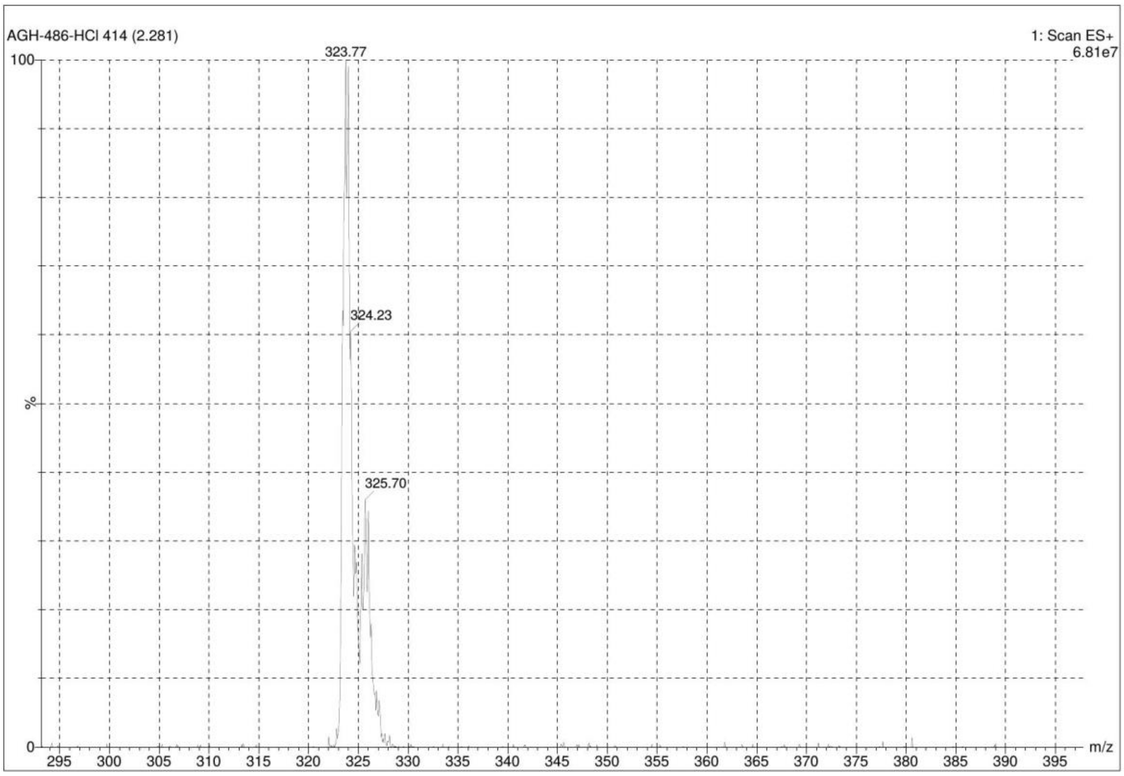

AGH-486-HCl 414 (2.281)
1: Scan ES+
6.81e7
100
%
0
323.77
324.23
325.70
295 300 305 310 315 320 325 330 335 340 345 350 355 360 365 370 375 380 385 390 395
m/z

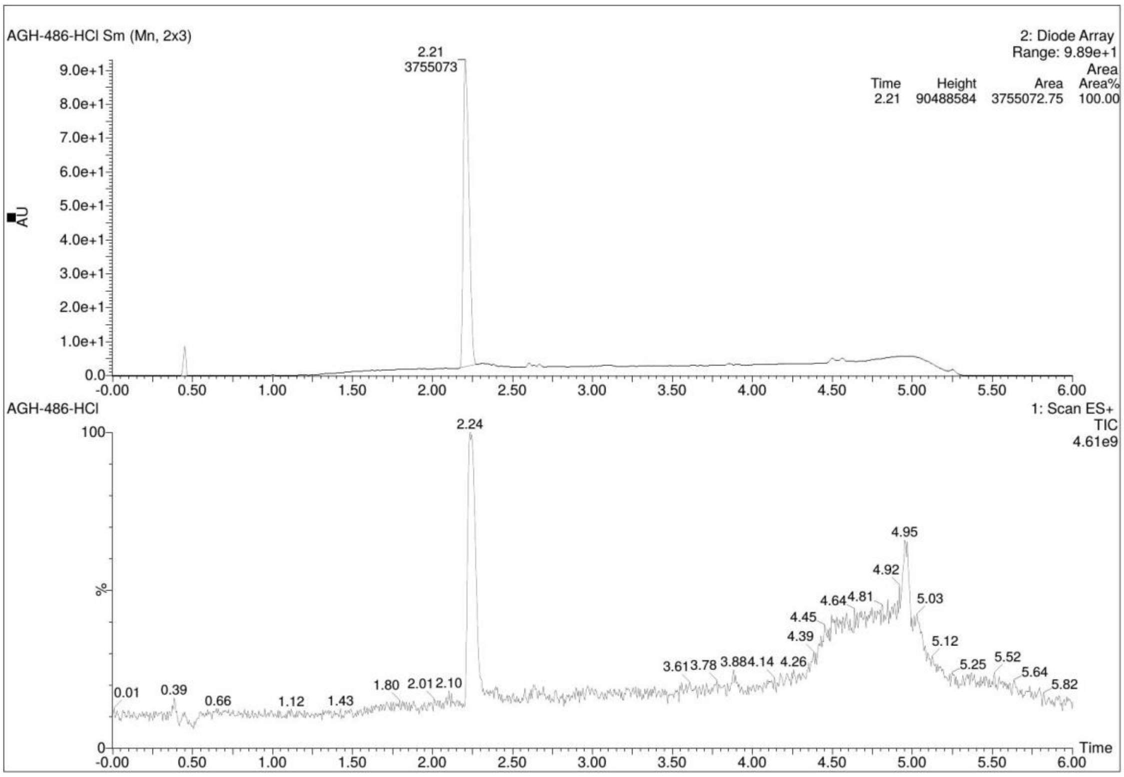

AGH-486-HCl Sm (Mn, 2x3)
2: Diode Array
Range: 9.89e+1
Time Height Area Area%
2.21 90488584 3755072.75 100.00
2.21
3755073
AU
9.0e+1 8.0e+1 7.0e+1 6.0e+1 5.0e+1 4.0e+1 3.0e+1 2.0e+1 1.0e+1 0.0
-0.00 0.50 1.00 1.50 2.00 2.50 3.00 3.50 4.00 4.50 5.00 5.50 6.00
AGH-486-HCl
1: Scan ES+
TIC
4.61e9
100
%
0
2.24
0.01 0.39 0.66 1.12 1.43 1.80 2.01 2.10
3.61 3.78 3.88 4.14 4.26 4.39 4.45 4.64 4.81 4.92 4.95 5.03 5.12 5.25 5.52 5.64 5.82
-0.00 0.50 1.00 1.50 2.00 2.50 3.00 3.50 4.00 4.50 5.00 5.50 6.00
Time

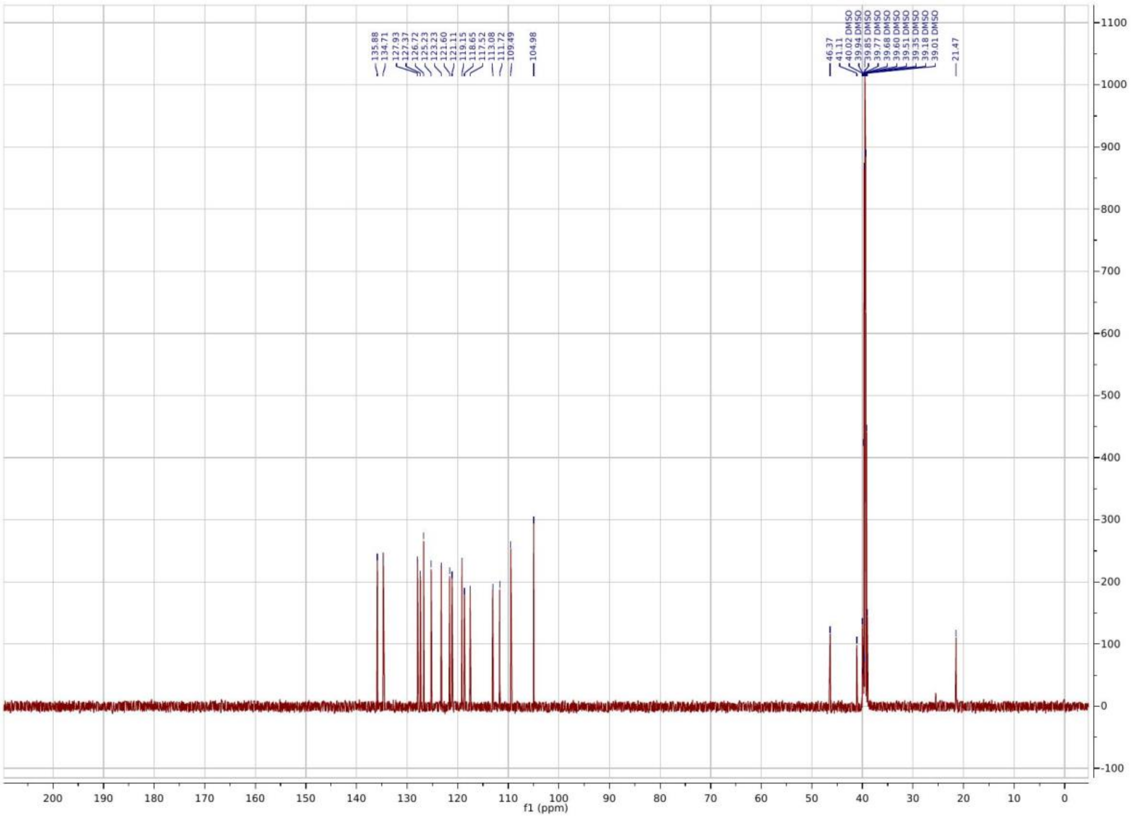

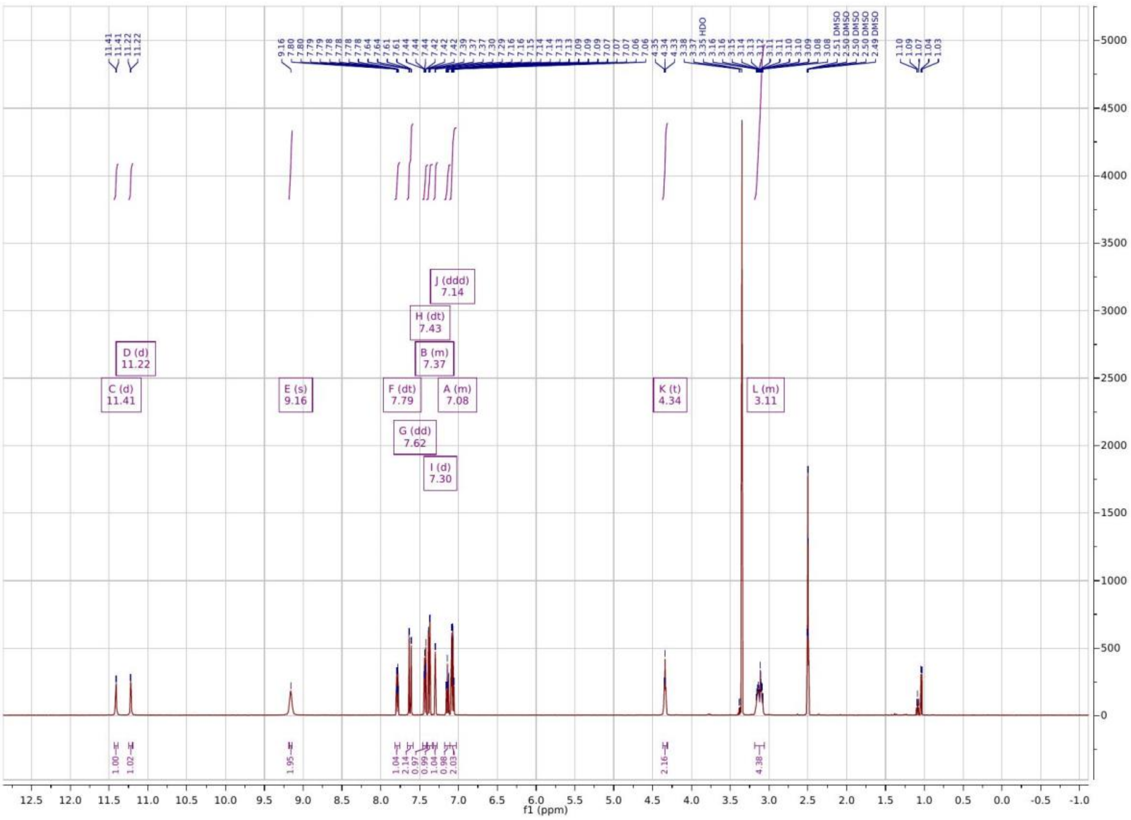

[2-(6-chloro-1*H*-indol-3-yl)ethyl][(1*H*-indol-3-yl)methyl]amine (5)

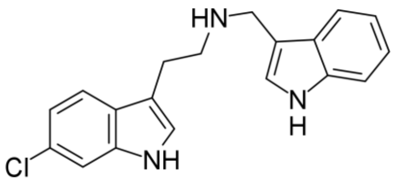

The product was obtained as a beige solid. Yield: 44%.
LCMS [M+1] = 324,43 (324,13 calculated).

^{1}H NMR (500 MHz, DMSO-d_6) δ 11.41 (s, 0H), 11.18 (s, 1H), 7.74 (dt, *J* = 7.8, 0.9 Hz, 1H), 7.59 – 7.49 (m, 2H), 7.46 – 7.39 (m, 2H), 7.25 (d, *J* = 2.3 Hz, 1H), 7.14 (ddd, *J* = 8.2, 7.0, 1.2 Hz, 1H), 7.07 (ddd, *J* = 8.0, 7.0, 1.1 Hz, 1H), 7.00 (dd, *J* = 8.5, 1.9 Hz, 1H), 4.36 (s, 2H), 3.21 – 3.14 (m, 2H), 3.06 (dd, *J* = 9.6, 6.5 Hz, 2H).
^{13}C NMR (126 MHz, DMSO-d_6) δ 164.83, 136.63, 135.93, 127.21, 126.76, 125.92, 125.58, 124.47, 121.61, 119.53, 119.17, 118.77, 118.56, 111.75, 111.17, 109.92, 105.10, 46.35, 41.21, 21.55.

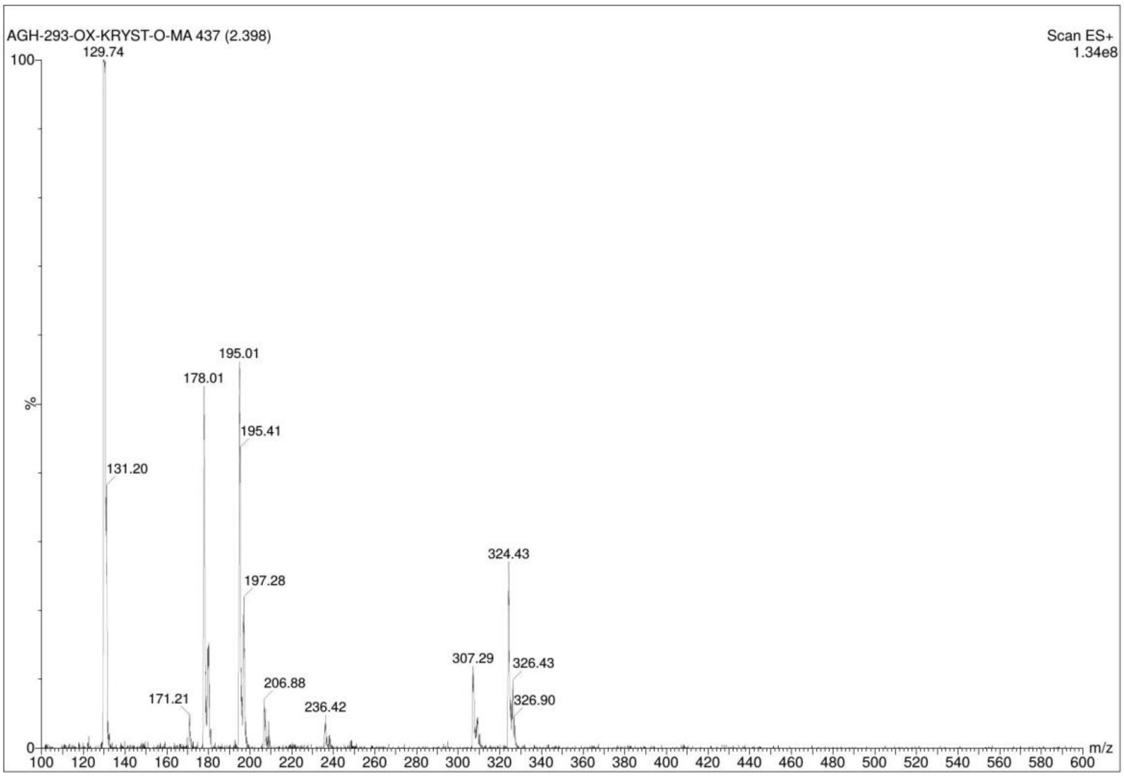

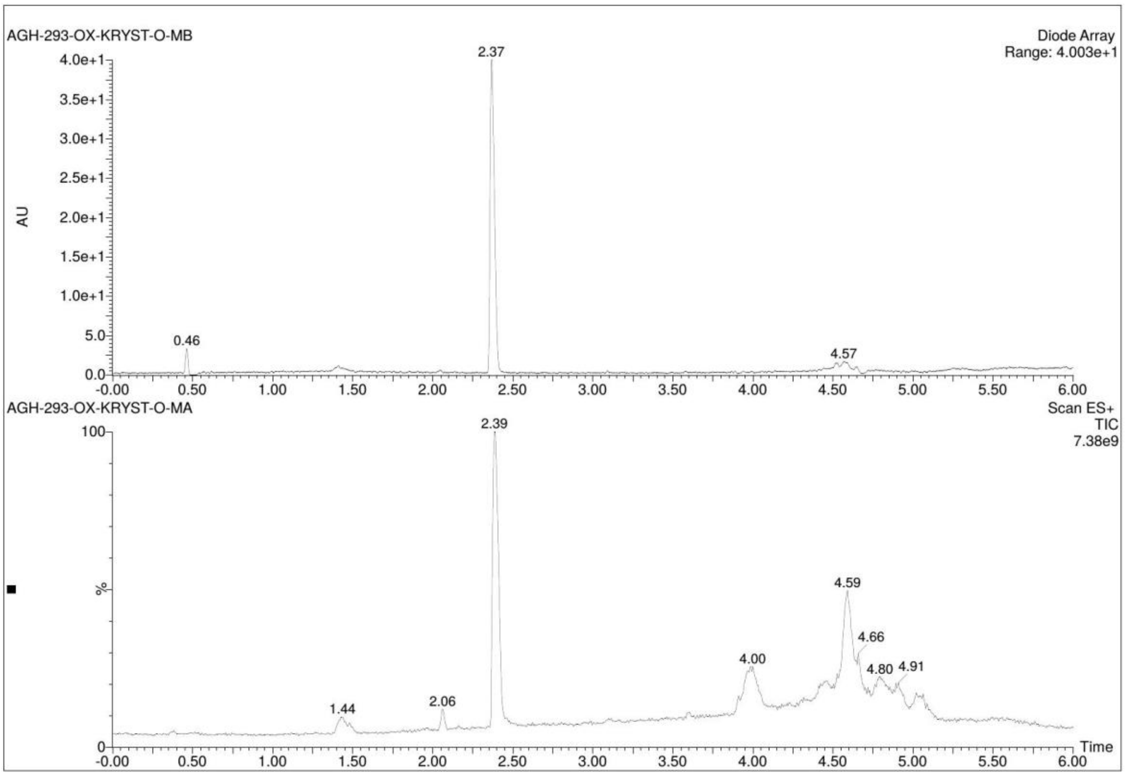
AGH-293-OX-KRYST-O-MB
Diode Array
Range: 4.003e+1
2.37
0.46
4.57
AU
AGH-293-OX-KRYST-O-MA
Scan ES+
TIC
7.38e9
2.39
4.59
4.66
4.00
4.80
4.91
1.44
2.06
%
Time

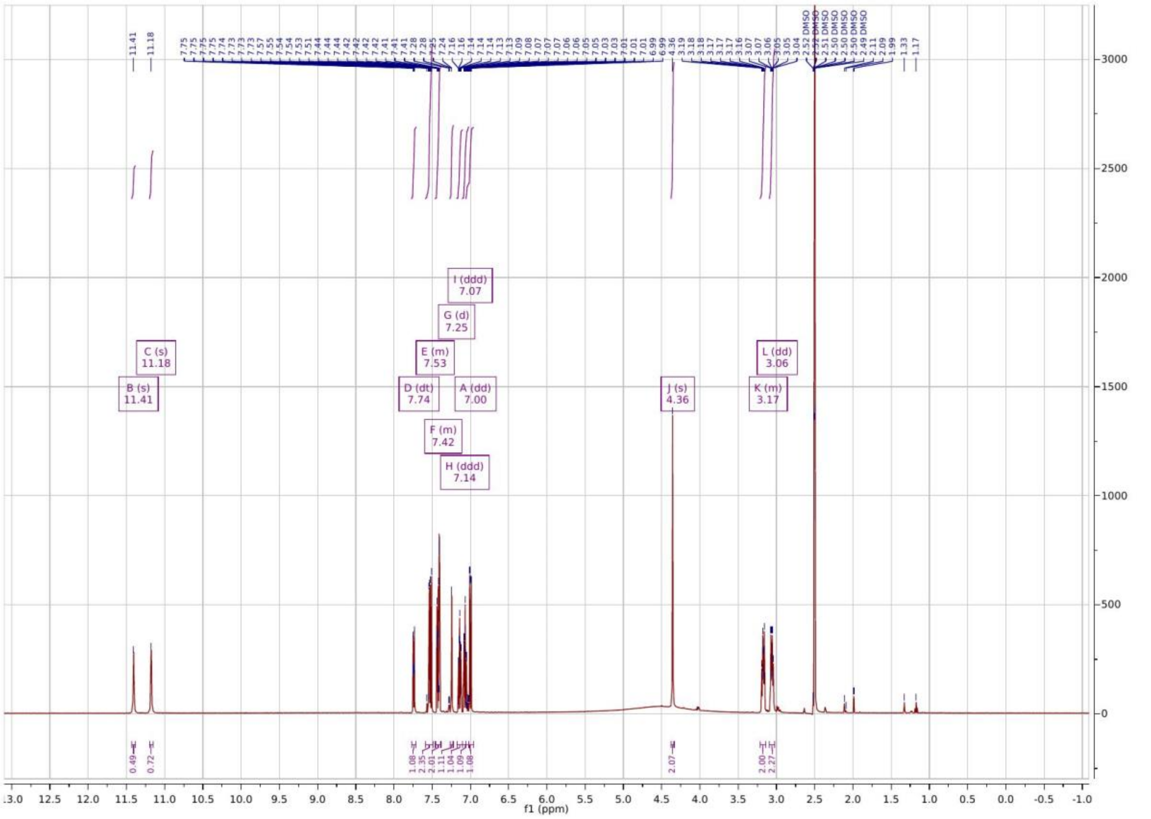
I (ddd)
7.07
G (d)
7.25
C (s)
11.18
E (m)
7.53
L (dd)
3.06
B (s)
11.41
D (dt)
7.74
A (dd)
7.00
J (s)
4.36
K (m)
3.17
F (m)
7.42
H (ddd)
7.14
f1 (ppm)

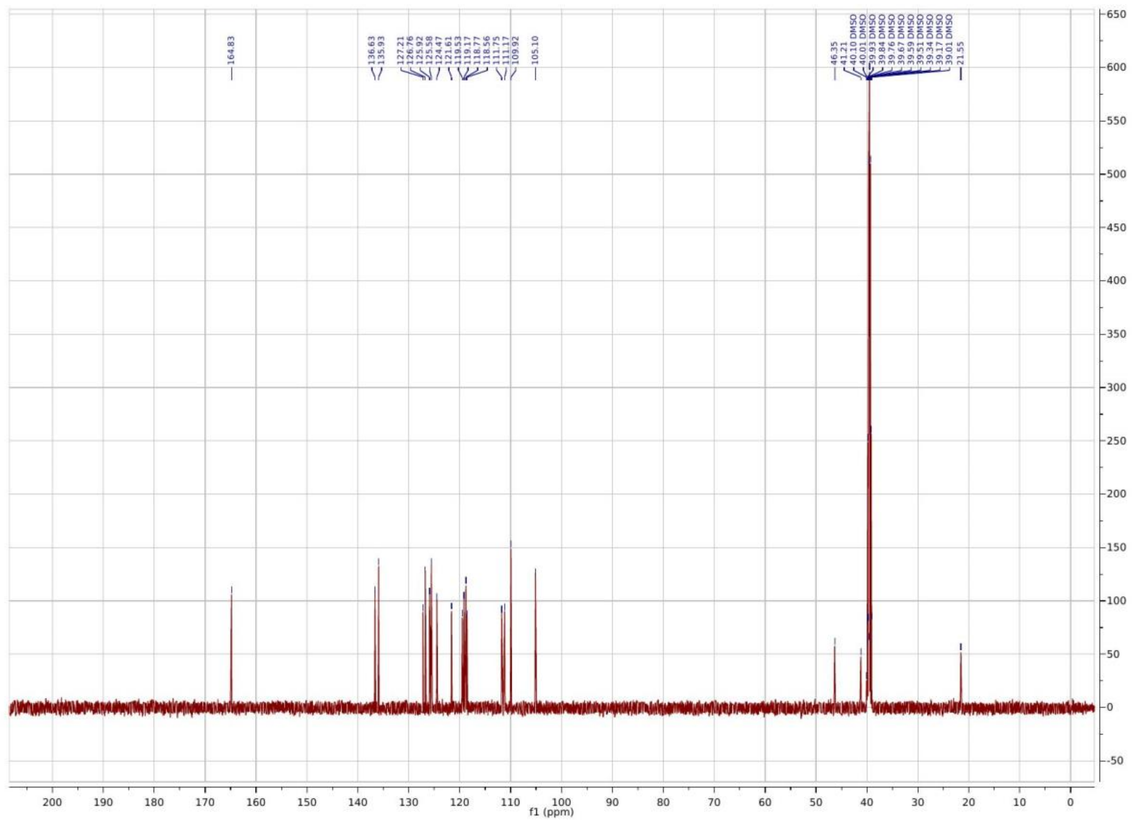

[2-(7-fluoro-1*H*-indol-3-yl)ethyl][(1*H*-indol-3-yl)methyl]amine (6)

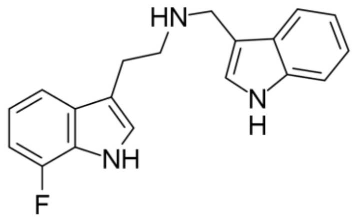

The product was obtained as a yellow solid. Yield: 90%.
LCMS [M+1] = 308,09 (308,16 calculated).

^{1}H NMR (500 MHz, DMSO-d_6) δ 11.48 (s, 1H), 11.45 (s, 1H), 9.31 (s, 2H), 7.79 (dd, *J* = 8.0, 1.0 Hz, 1H), 7.63 (d, *J* = 2.6 Hz, 1H), 7.43 (dd, *J* = 8.1, 1.0 Hz, 1H), 7.41 – 7.36 (m, 1H), 7.28 (d, *J* = 2.5 Hz, 1H), 7.14 (ddd, *J* = 8.2, 7.0, 1.2 Hz, 1H), 7.06 (ddd, *J* = 8.0, 7.0, 1.0 Hz, 1H), 7.00 – 6.86 (m, 2H), 4.34 (s, 2H), 3.15 (t, *J* = 3.1 Hz, 4H).
^{13}C NMR (126 MHz, DMSO-d_6) δ 150.24, 148.31, 135.89, 130.87, 130.82, 127.43, 126.75, 124.47, 124.03, 123.92, 121.57, 119.14, 118.85, 118.80, 118.67, 114.52, 114.50, 111.71, 110.78, 110.76, 106.08, 105.95, 104.92, 46.24, 41.11, 21.59.

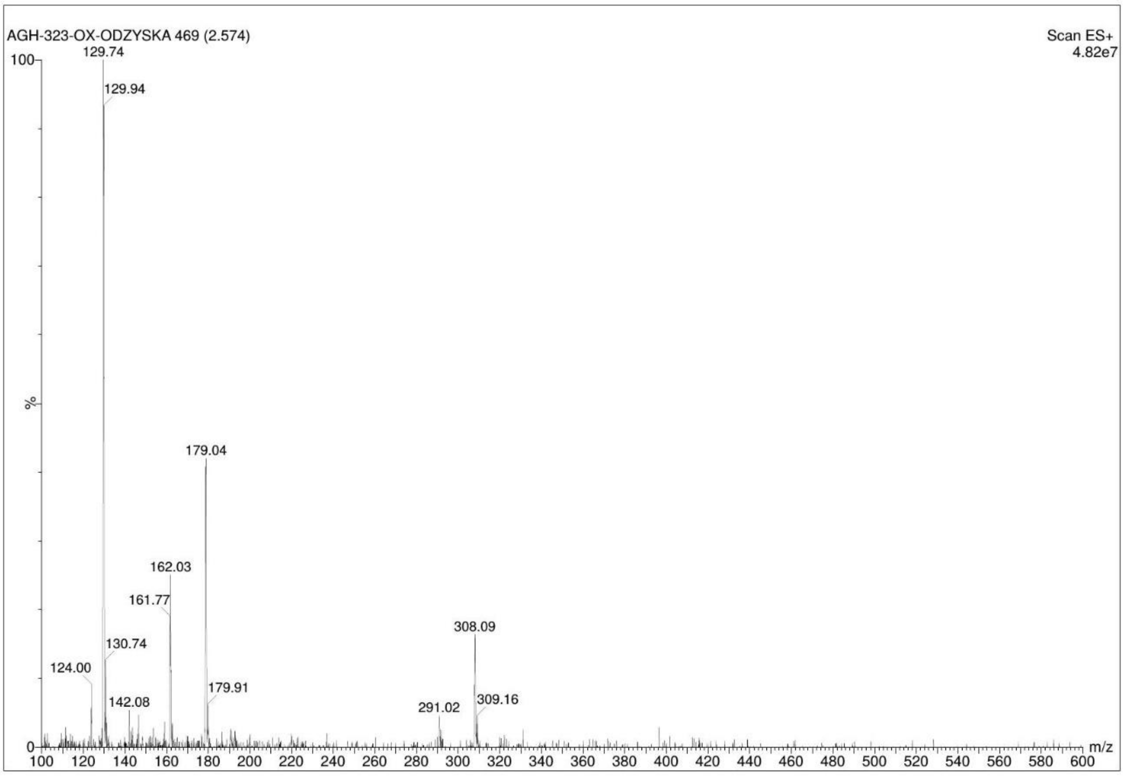
AGH-323-OX-ODZYSKA 469 (2.574)
Scan ES+
4.82e7
129.74
129.94
179.04
162.03
161.77
308.09
130.74
124.00
179.91
309.16
142.08
291.02
%
m/z

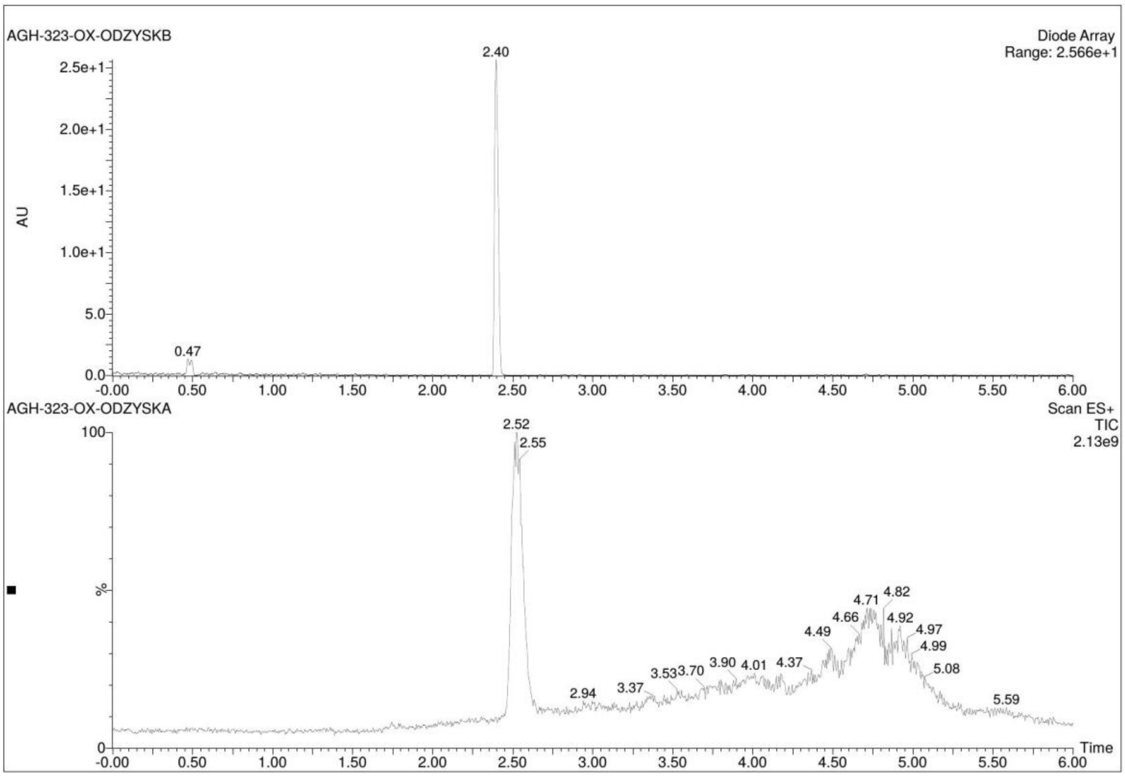
AGH-323-OX-ODZYSKB
Diode Array
Range: 2.566e+1
2.40
0.47
AU
AGH-323-OX-ODZYSKA
Scan ES+
TIC
2.13e9
2.52
2.55
2.94
3.37
3.53
3.70
3.90
4.01
4.37
4.49
4.66
4.71
4.82
4.92
4.97
4.99
5.08
5.59
%
Time

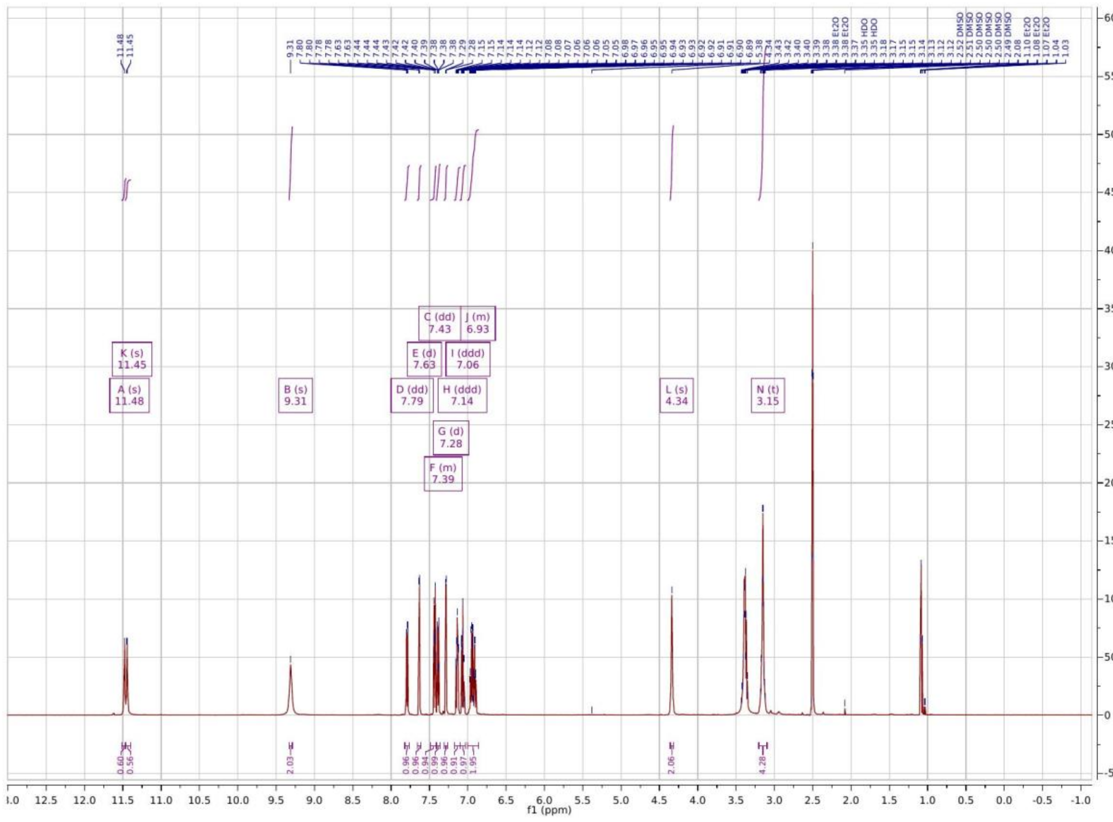

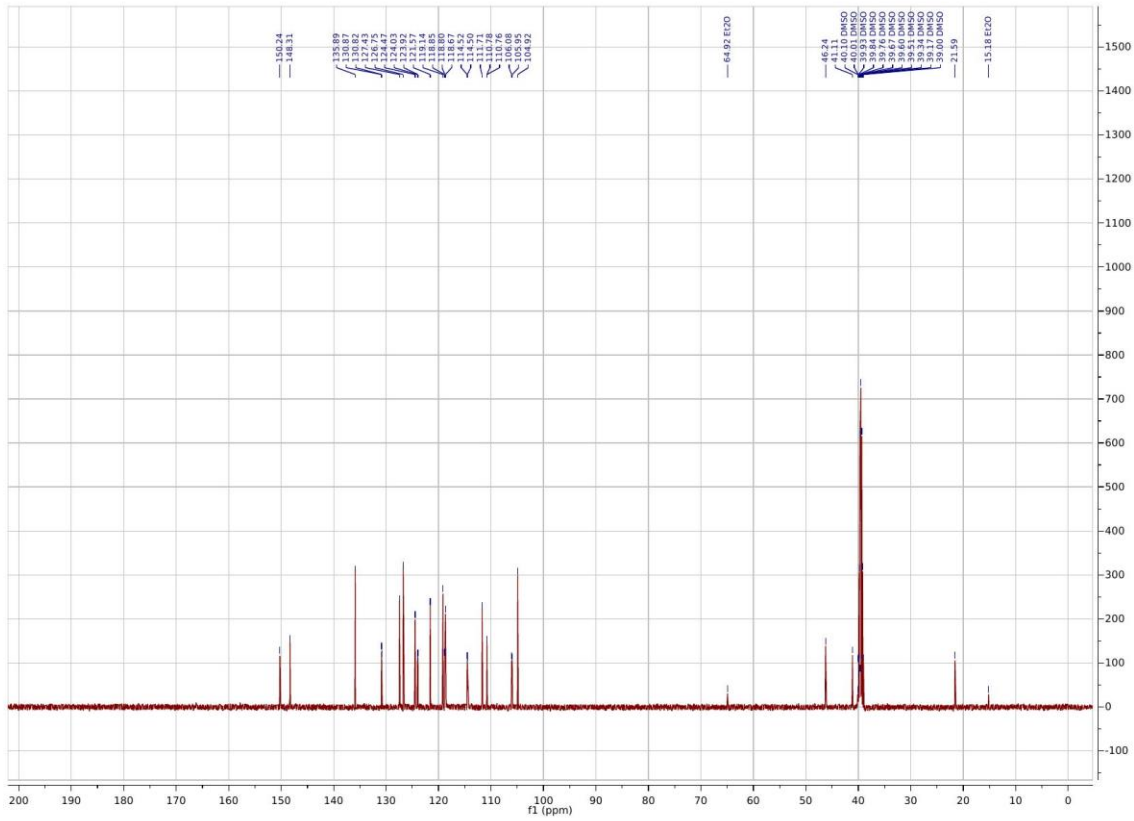

[2-(7-chloro-1*H*-indol-3-yl)ethyl][(1*H*-indol-3-yl)methyl]amine (7)

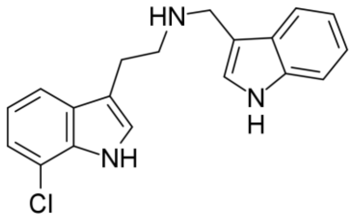

The product was obtained as a light brown solid. Yield: 61%.
LCMS [M+1] = 324,02 (324,13 calculated).

^{1}H NMR (500 MHz, DMSO-d_6) δ 11.47 – 11.38 (m, 1H), 11.38 – 11.29 (m, 1H), 7.75 (d, *J* = 7.9 Hz, 1H), 7.55 (d, *J* = 2.5 Hz, 1H), 7.51 (d, *J* = 7.8 Hz, 1H), 7.43 (dt, *J* = 8.1, 0.9 Hz, 1H), 7.29 (d, *J* = 2.3 Hz, 1H), 7.17 (dd, *J* = 7.5, 0.8 Hz, 1H), 7.14 (ddd, *J* = 8.1, 6.9, 1.1 Hz, 1H), 7.07 (td, *J* = 7.4, 6.9, 1.0 Hz, 1H), 7.00 (t, *J* = 7.7 Hz, 1H), 4.36 (s, 2H), 3.19 (dd, *J* = 9.7, 6.3 Hz, 2H), 3.08 (dd, *J* = 9.6, 6.4 Hz, 2H).
^{13}C NMR (126 MHz, DMSO-d_6) δ 164.83, 135.93, 133.02, 128.78, 127.25, 126.77, 124.71, 121.60, 120.71, 119.56, 119.16, 118.56, 117.31, 115.95, 111.75, 111.02, 105.06, 46.28, 41.20, 21.64.

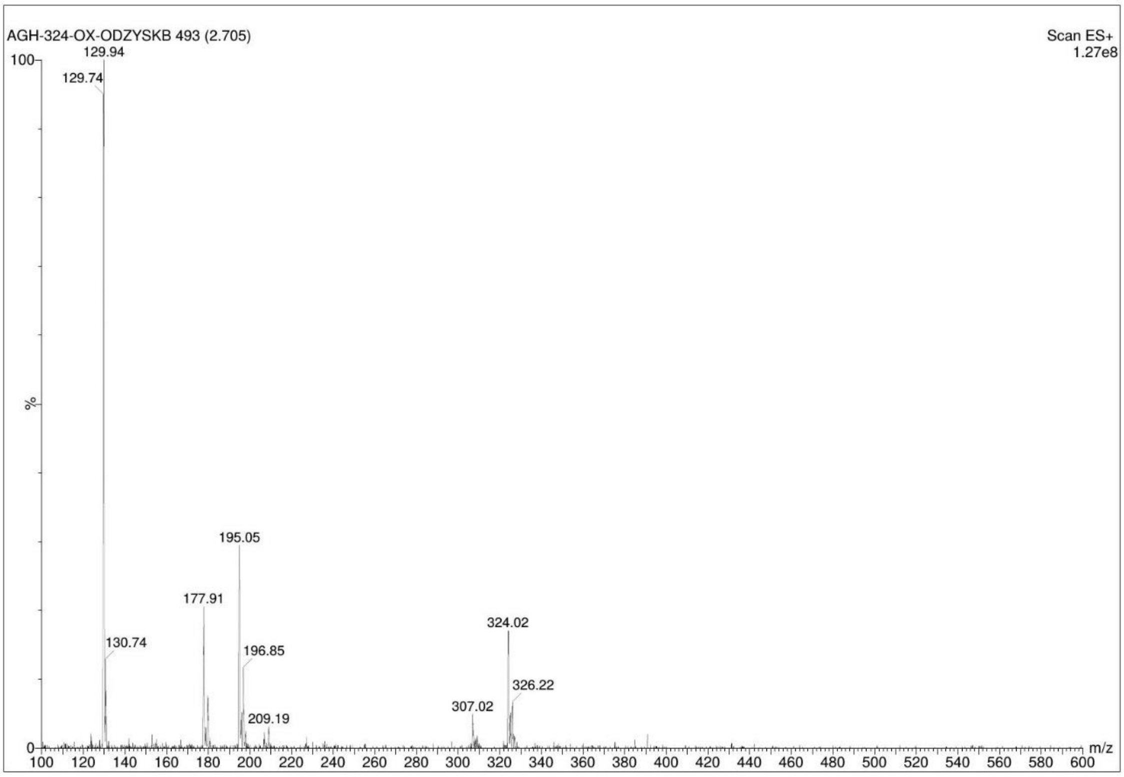

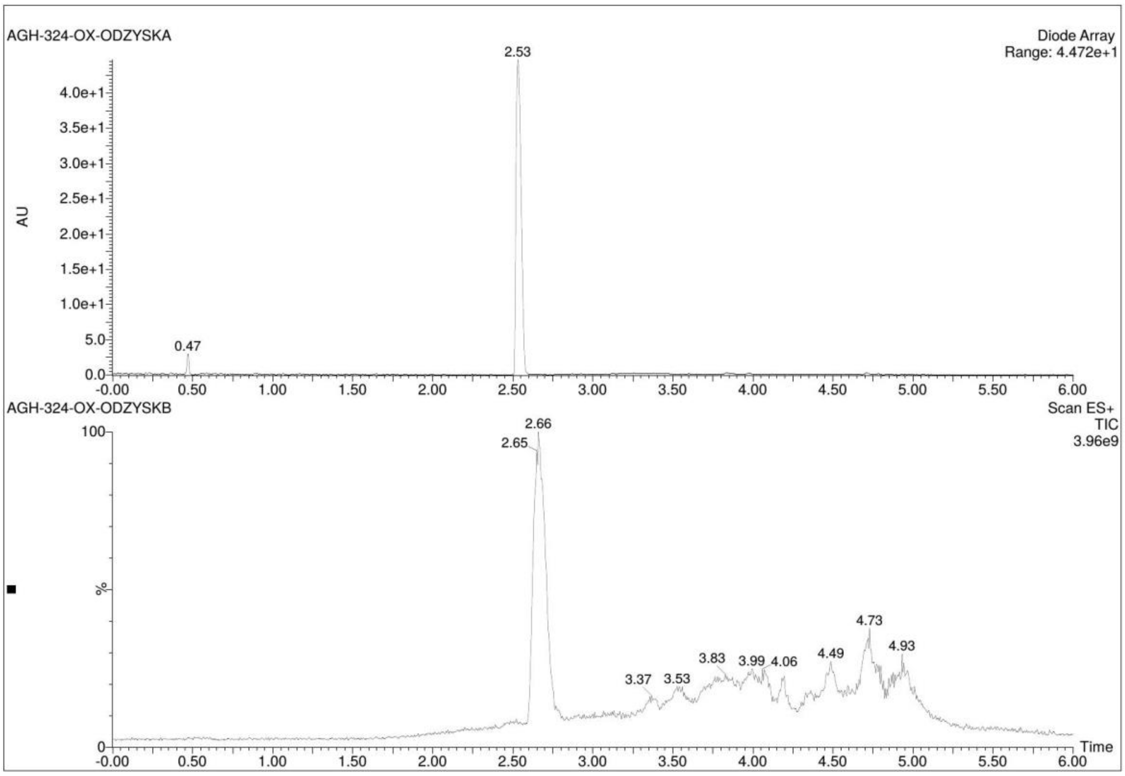
AGH-324-OX-ODZYSKA
Diode Array
Range: 4.472e+1
2.53
0.47
AU
AGH-324-OX-ODZYSKB
Scan ES+
TIC
3.96e9
2.66
2.65
3.37
3.53
3.83
3.99
4.06
4.49
4.73
4.93
%
Time

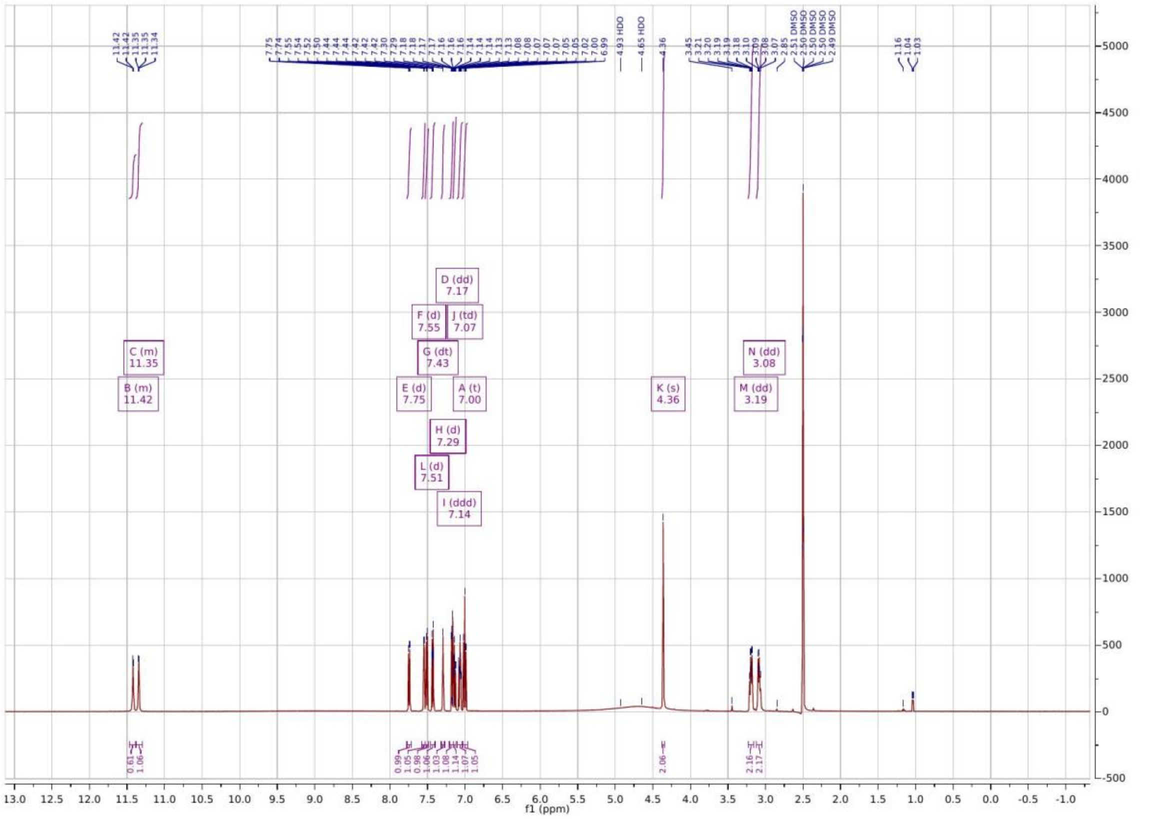
D (dd) 7.17
F (d) 7.55
J (td) 7.07
C (m) 11.35
G (dt) 7.43
N (dd) 3.08
B (m) 11.42
E (d) 7.75
A (t) 7.00
K (s) 4.36
M (dd) 3.19
H (d) 7.29
L (d) 7.51
I (ddd) 7.14
4.93 HDO
4.65 HDO
f1 (ppm)

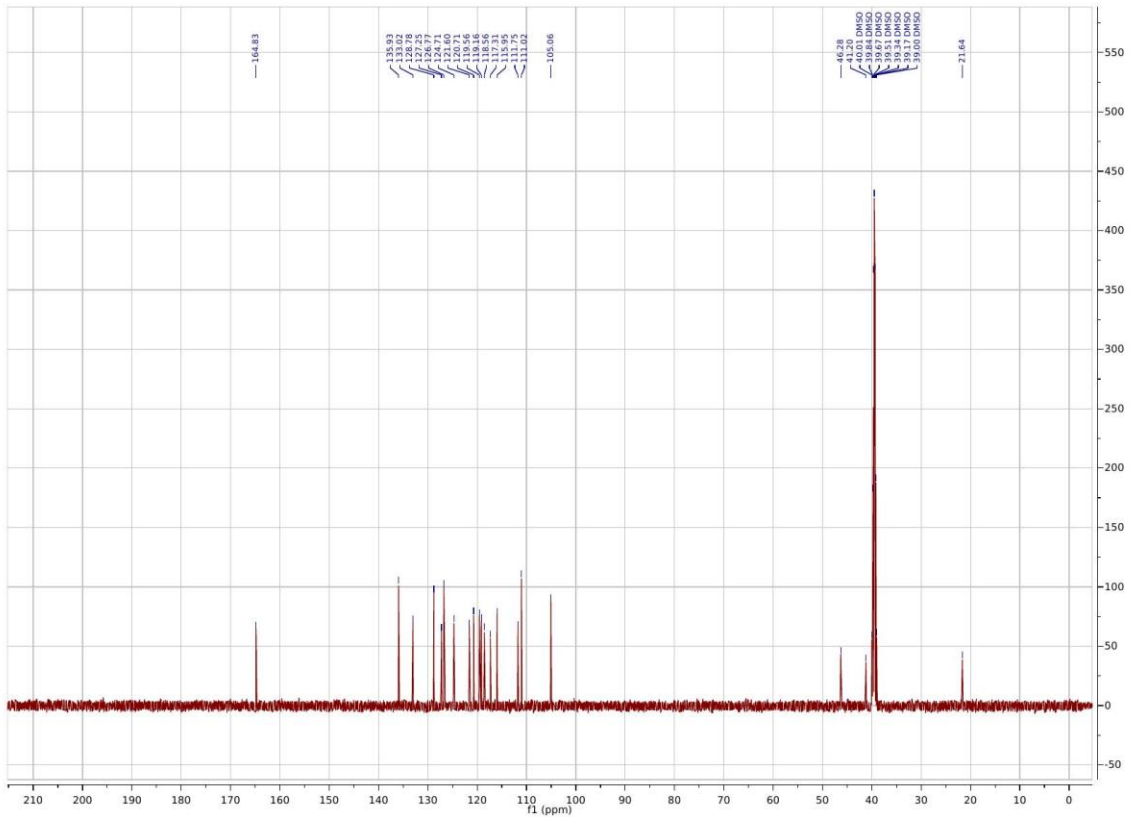

[2-(7-bromo-1*H*-indol-3-yl)ethyl][(1*H*-indol-3-yl)methyl]amine (8)

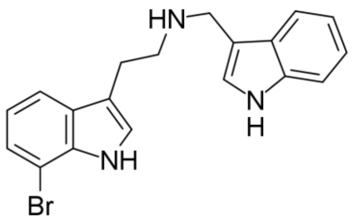

The product was obtained as a white solid. Yield: 43%.
LCMS [M+1] = 369,96 (368,08 calculated).

^{1}H NMR (500 MHz, DMSO-d_6) δ 11.41 (d, *J* = 2.5 Hz, 1H), 11.20 (d, *J* = 2.5 Hz, 1H), 7.77 – 7.71 (m, 1H), 7.61 – 7.52 (m, 2H), 7.43 (dt, *J* = 8.1, 1.0 Hz, 1H), 7.35 – 7.30 (m, 1H), 7.29 (d, *J* = 2.5 Hz, 1H), 7.14 (ddd, *J* = 8.2, 7.0, 1.2 Hz, 1H), 7.07 (ddd, *J* = 8.1, 7.0, 1.1 Hz, 1H), 6.95 (t, *J* = 7.7 Hz, 1H), 4.36 (s, 2H), 3.23 – 3.16 (m, 2H), 3.07 (dd, *J* = 9.3, 6.6 Hz, 2H).
^{13}C NMR (126 MHz, DMSO-d_6) δ 164.79, 135.93, 134.52, 128.54, 127.23, 126.76, 124.71, 123.76, 121.60, 119.98, 119.17, 118.56, 117.79, 111.75, 111.09, 105.07, 104.30, 46.28, 41.22, 25.50, 21.70.

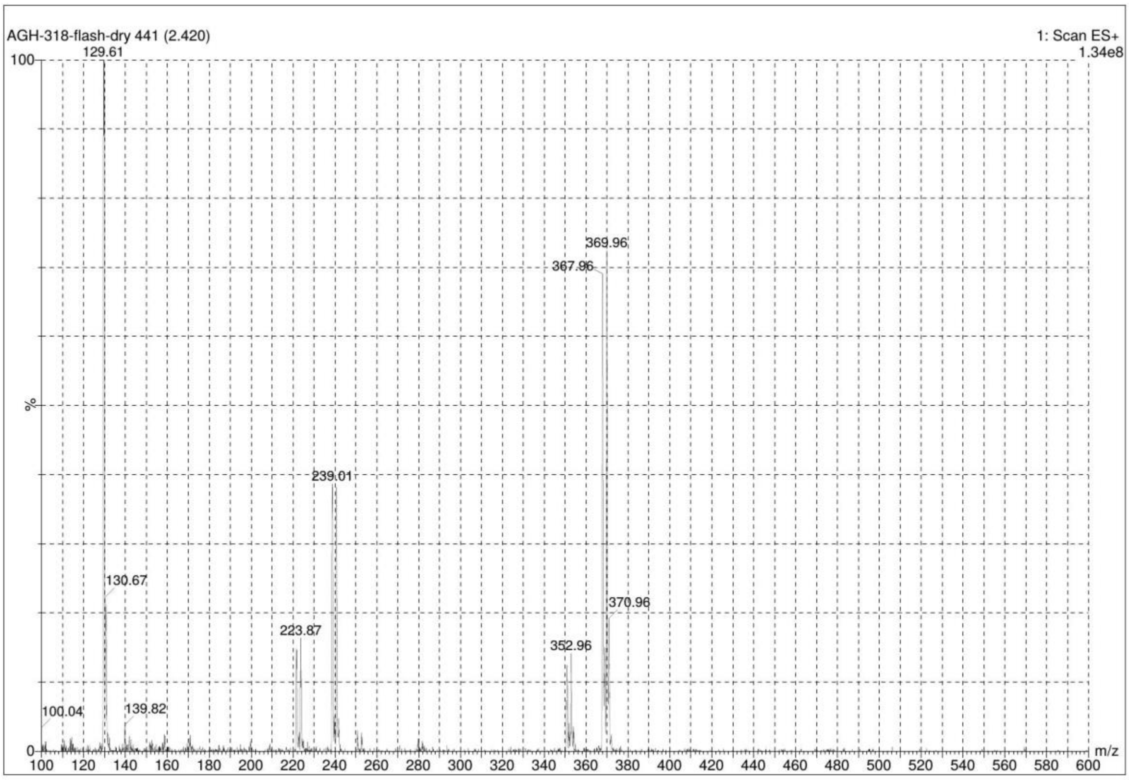
AGH-318-flash-dry 441 (2.420)
1: Scan ES+
1.34e8
100
%
0
129.61
369.96
367.96
239.01
130.67
370.96
223.87
352.96
100.04
139.82
m/z
100 120 140 160 180 200 220 240 260 280 300 320 340 360 380 400 420 440 460 480 500 520 540 560 580 600

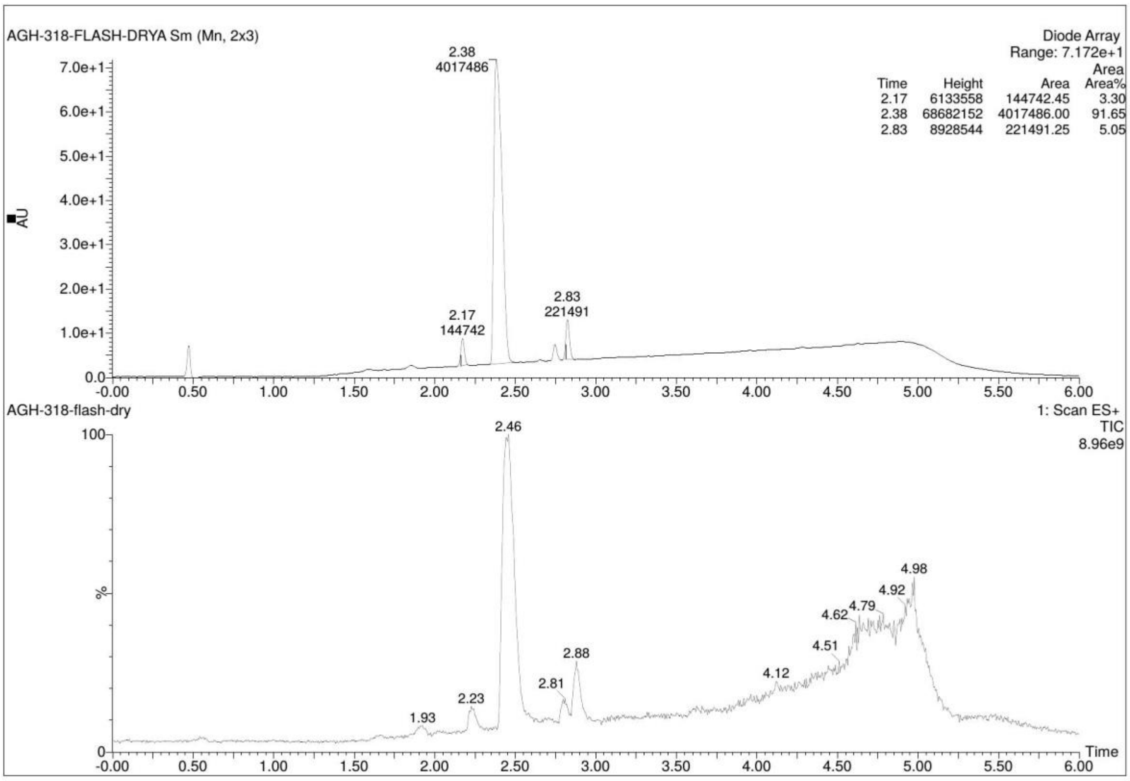
AGH-318-FLASH-DRYA Sm (Mn, 2x3)
Diode Array
Range: 7.172e+1
Time Height Area Area%
2.17 6133558 144742.45 3.30
2.38 68682152 4017486.00 91.65
2.83 8928544 221491.25 5.05
AU
7.0e+1 6.0e+1 5.0e+1 4.0e+1 3.0e+1 2.0e+1 1.0e+1 0.0
2.38
4017486
2.17
144742
2.83
221491
-0.00 0.50 1.00 1.50 2.00 2.50 3.00 3.50 4.00 4.50 5.00 5.50 6.00
AGH-318-flash-dry
1: Scan ES+
TIC
8.96e9
100
%
0
2.46
1.93
2.23
2.81
2.88
4.12
4.51
4.62
4.79
4.92
4.98
Time
-0.00 0.50 1.00 1.50 2.00 2.50 3.00 3.50 4.00 4.50 5.00 5.50 6.00

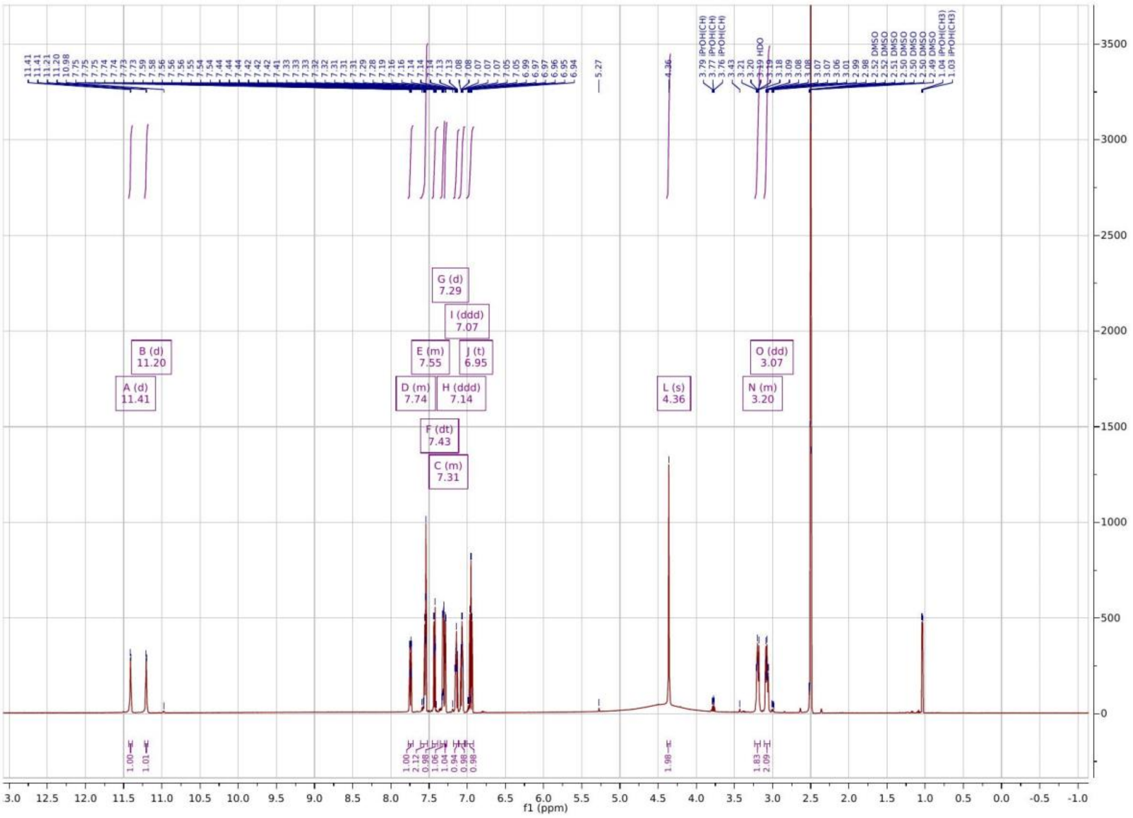

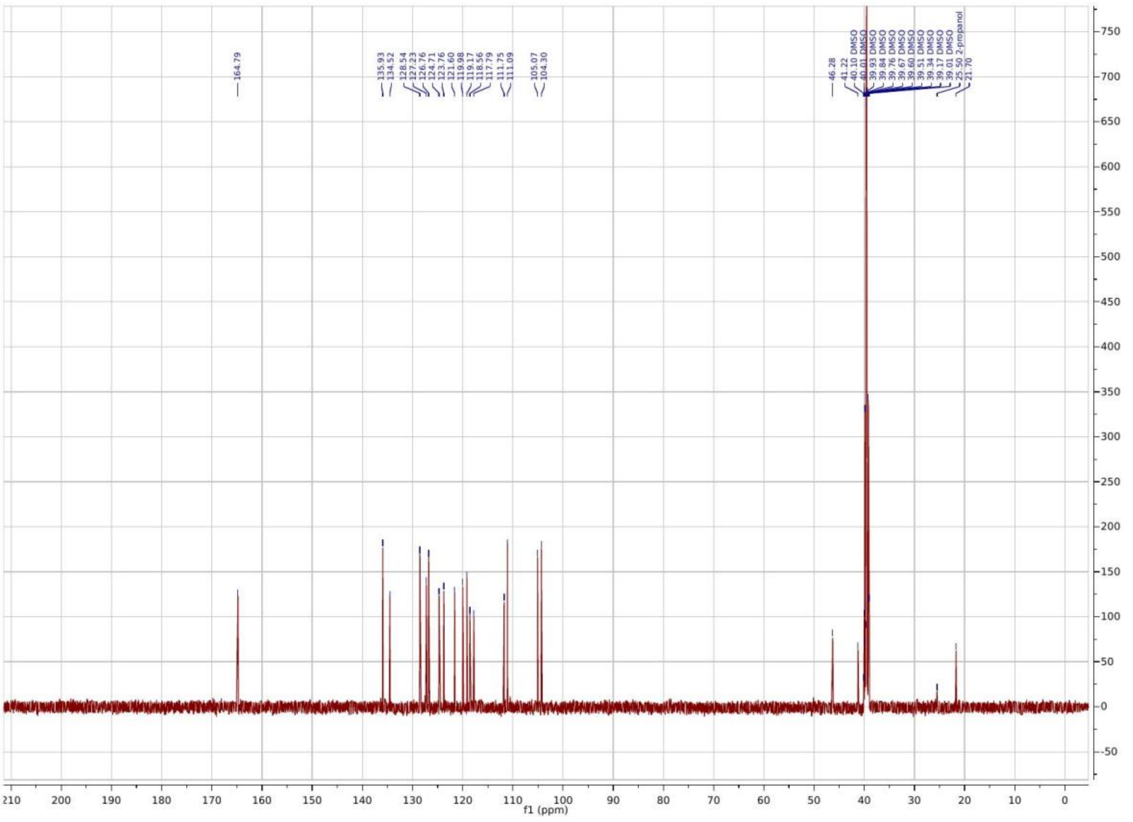

[2-(1*H*-indol-3-yl)ethyl][(5-methyl-1*H*-indol-3-yl)methyl]amine (9)

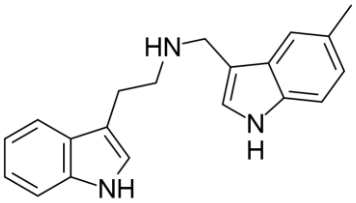

The product was obtained as a beige solid. Yield: 40%.
LCMS [M+1] = 303,93 (304,18 calculated).

^{1}H NMR (500 MHz, DMSO-d_6) δ 11.27 (d, J = 2.4 Hz, 1H), 10.98 (d, J = 2.4 Hz, 1H), 7.52 (d, J = 8.1 Hz, 2H), 7.48 (d, J = 2.6 Hz, 1H), 7.36 (dd, J = 8.1, 1.0 Hz, 1H), 7.37 – 7.28 (m, 1H), 7.20 (d, J = 2.4 Hz, 1H), 7.08 (ddd, J = 8.1, 7.0, 1.2 Hz, 1H), 7.05 – 6.94 (m, 2H), 4.33 (s, 2H), 3.18 (dd, J = 10.5, 5.7 Hz, 2H), 3.11 – 2.99 (m, 4H), 2.39 (s, 3H).
^{13}C NMR (126 MHz, DMSO-d_6) δ 164.75, 136.25, 134.32, 127.69, 127.24, 126.98, 126.74, 123.22, 123.20, 121.15, 118.44, 118.12, 118.09, 111.55, 111.46, 109.52, 104.54, 41.24, 21.70, 21.28.

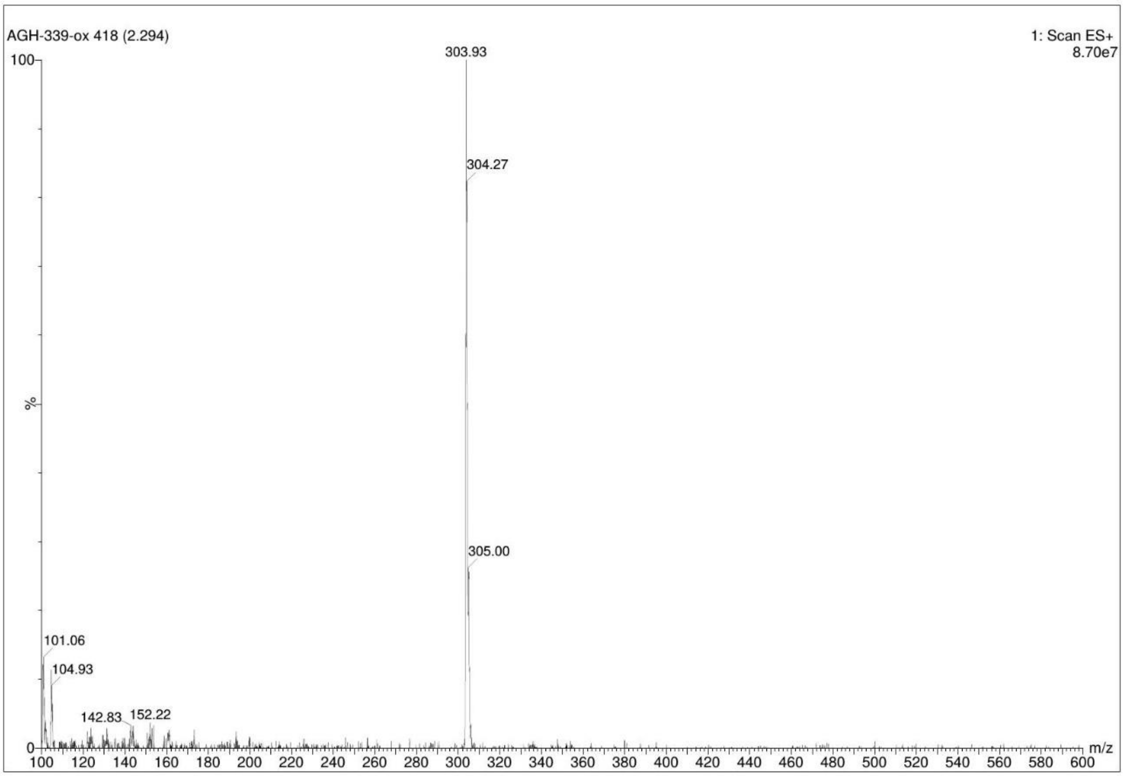

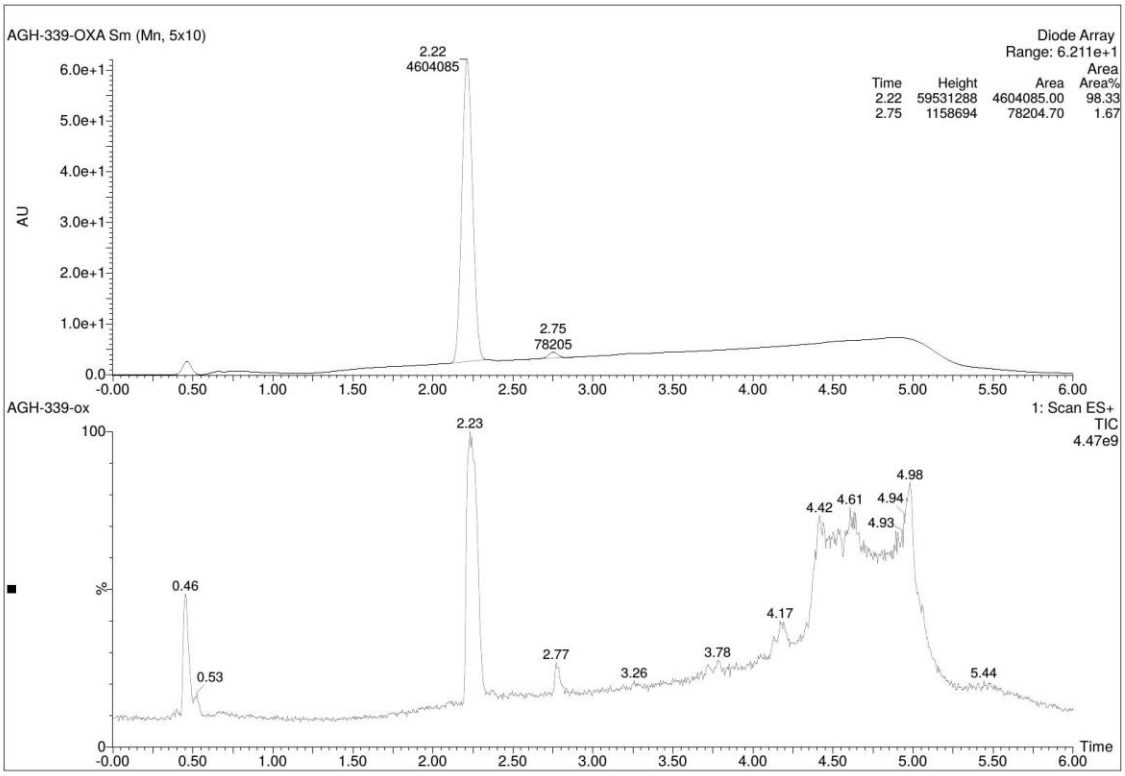
AGH-339-OXA Sm (Mn, 5x10)
Diode Array
Range: 6.211e+1
Time Height Area Area%
2.22 59531288 4604085.00 98.33
2.75 1158694 78204.70 1.67
2.22
4604085
2.75
78205
AU
AGH-339-ox
1: Scan ES+
TIC
4.47e9
2.23
0.46
0.53
2.77
3.26
3.78
4.17
4.42
4.61
4.94
4.93
4.98
5.44
%
Time

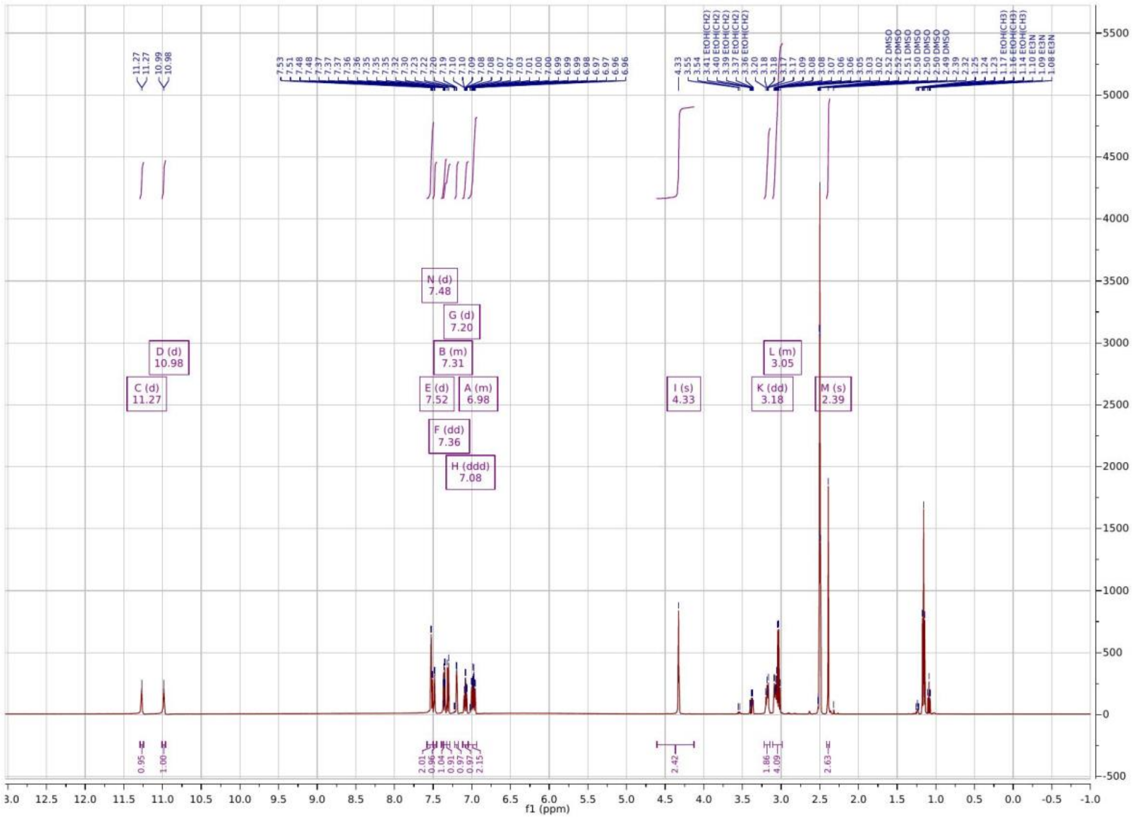
N (d)
7.48
G (d)
7.20
D (d)
10.98
B (m)
7.31
L (m)
3.05
C (d)
11.27
E (d)
7.52
A (m)
6.98
I (s)
4.33
K (dd)
3.18
M (s)
2.39
F (dd)
7.36
H (ddd)
7.08
0.95
1.00
2.01
0.96
1.04
0.91
0.97
0.97
2.15
2.42
1.86
4.09
2.63
f1 (ppm)

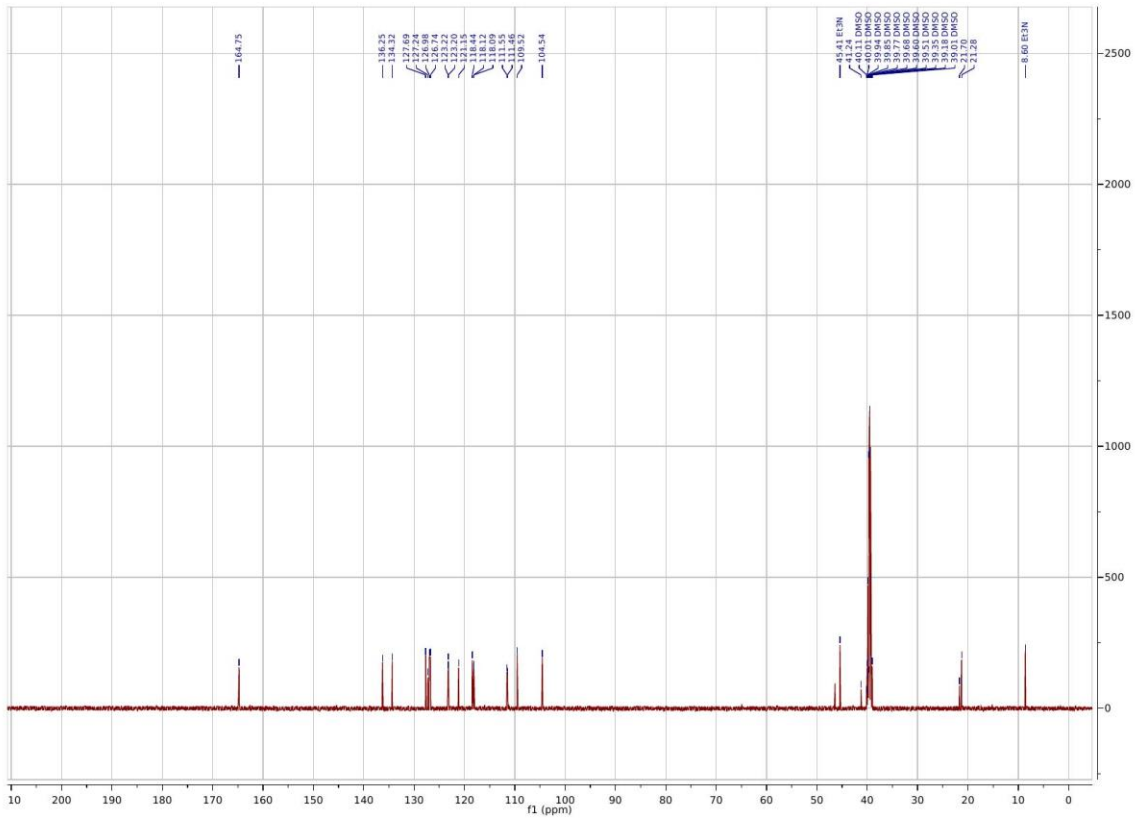

[(5-fluoro-1*H*-indol-3-yl)methyl][2-(1*H*-indol-3-yl)ethyl]amine (10)

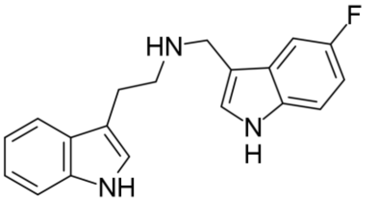

The product was obtained as a light yellow solid. Yield: 66%.
LCMS [M+1] = 306,13 (308,16 calculated).

^{1}H NMR (500 MHz, DMSO-d_6) δ 10.97 – 10.92 (m, 1H), 10.77 (s, 1H), 7.49 (dq, *J* = 8.0, 0.9 Hz, 1H), 7.37 – 7.26 (m, 5H), 7.11 (d, *J* = 2.3 Hz, 1H), 7.04 (ddd, *J* = 8.1, 7.0, 1.2 Hz, 1H), 6.94 (ddd, *J* = 8.0, 7.0, 1.1 Hz, 1H), 6.92 – 6.87 (m, 1H), 3.87 (d, *J* = 0.8 Hz, 2H), 2.86 (s, 4H).
^{13}C NMR (126 MHz, DMSO-d_6) δ 157.49, 155.65, 136.23, 136.17, 133.19, 132.99, 127.69, 127.29, 127.19, 127.16, 125.45, 122.50, 122.19, 120.80, 120.68, 118.33, 118.08, 114.13, 114.09, 112.75, 112.70, 112.60, 112.21, 112.13, 111.32, 109.15, 109.07, 108.86, 103.61, 103.42, 49.65, 44.20, 25.40.

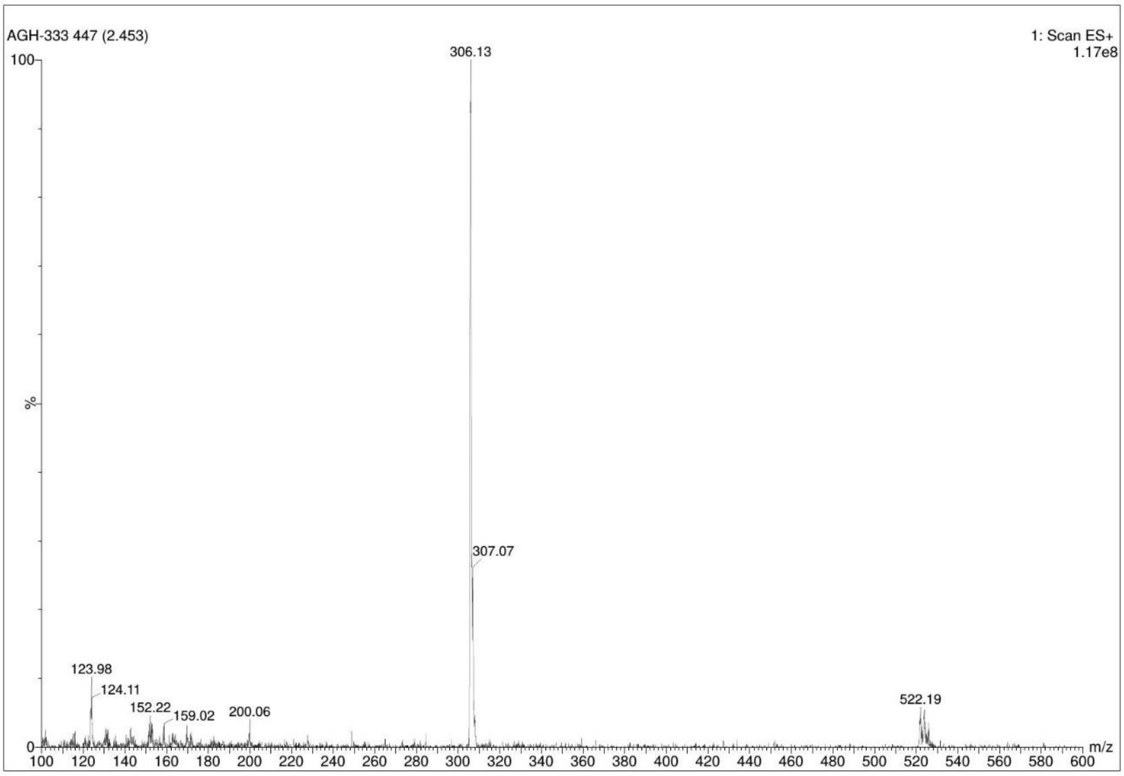

AGH-333 447 (2.453)
1: Scan ES+
1.17e8
306.13
307.07
123.98
124.11
152.22
159.02
200.06
522.19
%
m/z

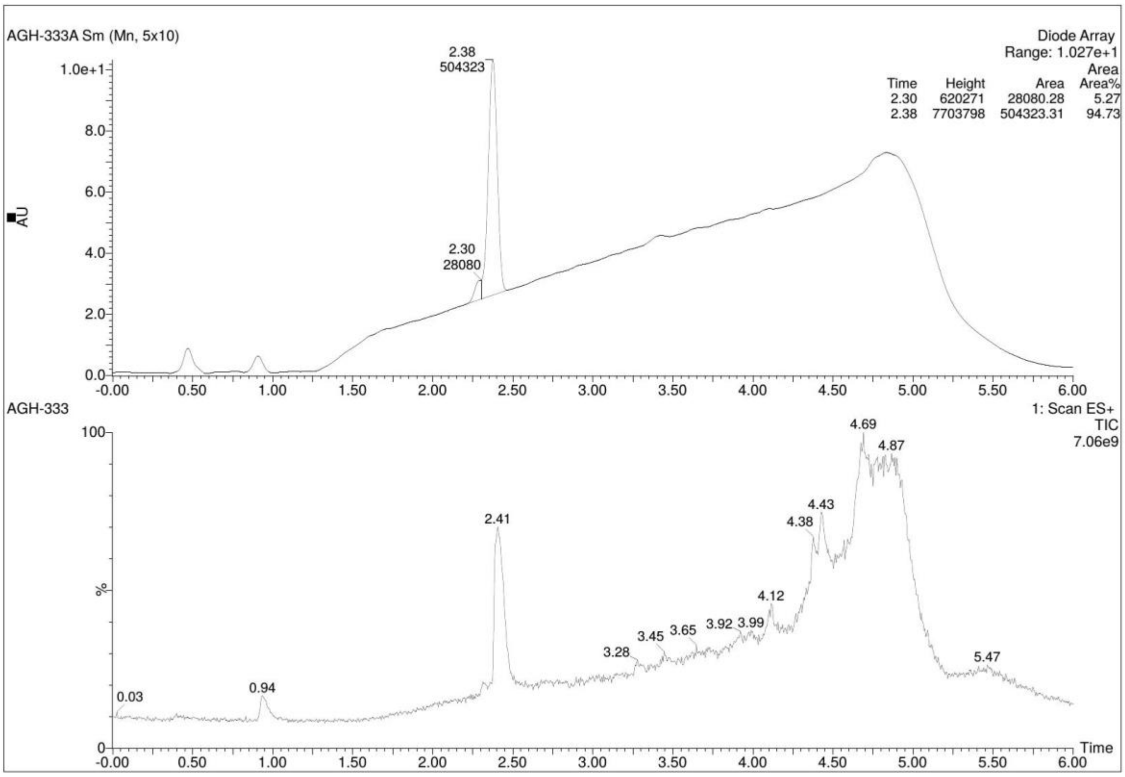

AGH-333A Sm (Mn, 5x10)
Diode Array
Range: 1.027e+1
Time Height Area Area%
2.30 620271 28080.28 5.27
2.38 7703798 504323.31 94.73
2.38
504323
2.30
28080
AU
AGH-333
1: Scan ES+
TIC
7.06e9
0.03
0.94
2.41
3.28
3.45
3.65
3.92
3.99
4.12
4.38
4.43
4.69
4.87
5.47
%
Time

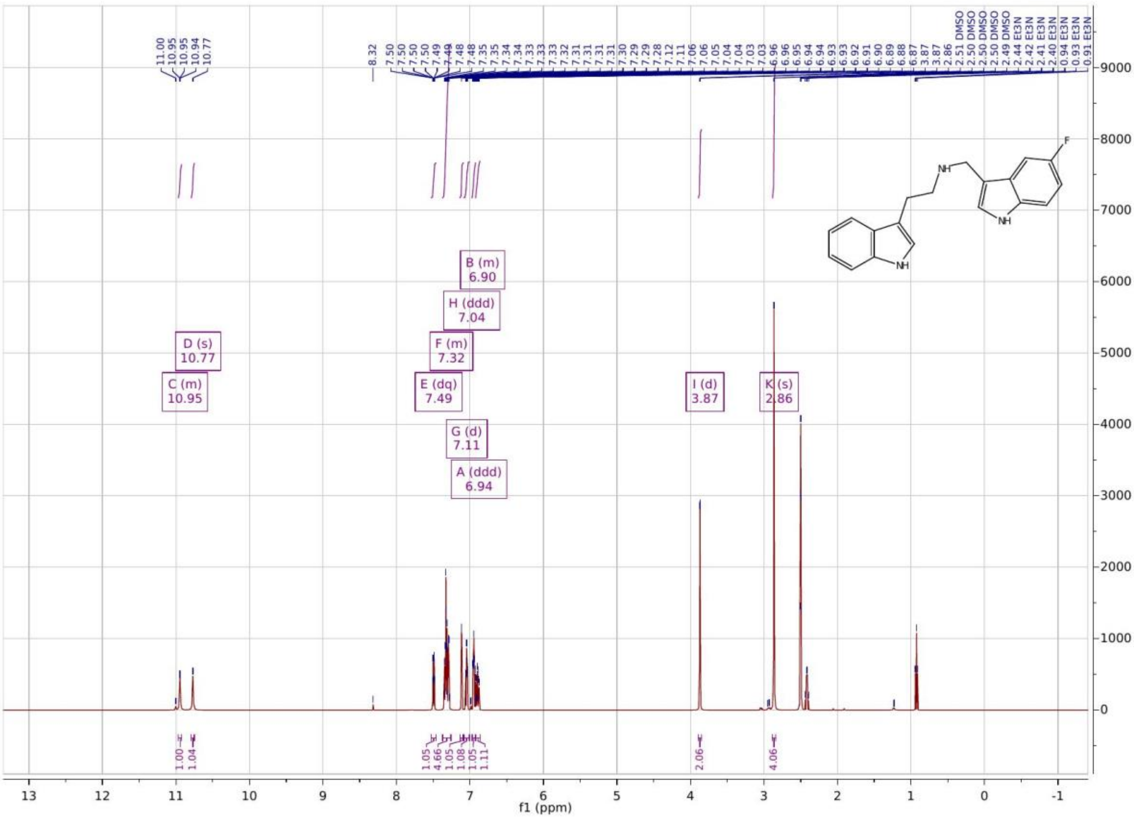

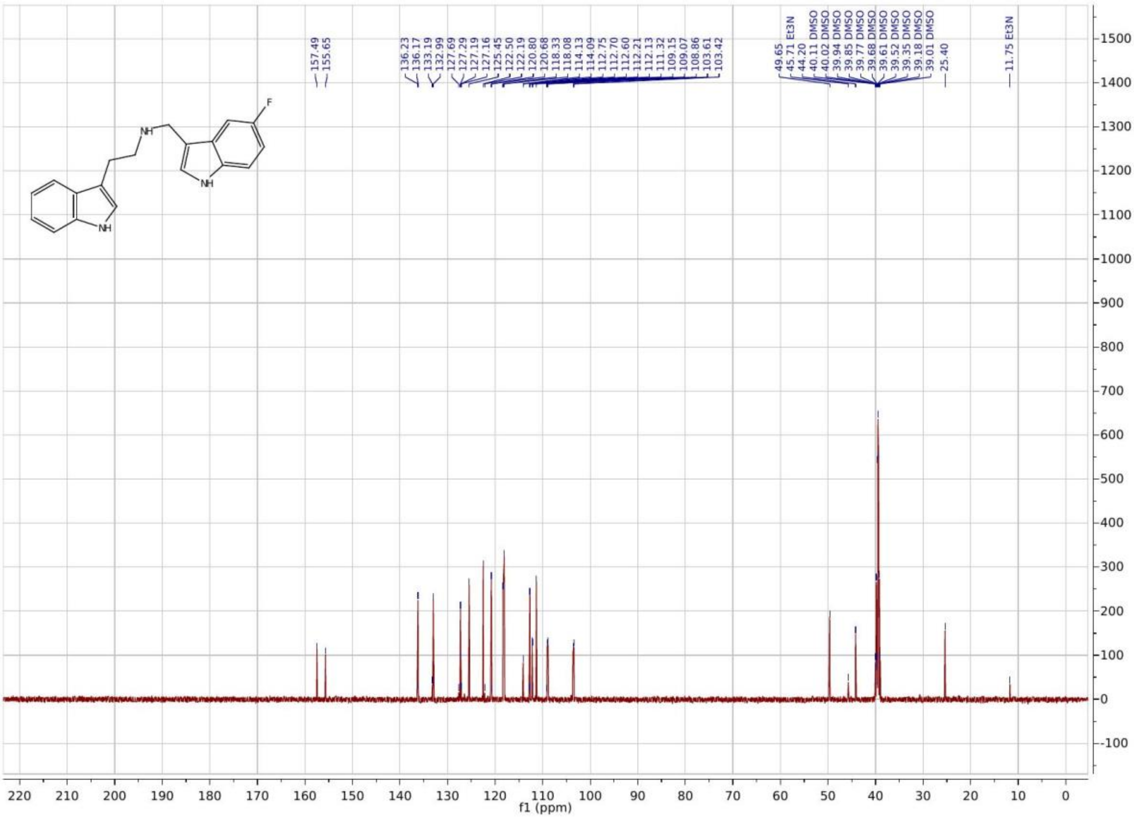

[(5-chloro-1*H*-indol-3-yl)methyl][2-(1*H*-indol-3-yl)ethyl]amine (11)

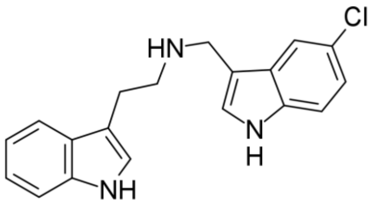

The product was obtained as a light brown solid. Yield: 56%.
LCMS [M+1] = 322,13 (324,13 calculated).

^{1}H NMR (500 MHz, DMSO-d_6) δ 11.06 (s, 1H), 10.78 (s, 1H), 7.68 – 7.63 (m, 1H), 7.60 (d, J = 2.0 Hz, 1H), 7.53 – 7.46 (m, 1H), 7.37 – 7.30 (m, 4H), 7.12 (dd, J = 10.2, 2.3 Hz, 2H), 7.08 – 7.01 (m, 4H), 3.88 (d, J = 0.9 Hz, 2H), 2.42 (s, 1H), 2.41 (s, 1H).

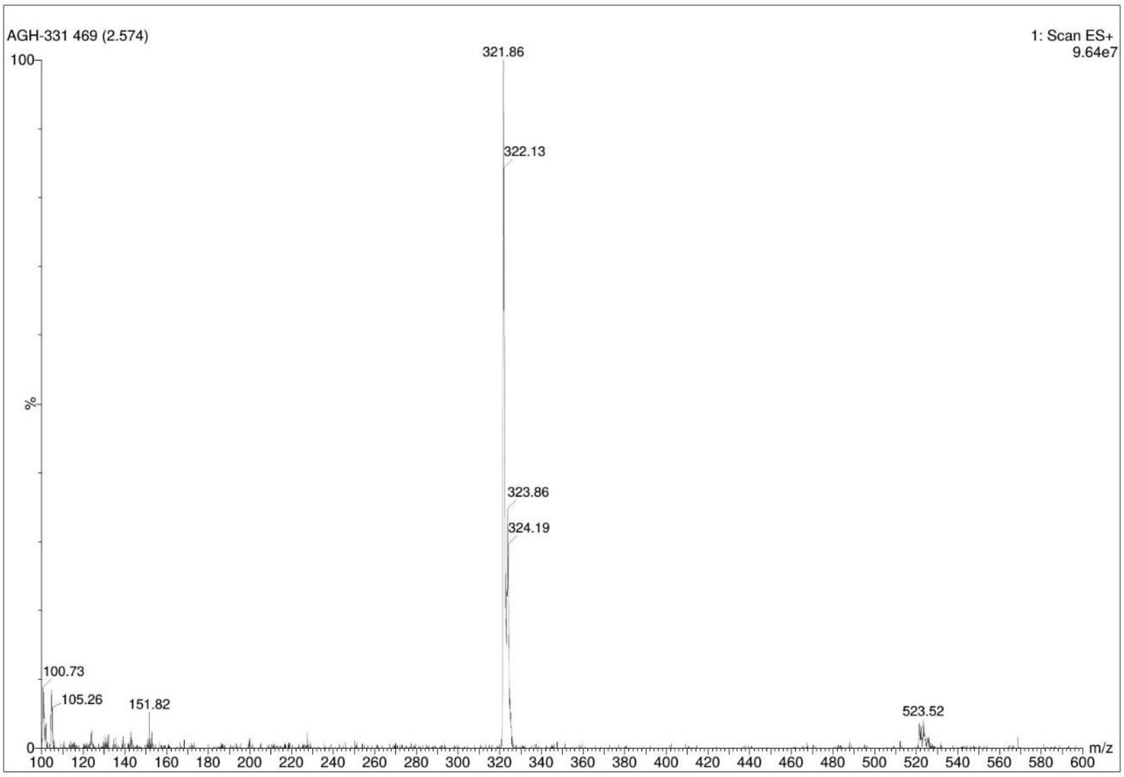

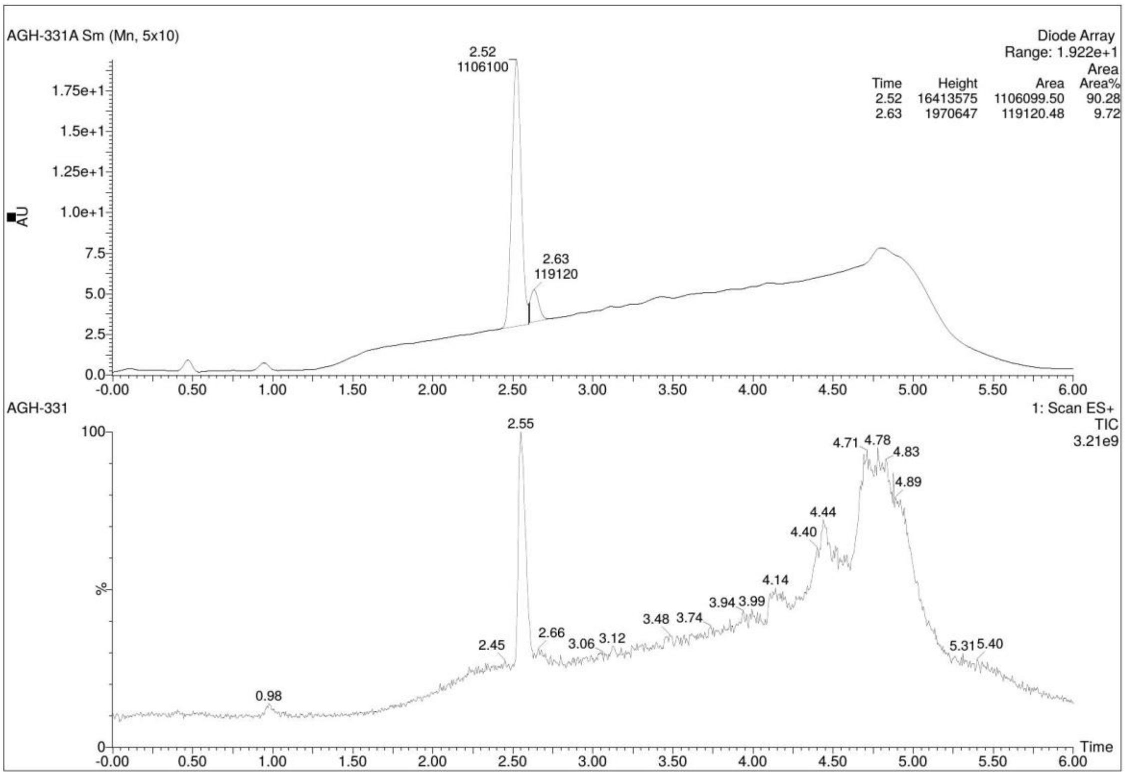

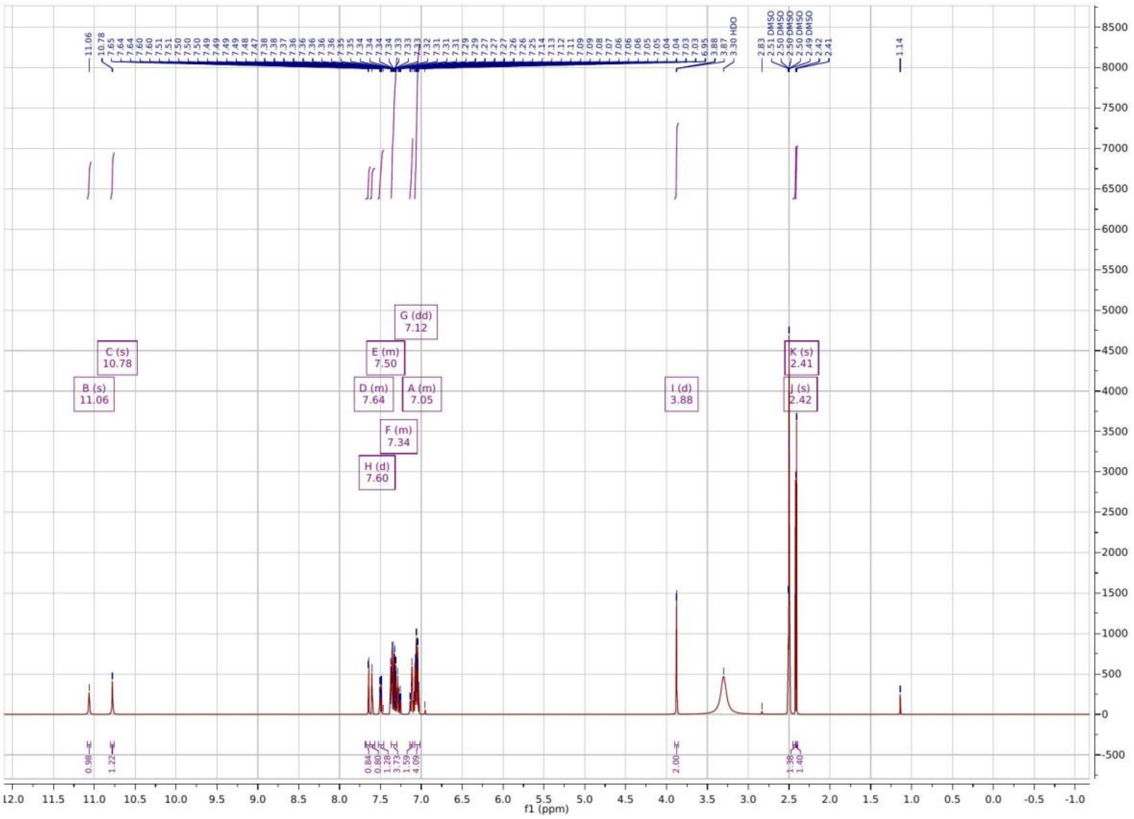

[2-(1*H*-indol-3-yl)ethyl][(5-iodo-1*H*-indol-3-yl)methyl]amine (12)

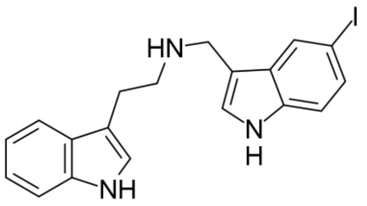

The product was obtained as a off-white solid. Yield: 67%.
LCMS [M+1] = 414,89 (416,06 calculated).

^{1}H NMR (500 MHz DMSO-d_6) δ 11.03 (s, 1H), 10.76 (s, 1H), 7.98 (d, *J* = 1.7 Hz, 1H), 7.54 – 7.47 (m, 1H), 7.36 – 7.27 (m, 3H), 7.25 – 7.17 (m, 2H), 7.11 (d, *J* = 2.4 Hz, 1H), 7.04 (ddd, *J* = 8.2, 7.0, 1.2 Hz, 1H), 6.95 (ddd, *J* = 7.9, 6.9, 1.1 Hz, 1H), 3.86 (d, *J* = 0.8 Hz, 2H), 2.85 (d, *J* = 2.7 Hz, 4H).

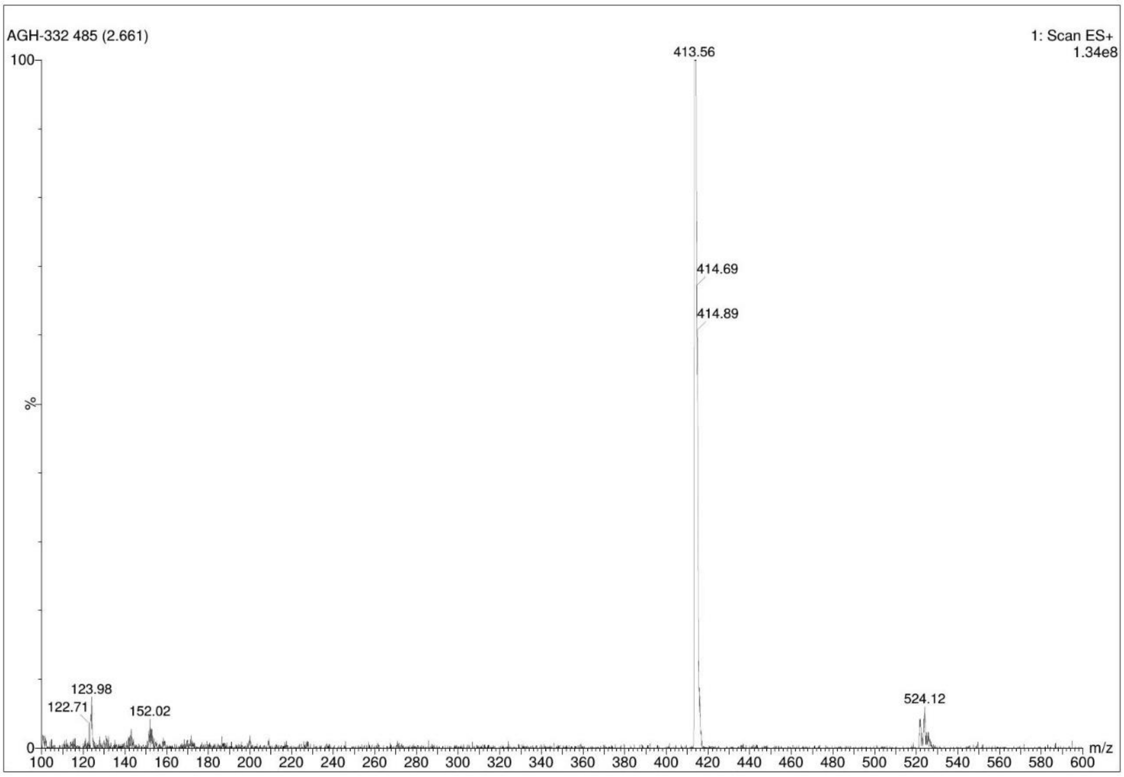

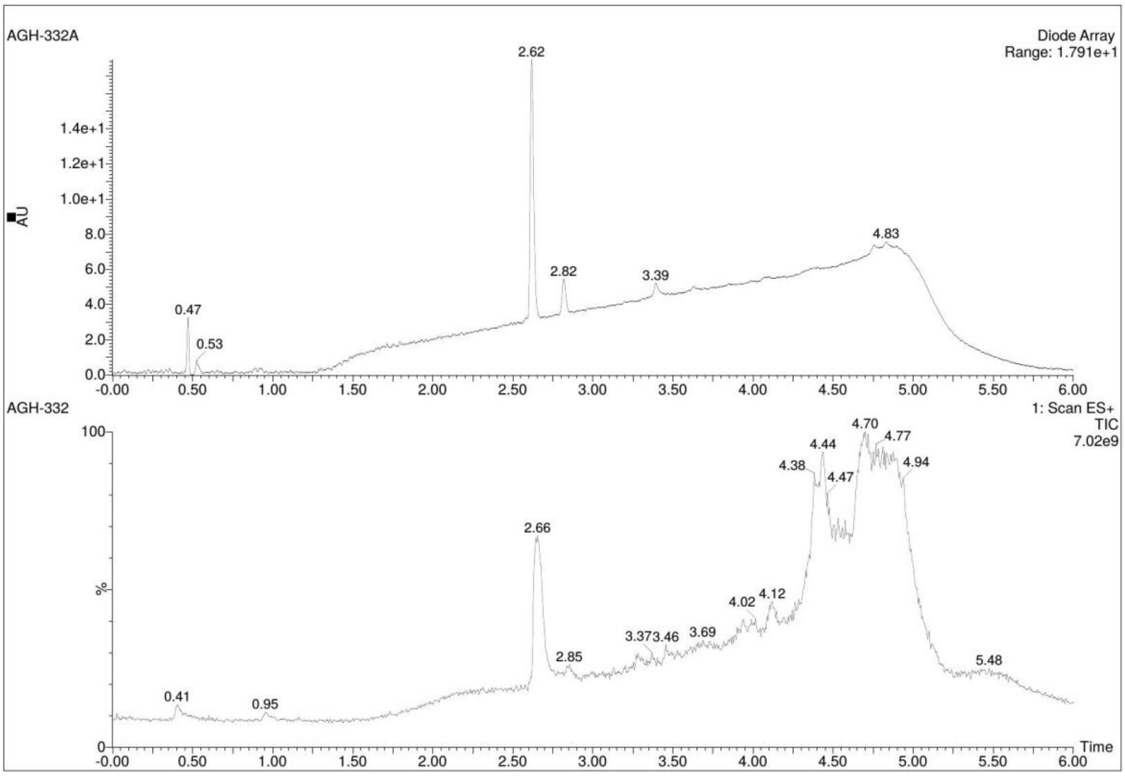

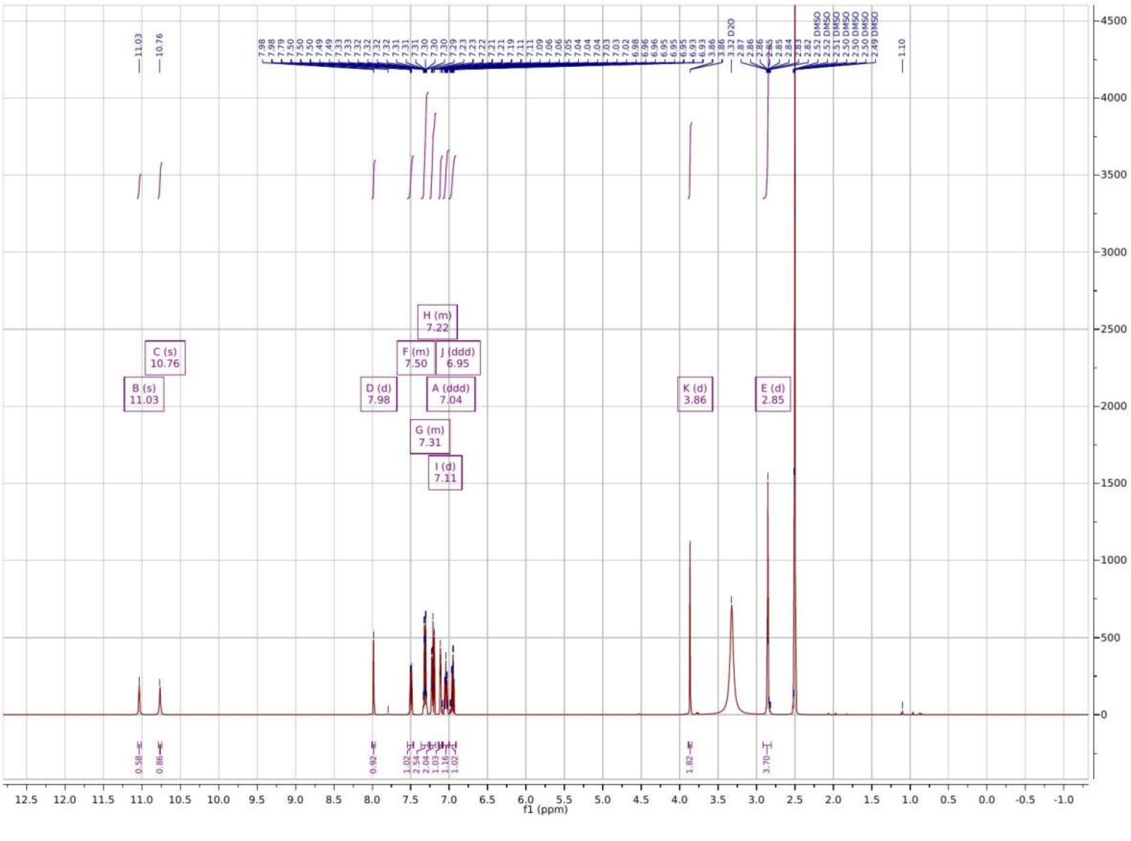

[(6-fluoro-1*H*-indol-3-yl)methyl][2-(1*H*-indol-3-yl)ethyl]amine (13)

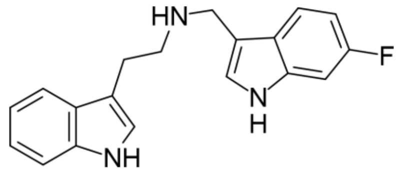

The product was obtained as a off-white solid. Yield: 40%.
LCMS [M+1] = 308,49 (308,16 calculated).

^{1}H NMR (500 MHz, DMSO-d_6) δ 11.51 (d, *J* = 2.5 Hz, 1H), 10.98 (d, *J* = 2.4 Hz, 1H), 7.75 (dd, *J* = 8.7, 5.4 Hz, 1H), 7.55 (d, *J* = 2.5 Hz, 1H), 7.51 (dd, *J* = 7.8, 0.9 Hz, 1H), 7.36 (dt, *J* = 8.1, 0.9 Hz, 1H), 7.22 (dd, *J* = 10.0, 2.4 Hz, 1H), 7.19 (d, *J* = 2.4 Hz, 1H), 7.08 (ddd, *J* = 8.1, 7.0, 1.2 Hz, 1H), 6.98 (ddd, *J* = 7.9, 7.0, 1.0 Hz, 1H), 6.94 (ddd, *J* = 9.7, 8.7, 2.4 Hz, 1H), 4.35 (s, 2H), 3.22 – 3.15 (m, 2H), 3.07 (dd, *J* = 9.8, 6.4 Hz, 2H).
^{13}C NMR (126 MHz, DMSO-d_6) δ 164.85, 159.97, 158.10, 136.26, 135.90, 135.80, 127.93, 127.90, 126.73, 123.58, 123.23, 121.16, 119.86, 119.78, 118.45, 118.11, 111.56, 109.50, 107.81, 107.62, 105.43, 97.83, 97.62, 46.48, 41.09, 21.73.

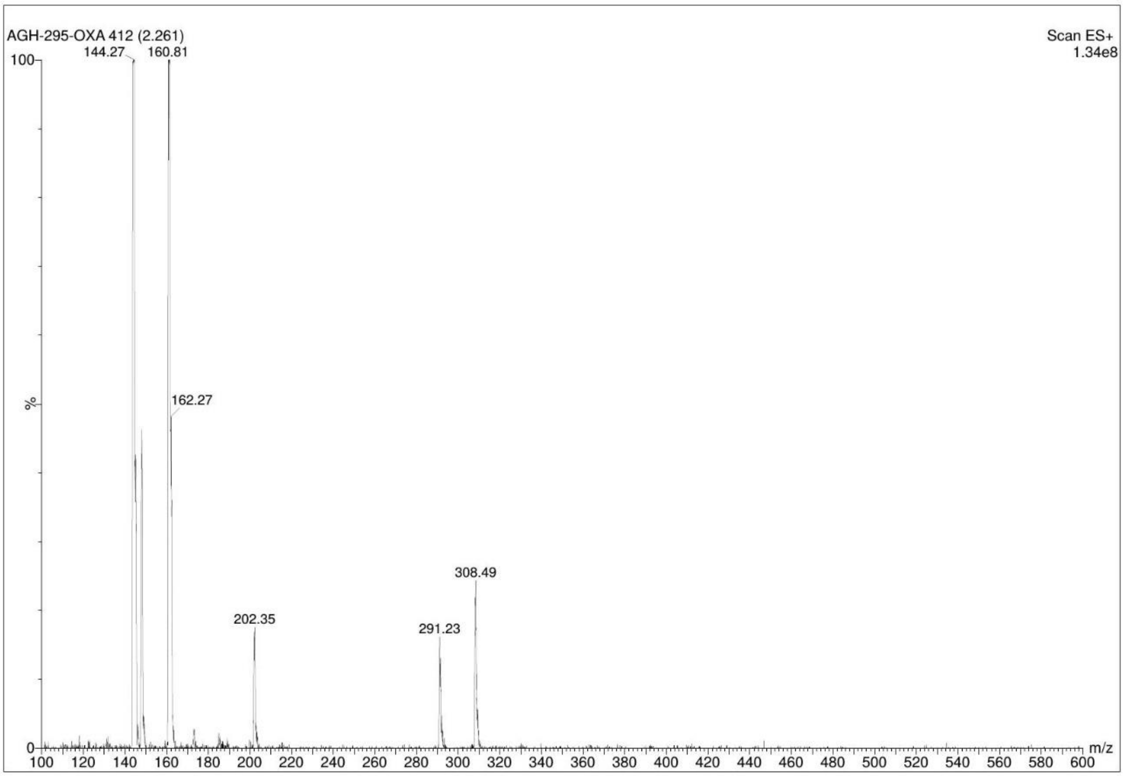

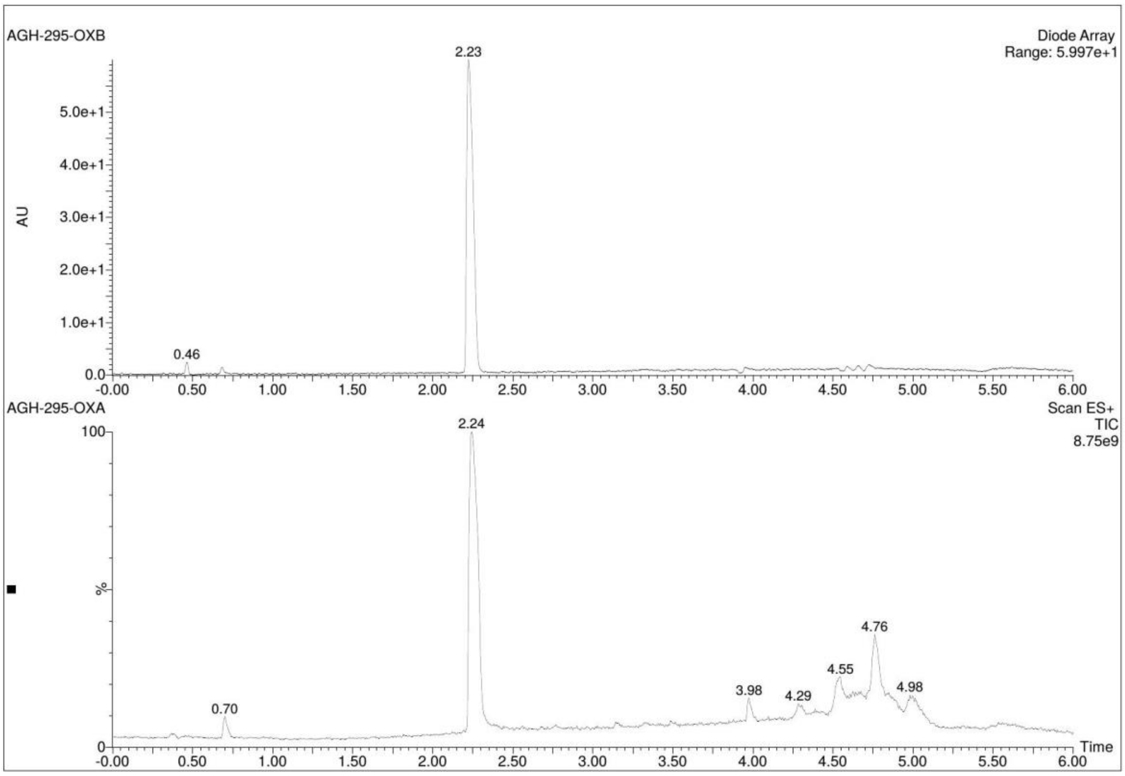

AGH-295-OXB
Diode Array
Range: 5.997e+1
AU
2.23
0.46
AGH-295-OXA
Scan ES+
TIC
8.75e9
%
2.24
0.70
3.98
4.29
4.55
4.76
4.98
Time

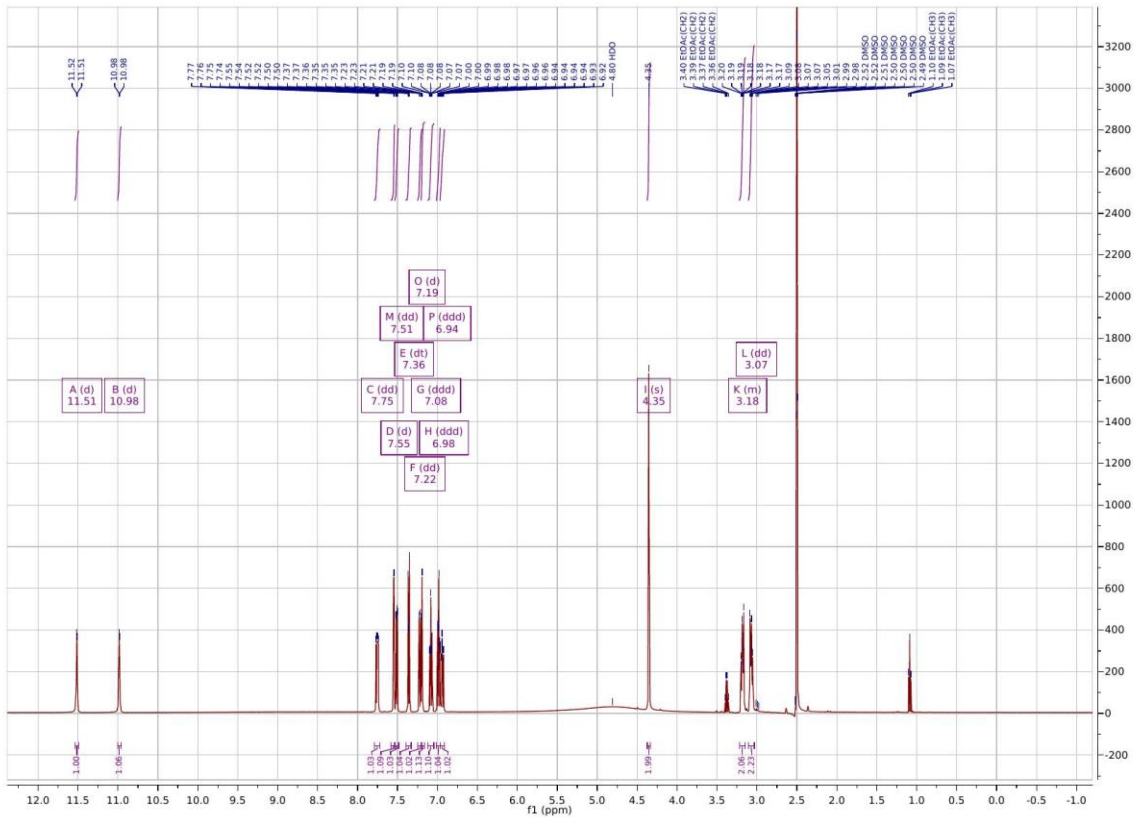

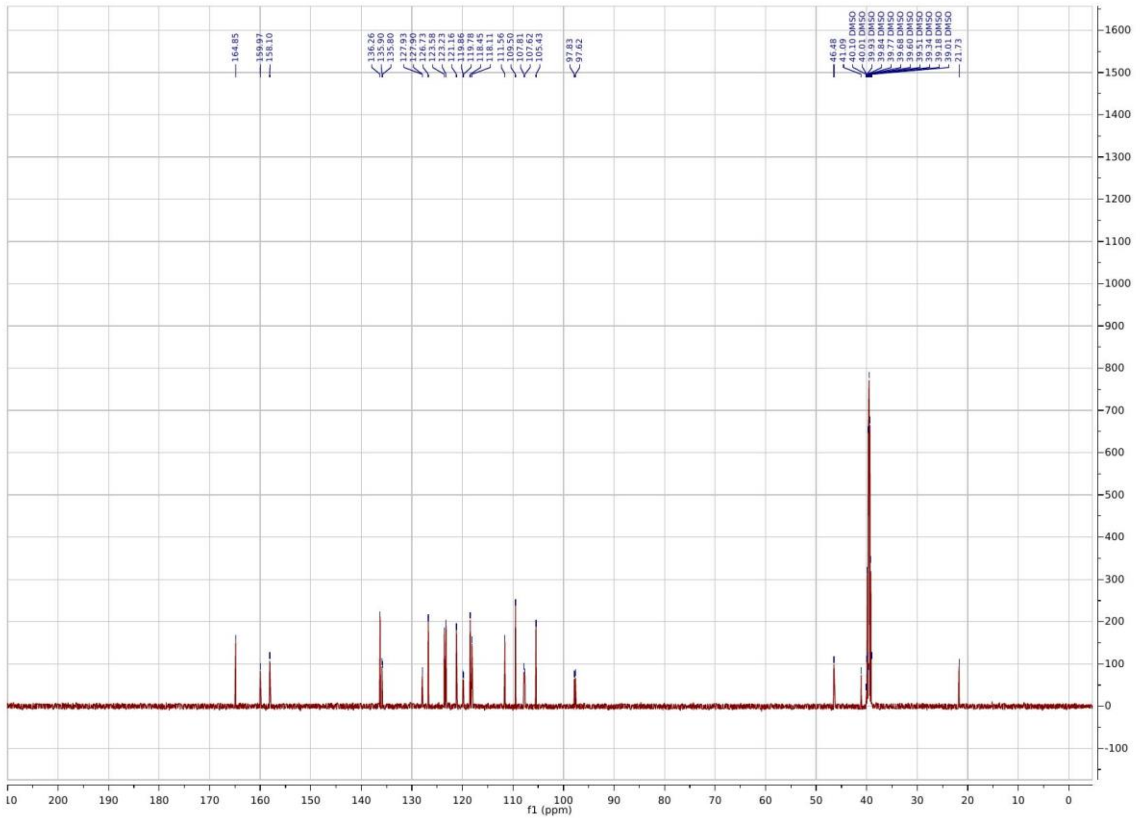

[(6-chloro-1*H*-indol-3-yl)methyl][2-(1*H*-indol-3-yl)ethyl]amine (14)

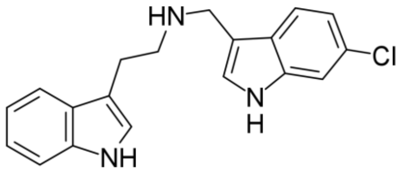

The product was obtained as a white solid. Yield: 55%.
LCMS [M+1] = 324,23 (324,13 calculated).

^{1}H NMR (500 MHz, DMSO-d_6) δ 11.61 (d, *J* = 2.5 Hz, 1H), 10.98 (d, *J* = 2.4 Hz, 1H), 7.77 (d, *J* = 8.5 Hz, 1H), 7.59 (d, *J* = 2.5 Hz, 1H), 7.54 – 7.47 (m, 2H), 7.36 (dt, *J* = 8.1, 0.9 Hz, 1H), 7.19 (d, *J* = 2.4 Hz, 1H), 7.12 – 7.05 (m, 2H), 6.98 (ddd, *J* = 8.0, 7.0, 1.0 Hz, 1H), 4.36 (s, 2H), 3.22 – 3.14 (m, 2H), 3.07 (dd, *J* = 9.8, 6.4 Hz, 2H).
^{13}C NMR (126 MHz, DMSO-d_6) δ 164.87, 136.36, 136.26, 128.43, 126.73, 126.34, 125.61, 123.23, 121.16, 120.15, 119.49, 118.45, 118.11, 111.56, 111.40, 109.49, 105. 55, 46.49, 40.98, 21.74.

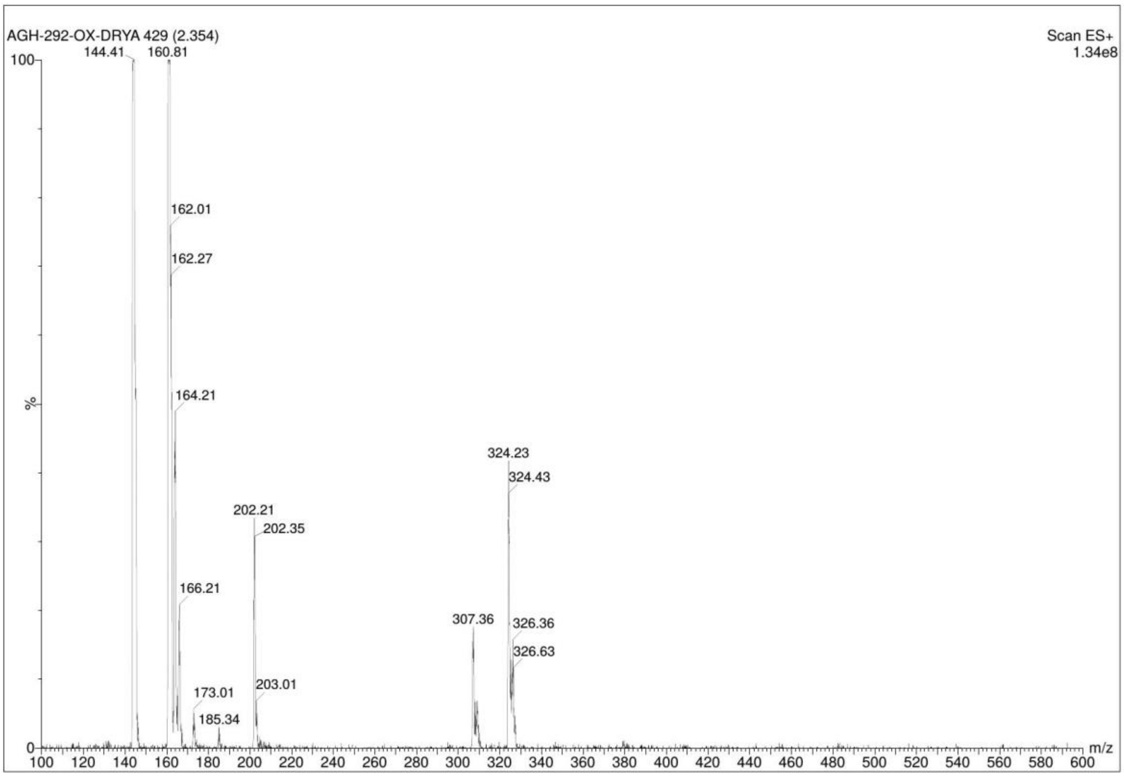

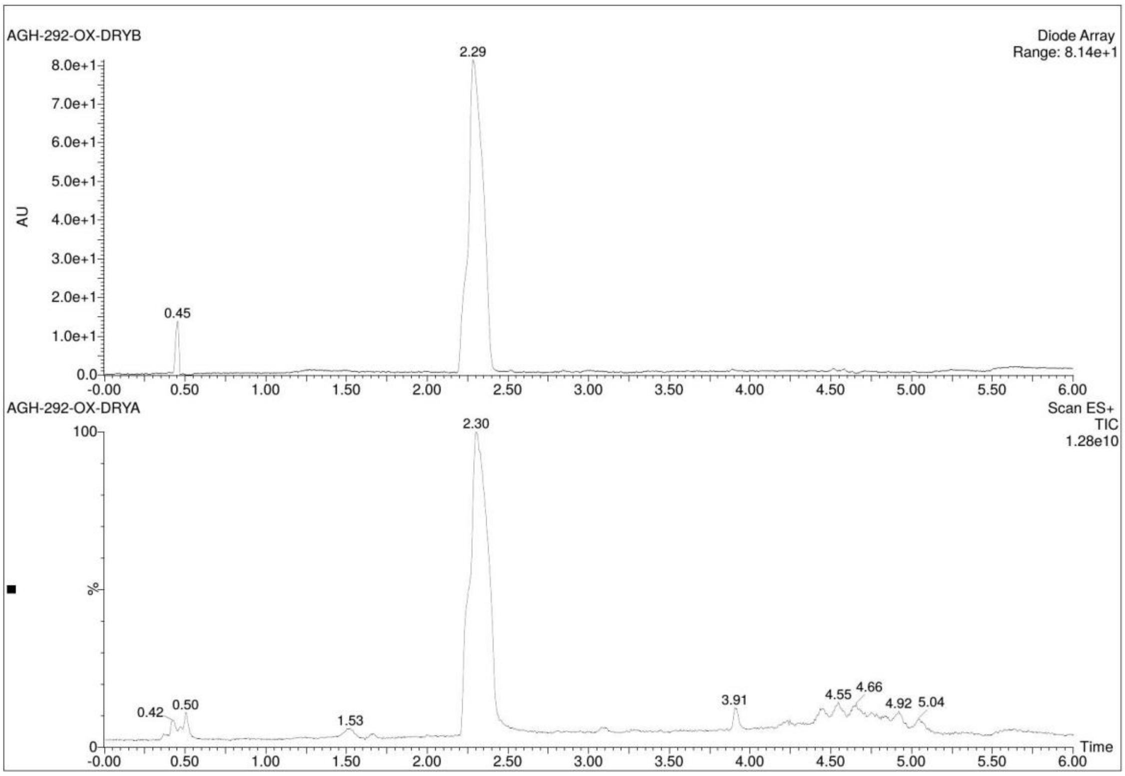
AGH-292-OX-DRYB
Diode Array
Range: 8.14e+1
AU
AGH-292-OX-DRYA
Scan ES+
TIC
1.28e10
Time

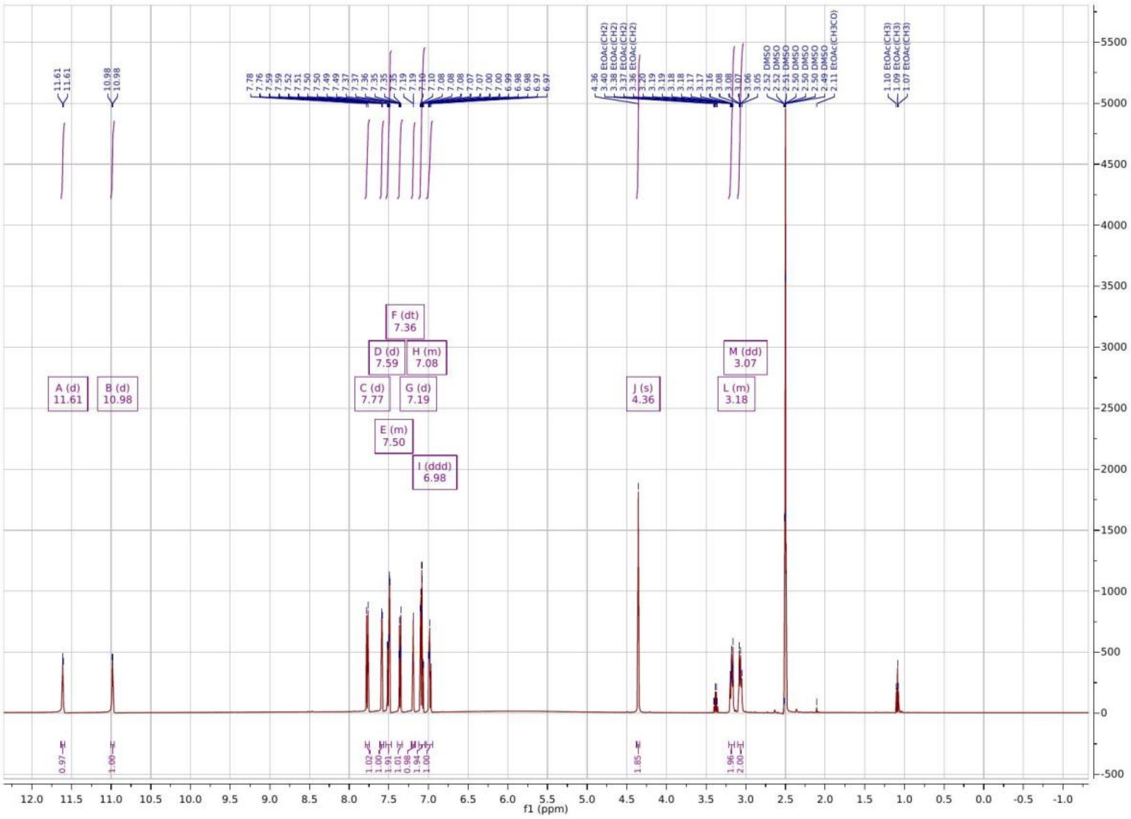
A (d)
11.61
B (d)
10.98
C (d)
7.77
D (d)
7.59
E (m)
7.50
F (dt)
7.36
G (d)
7.19
H (m)
7.08
I (ddd)
6.98
J (s)
4.36
L (m)
3.18
M (dd)
3.07
f1 (ppm)

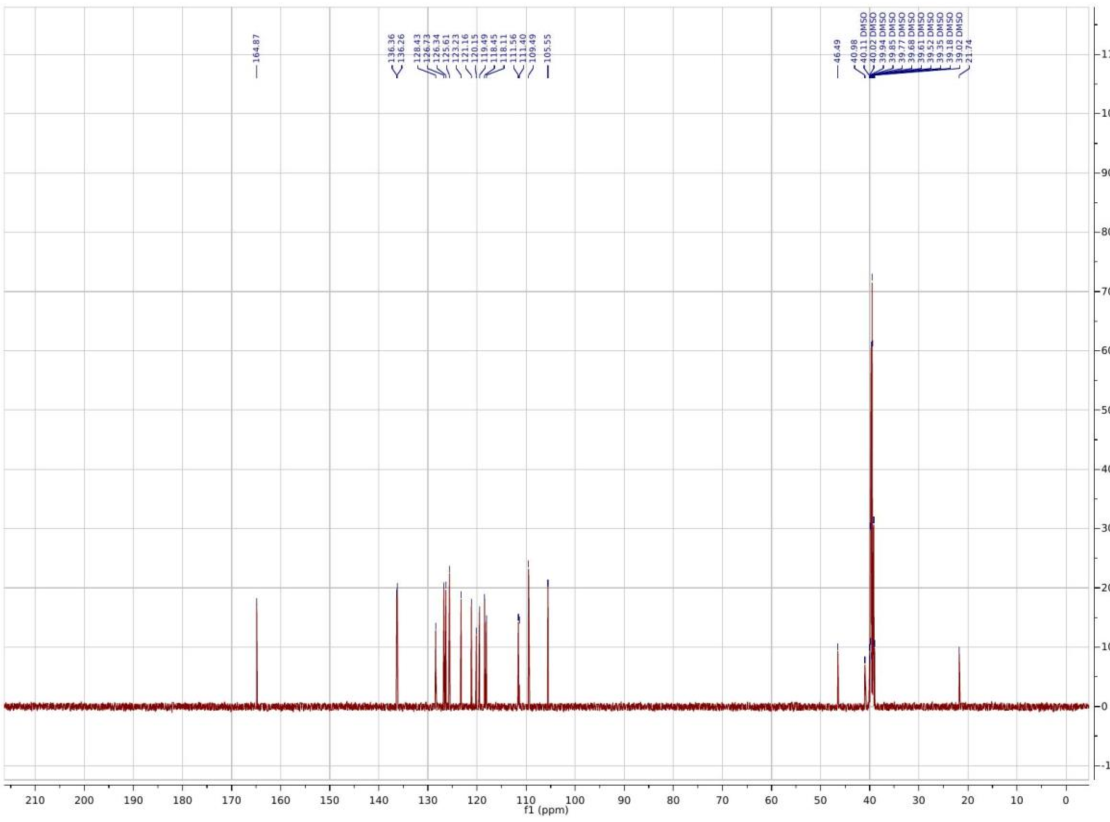

[(6-bromo-1*H*-indol-3-yl)methyl][2-(1*H*-indol-3-yl)ethyl]amine (15)

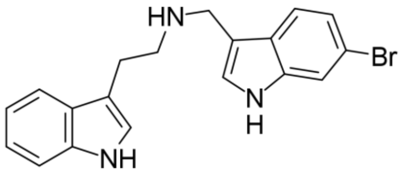

The product was obtained as a dirty pink solid. Yield: 35%.
LCMS [M+1] = 367,77 (368,08 calculated).

^{1}H NMR (500 MHz, DMSO-d_6) δ 11.58 (d, *J* = 2.6 Hz, 1H), 10.98 (d, *J* = 2.5 Hz, 1H), 9.25 (s, 2H), 7.77 (d, *J* = 8.5 Hz, 1H), 7.64 (dd, *J* = 8.0, 2.2 Hz, 2H), 7.54 (dd, *J* = 7.8, 1.0 Hz, 1H), 7.36 (dt, *J* = 8.2, 1.0 Hz, 1H), 7.24 – 7.18 (m, 2H), 7.08 (ddd, *J* = 8.1, 6.9, 1.2 Hz, 1H), 6.99 (ddd, *J* = 8.0, 7.0, 1.0 Hz, 1H), 4.33 (t, *J* = 5.5 Hz, 2H), 3.19 – 3.07 (m, 4H).
^{13}C NMR (126 MHz, DMSO-d_6) δ 136.78, 136.24, 128.50, 126.72, 125.81, 123.19, 122.02, 121.16, 120.67, 118.43, 118.16, 114.34, 114.29, 111.54, 109.45, 105.40, 46.42, 40.86, 25.50, 21.67, 15.18.

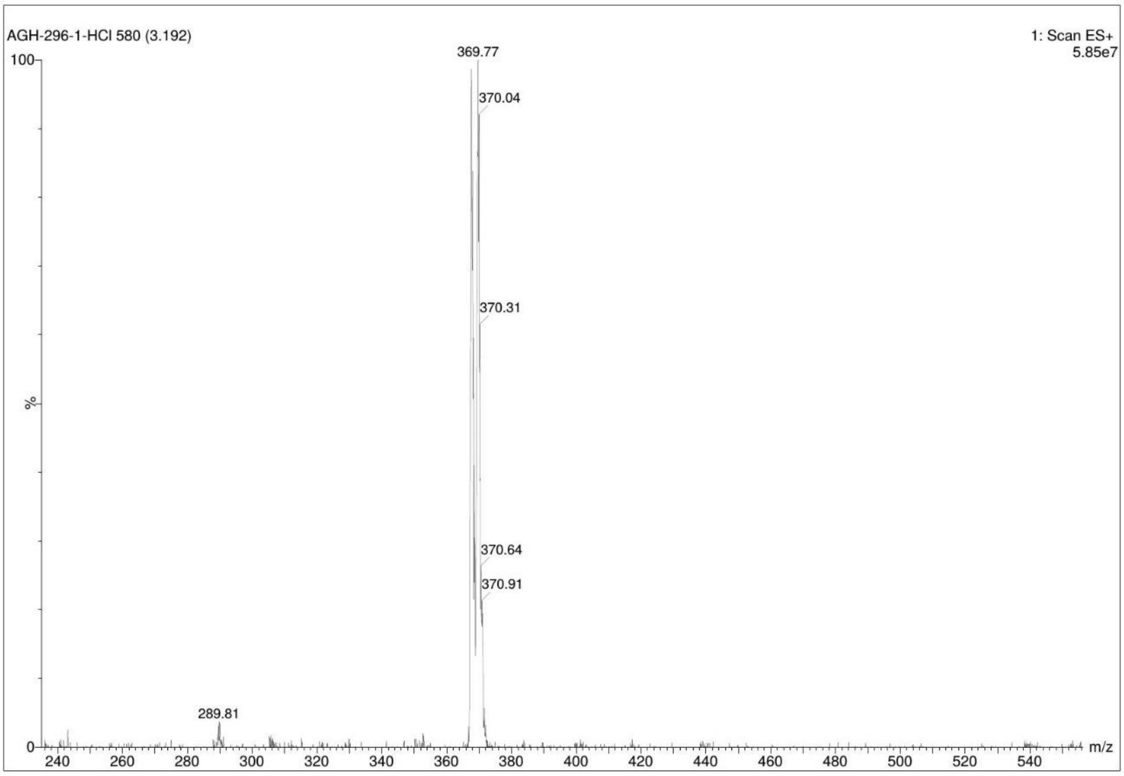
AGH-296-1-HCl 580 (3.192)
1: Scan ES+
5.85e7
369.77
370.04
370.31
370.64
370.91
289.81
%
m/z

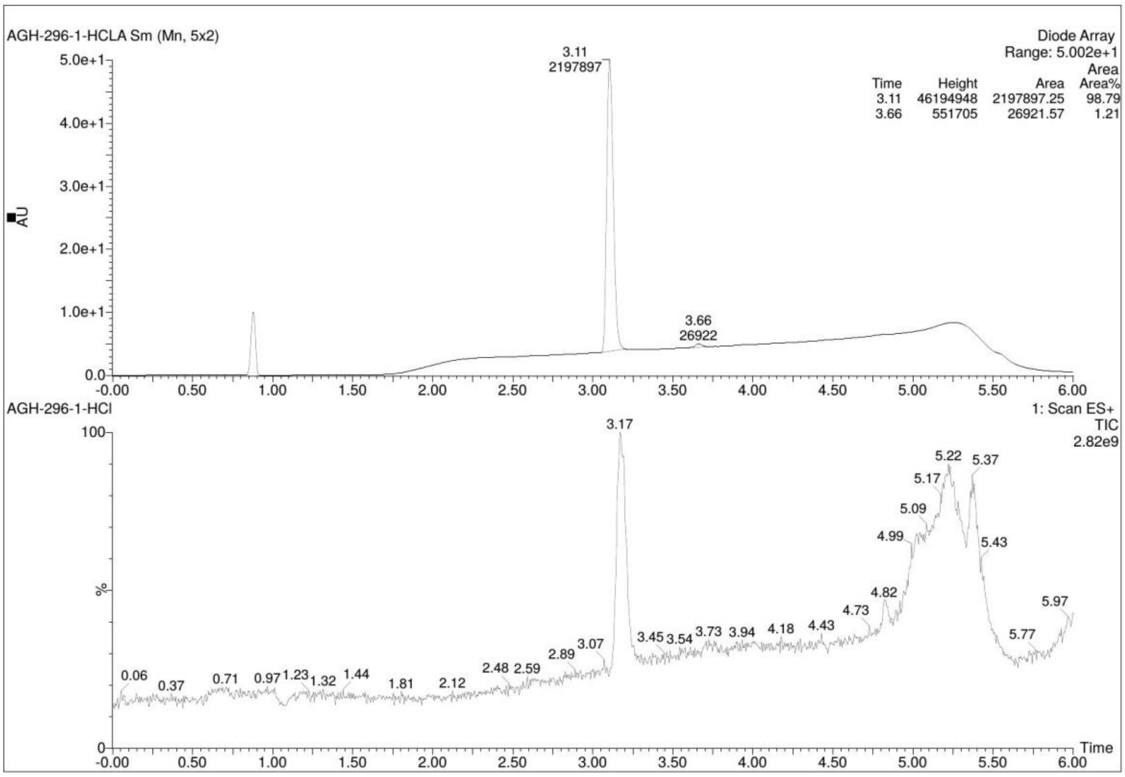
AGH-296-1-HCLA Sm (Mn, 5x2)
Diode Array
Range: 5.002e+1
Time Height Area Area%
3.11 46194948 2197897.25 98.79
3.66 551705 26921.57 1.21
3.11
2197897
3.66
26922
AU
AGH-296-1-HCl
1: Scan ES+
TIC
2.82e9
3.17
%
Time

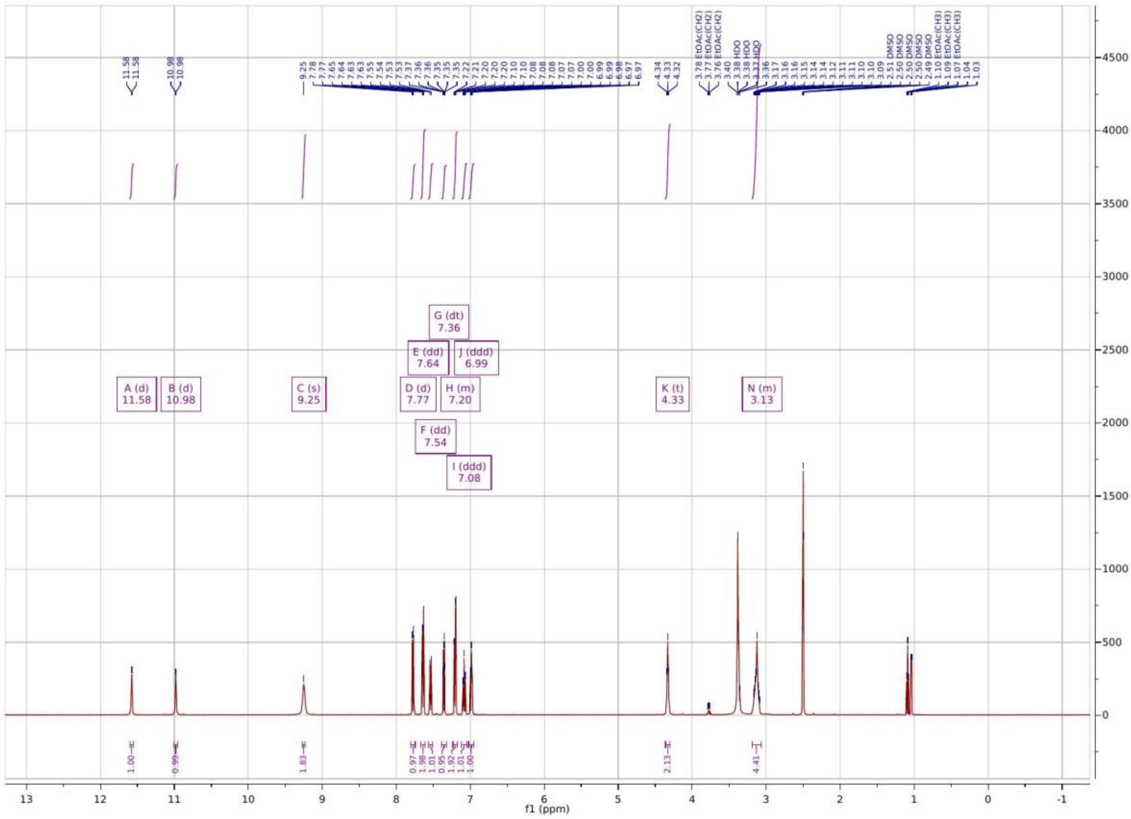

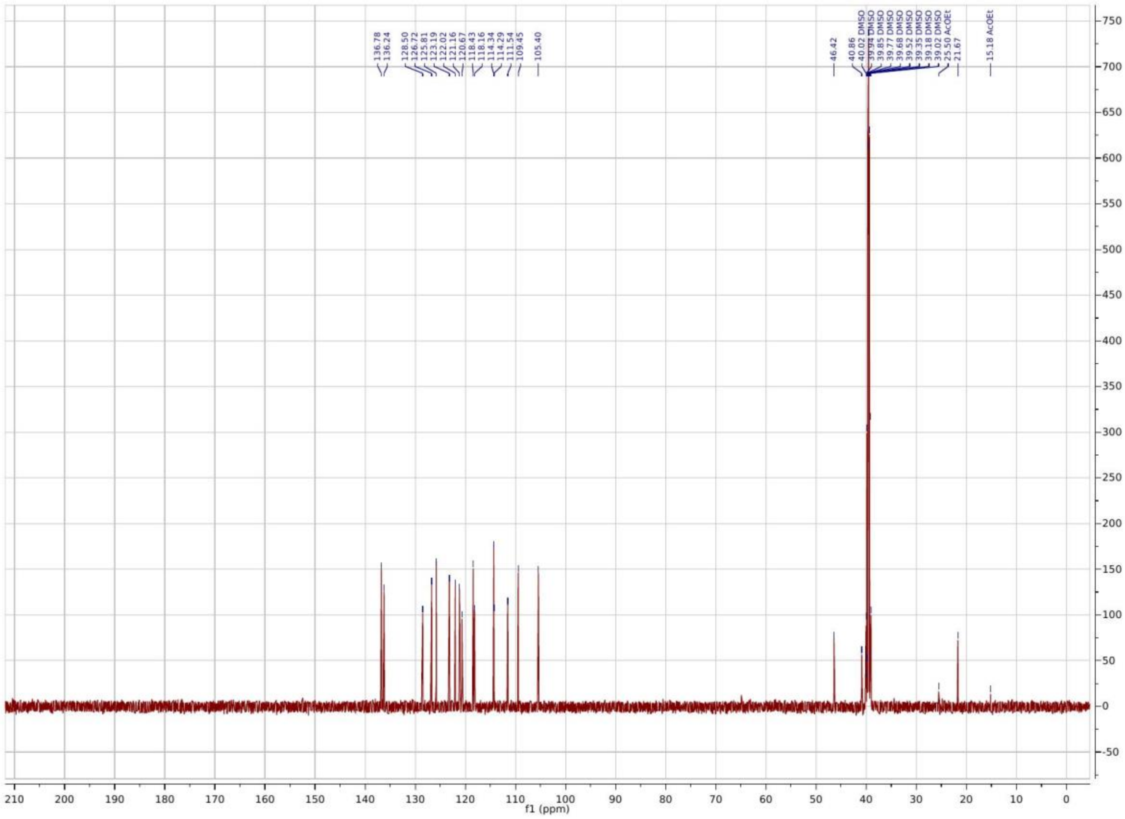

[(7-fluoro-1*H*-indol-3-yl)methyl][2-(1*H*-indol-3-yl)ethyl]amine (16)

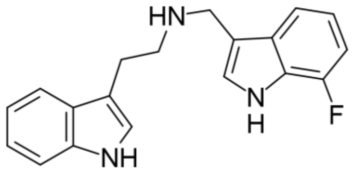

The product was obtained as a white solid. Yield: 39%.
LCMS [M+1] = 308,43 (308,16 calculated).

^{1}H NMR (500 MHz, DMSO-d_6) δ 11.90 (d, J = 2.4 Hz, 1H), 10.99 – 10.94 (m, 1H), 7.61 (d, J = 2.5 Hz, 1H), 7.59 (d, J = 7.8 Hz, 1H), 7.51 (dt, J = 7.9, 0.9 Hz, 1H), 7.36 (dt, J = 8.1, 0.9 Hz, 1H), 7.20 (d, J = 2.3 Hz, 1H), 7.11 – 7.02 (m, 2H), 7.02 – 6.94 (m, 2H), 4.37 (s, 2H), 3.19 (dd, J = 9.8, 6.4 Hz, 2H), 3.07 (dd, J = 9.8, 6.5 Hz, 2H).
^{13}C NMR (126 MHz, DMSO-d_6) δ 164.67, 150.15, 148.21, 136.25, 130.75, 130.71, 128.34, 126.72, 123.83, 123.72, 123.23, 121.16, 119.62, 119.57, 118.44, 118.09, 114.96, 114.93, 111.55, 109.44, 106.51, 106.42, 106.40, 46.53, 41.08, 21.74.

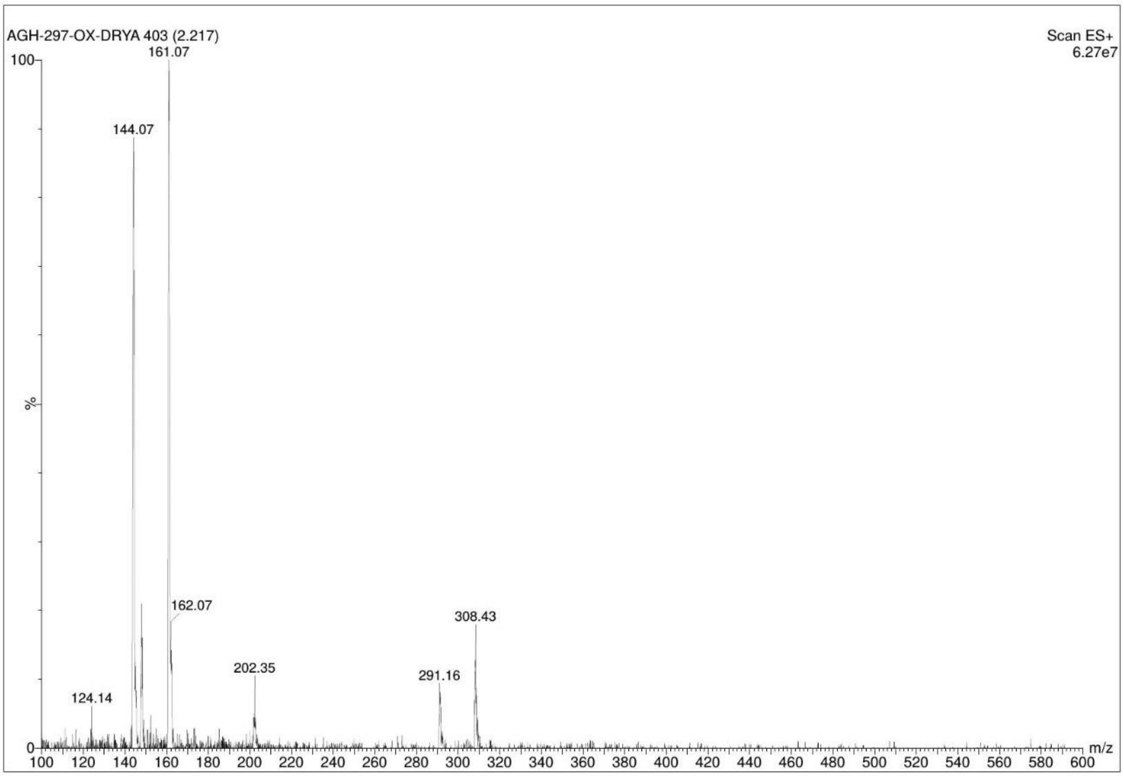

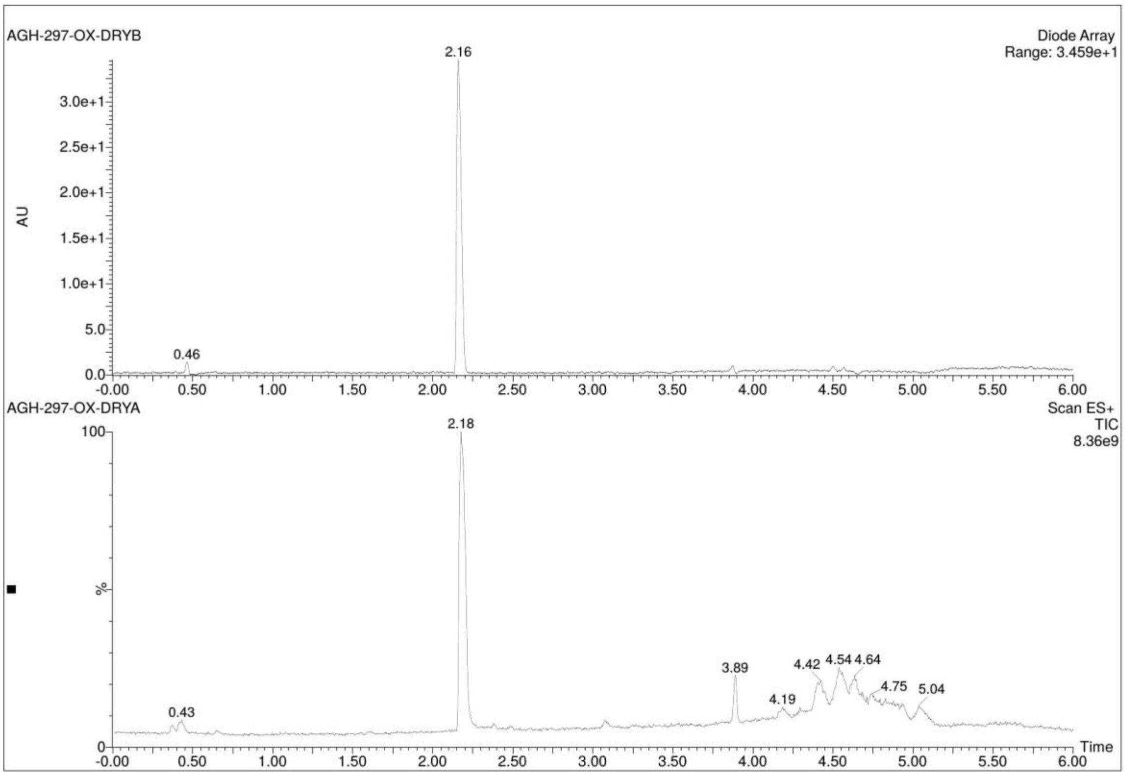
AGH-297-OX-DRYB
Diode Array
Range: 3.459e+1
AU
2.16
0.46
AGH-297-OX-DRYA
Scan ES+
TIC
8.36e9
2.18
0.43
3.89
4.19
4.42
4.54
4.64
4.75
5.04
%
Time

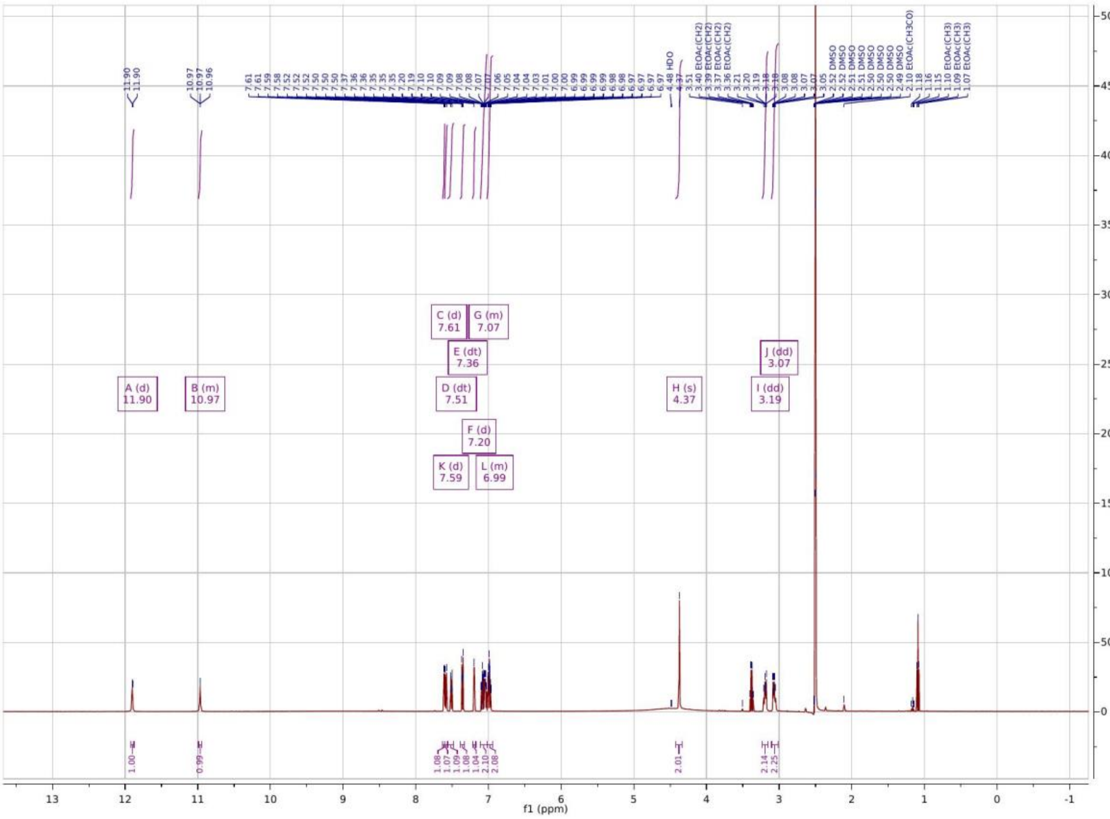
A (d)
11.90
B (m)
10.97
C (d)
7.61
G (m)
7.07
E (dt)
7.36
D (dt)
7.51
F (d)
7.20
K (d)
7.59
L (m)
6.99
H (s)
4.37
J (dd)
3.07
I (dd)
3.19
f1 (ppm)

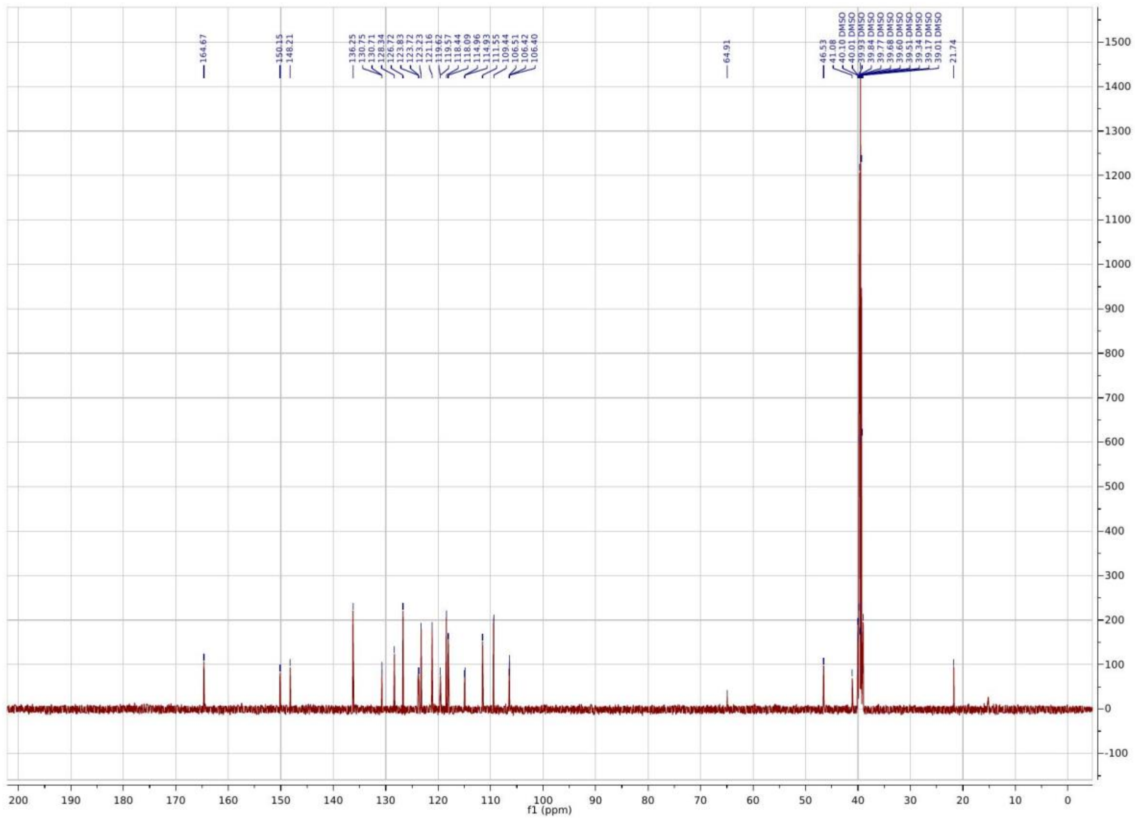

[(7-chloro-1*H*-indol-3-yl)methyl][2-(1*H*-indol-3-yl)ethyl]amine (17)

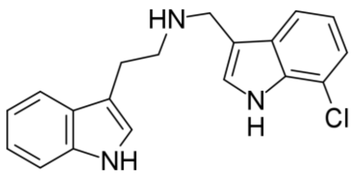

The product was obtained as a white solid. Yield: 55%.
LCMS [M+1] = 323,53 (324,13 calculated).

^{1}H NMR (500 MHz, DMSO-d_6) δ 11.77 (s, 1H), 10.99 (s, 1H), 7.75 (d, *J* = 7.8 Hz, 1H), 7.64 (d, *J* = 2.5 Hz, 1H), 7.51 (dd, *J* = 7.8, 1.0 Hz, 1H), 7.36 (dt, *J* = 8.0, 0.9 Hz, 1H), 7.23 (dd, *J* = 7.6, 0.9 Hz, 1H), 7.19 (d, *J* = 2.4 Hz, 1H), 7.12 – 7.04 (m, 2H), 6.98 (ddd, *J* = 7.9, 7.0, 1.0 Hz, 1H), 4.38 (s, 2H), 3.17 (s, 4H), 3.08 (dd, *J* = 9.8, 6.4 Hz, 2H).
^{13}C NMR (126 MHz, DMSO-d_6) δ 164.93, 136.27, 132.76, 128.79, 128.52, 126.74, 123.23, 121.15, 120.25, 118.45, 118.11, 117.86, 116.07, 111.56, 109.52, 106.72, 48.62, 46.53, 41.02, 21.75.

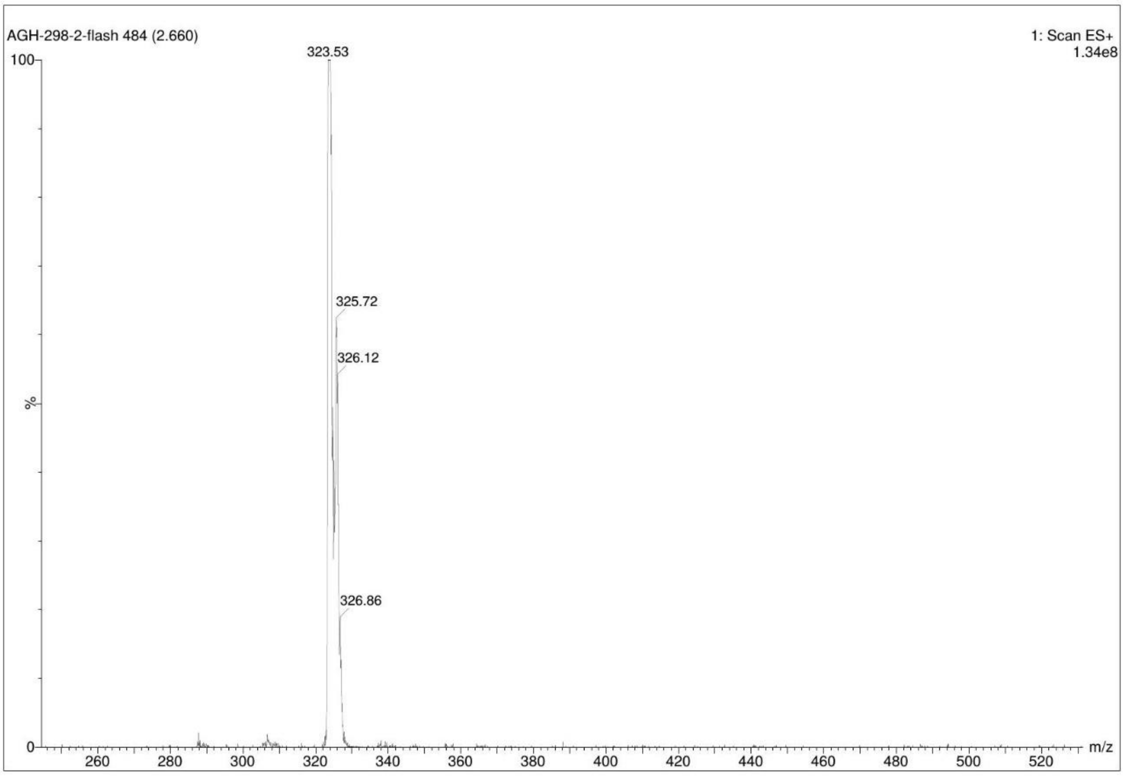

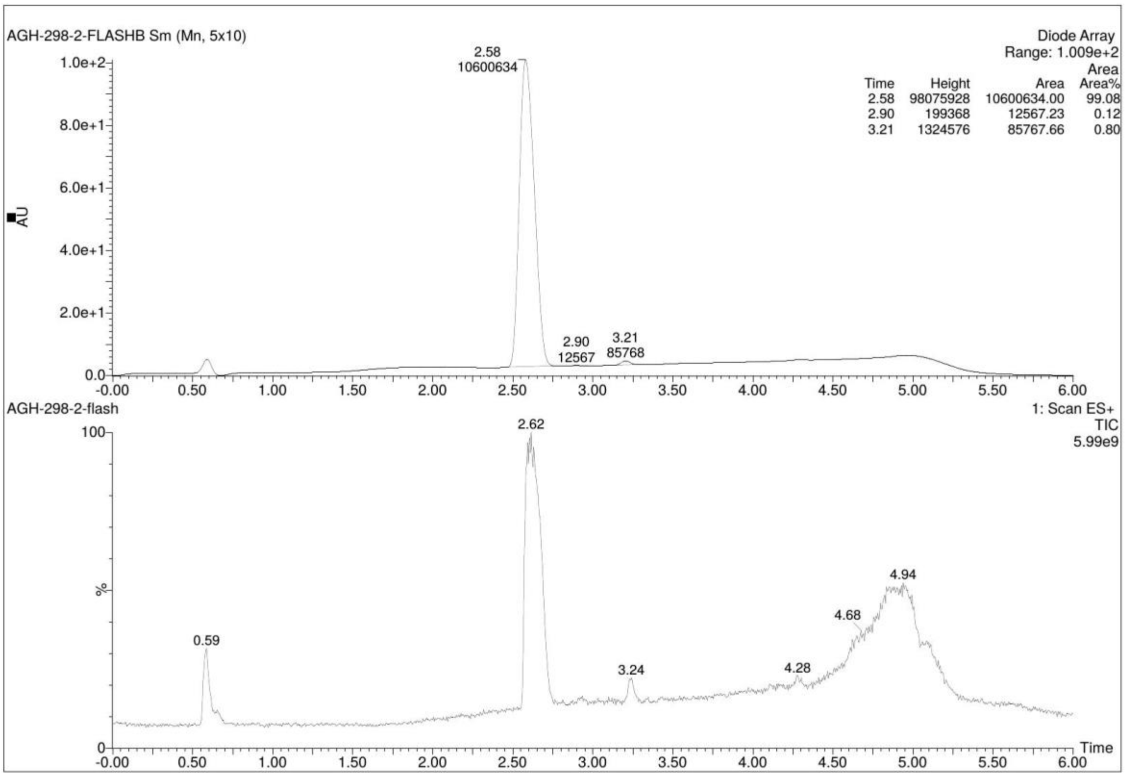

Time	Height	Area	Area Area%
2.58	98075928	10600634.00	99.08
2.90	199368	12567.23	0.12
3.21	1324576	85767.66	0.80

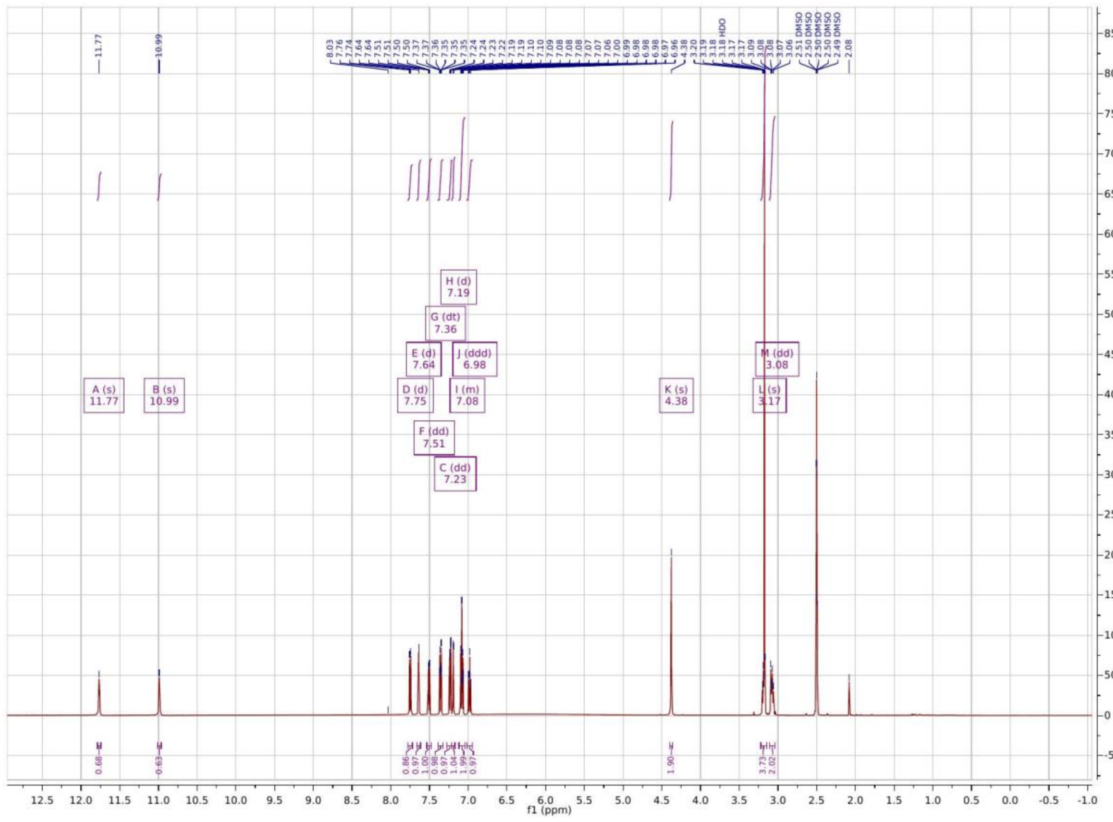

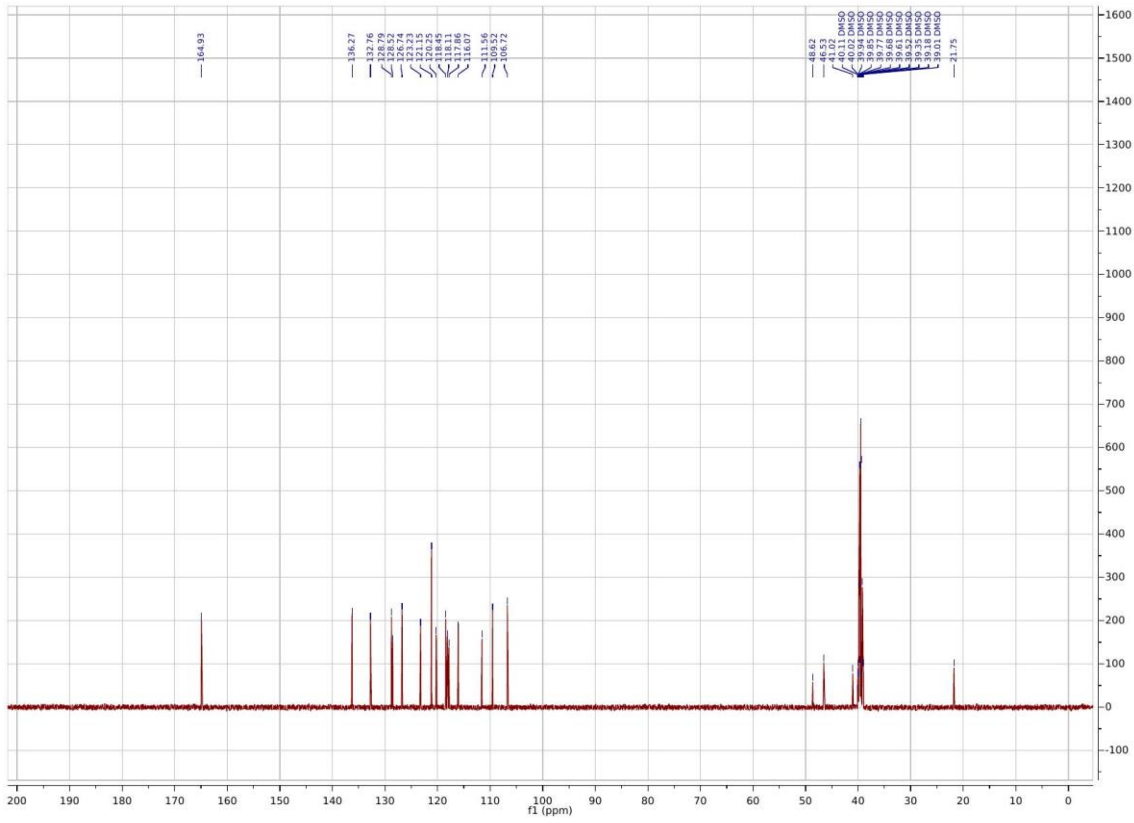

[(7-bromo-1*H*-indol-3-yl)methyl][2-(1*H*-indol-3-yl)ethyl]amine (18)

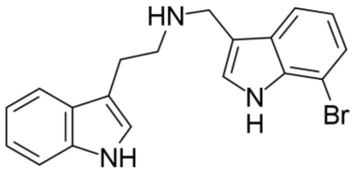

The product was obtained as a off-white solid. Yield: 50%.
LCMS [M+1] = 367,77 (368,08 calculated).

^{1}H NMR (500 MHz, DMSO-d_6) δ 11.60 (d, *J* = 3.0 Hz, 1H), 11.00 – 10.96 (m, 1H), 9.29 (s, 2H), 7.83 (d, *J* = 7.9 Hz, 1H), 7.71 (d, *J* = 2.7 Hz, 1H), 7.54 (d, *J* = 7.9 Hz, 1H), 7.36 (ddd, *J* = 9.4, 7.4, 0.9 Hz, 2H), 7.20 (d, *J* = 2.4 Hz, 1H), 7.12 – 6.95 (m, 3H), 4.35 (t, *J* = 5.1 Hz, 2H), 3.14 (p, *J* = 8.6, 8.1 Hz, 4H).
^{13}C NMR (126 MHz, DMSO-d_6) δ 136.24, 134.19, 128.73, 128.49, 126.73, 124.21, 123.20, 121.16, 120.65, 118.43, 118.16, 111.53, 109.44, 106.56, 104.30, 46.45, 40.98, 21.67.

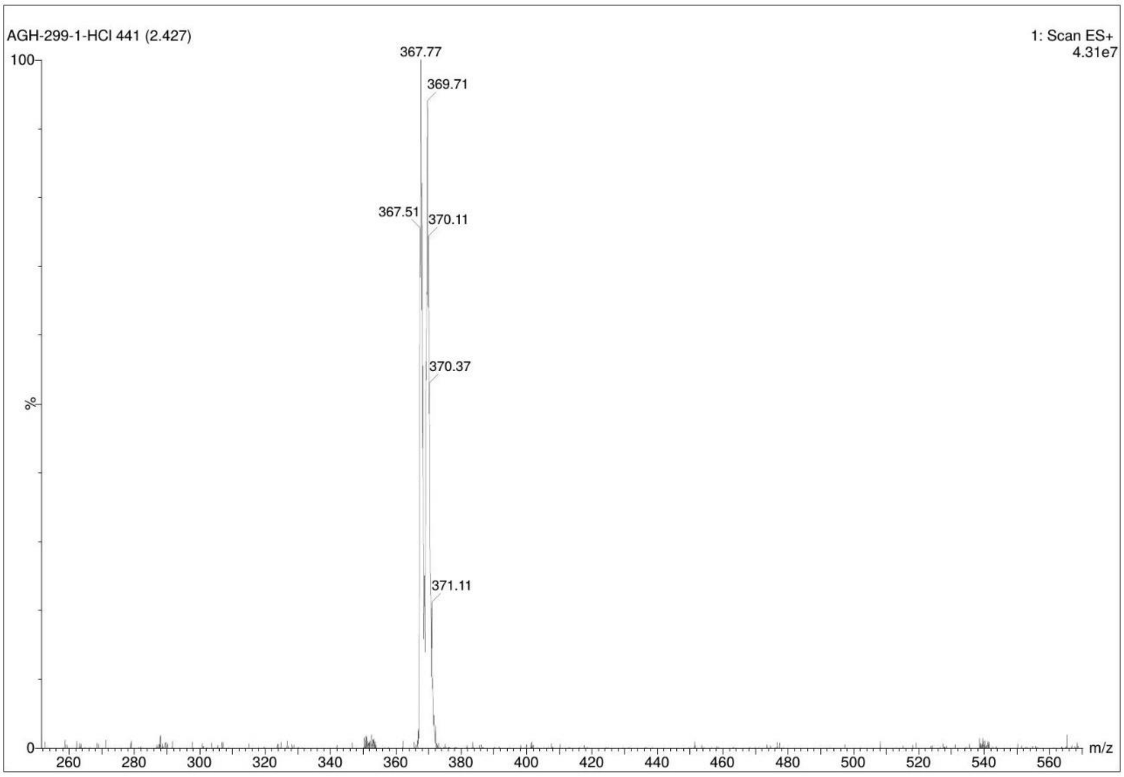

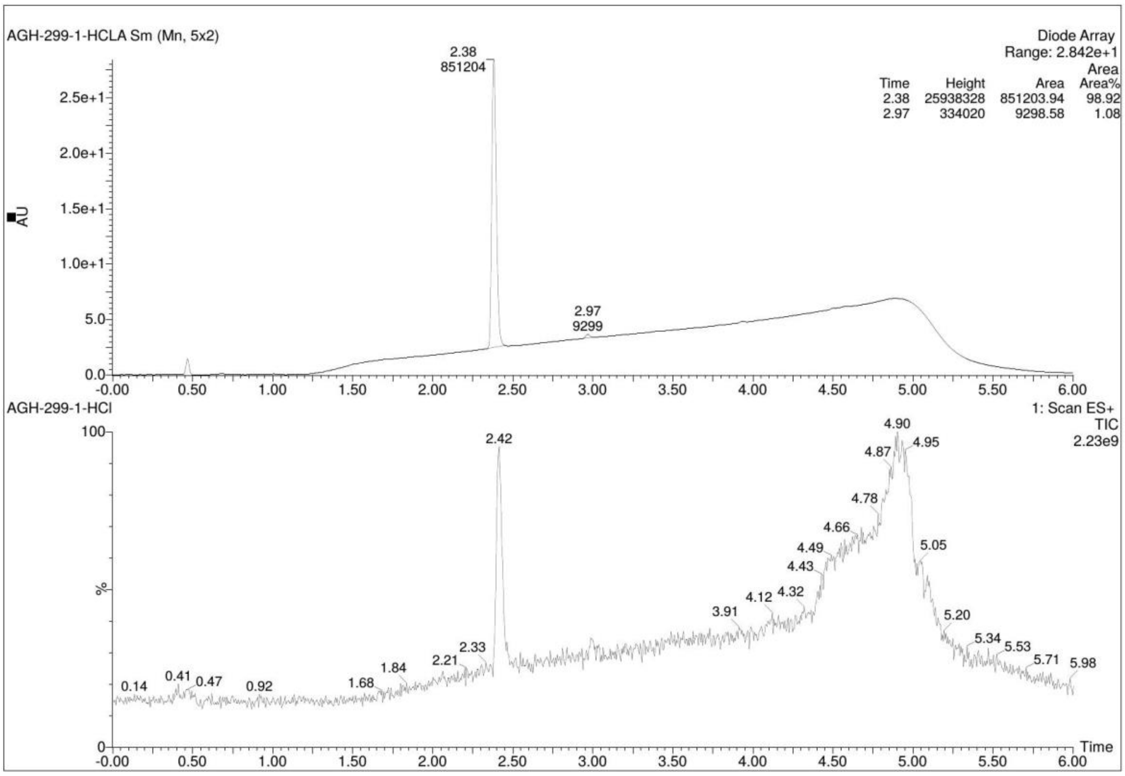
AGH-299-1-HCLA Sm (Mn, 5x2)
Diode Array
Range: 2.842e+1
Time Height Area Area%
2.38 25938328 851203.94 98.92
2.97 334020 9298.58 1.08
2.38
851204
2.97
9299
AU
AGH-299-1-HCl
1: Scan ES+
TIC
2.23e9
Time

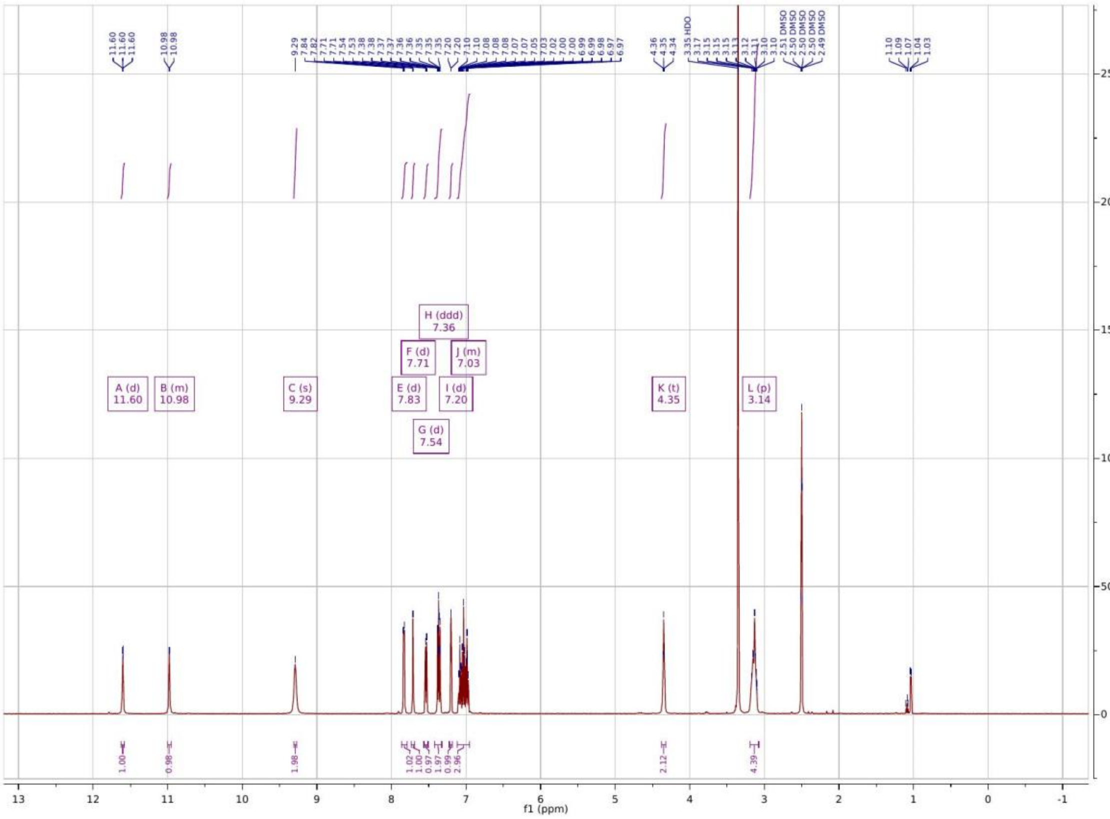
A (d)
11.60
B (m)
10.98
C (s)
9.29
E (d)
7.83
F (d)
7.71
G (d)
7.54
H (ddd)
7.36
I (d)
7.20
J (m)
7.03
K (t)
4.35
L (p)
3.14
f1 (ppm)

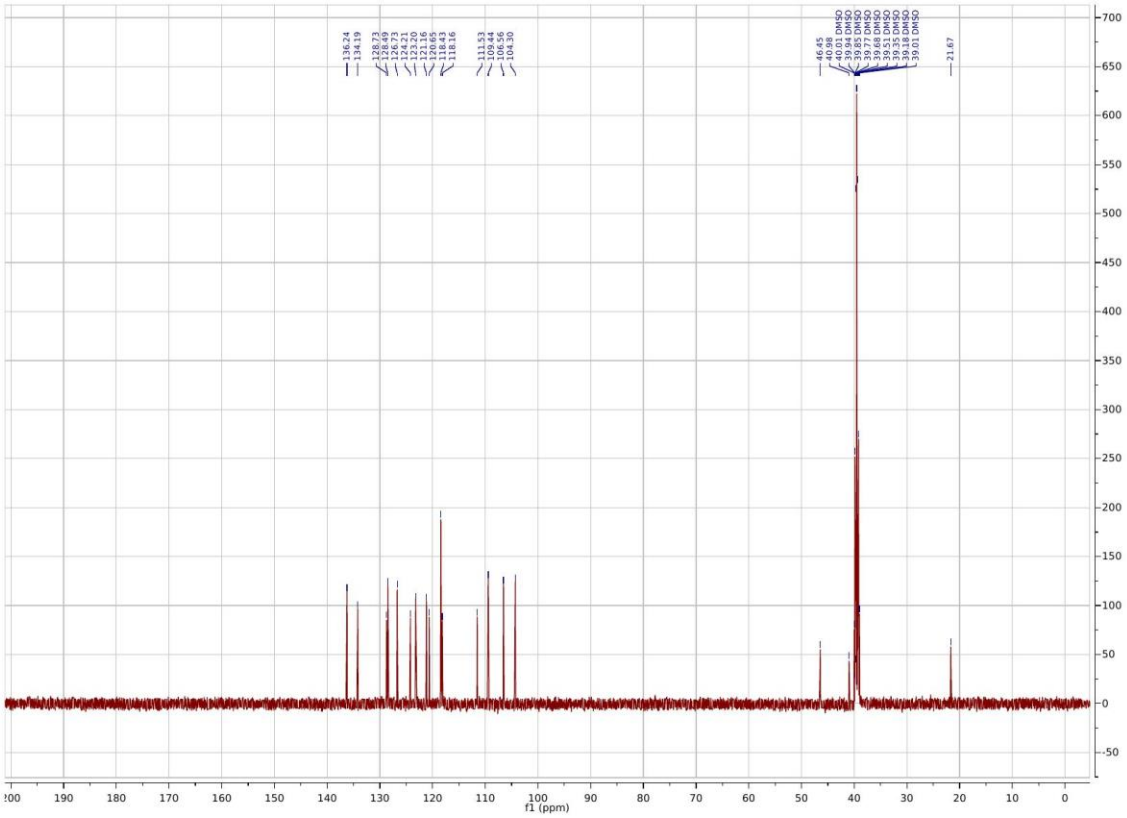

{[7-(benzyloxy)-1*H*-indol-3-yl]methyl}[2-(1*H*-indol-3-yl)ethyl]amine (19)

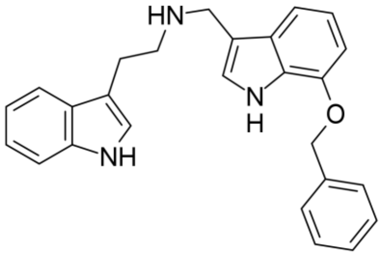

The product was obtained as a brown solid. Yield: 61%.
LCMS [M+1] = 396,09 (396,21 calculated).

^{1}H NMR (500 MHz, DMSO-d_6) δ 10.94 (s, 1H), 10.75 (s, 1H), 7.59 – 7.53 (m, 2H), 7.49 (dd, *J* = 8.0, 1.0 Hz, 1H), 7.44 – 7.37 (m, 2H), 7.36 – 7.28 (m, 2H), 7.18 – 7.08 (m, 3H), 7.04 (ddd, *J* = 8.2, 6.9, 1.2 Hz, 1H), 6.94 (ddd, *J* = 8.0, 7.0, 1.1 Hz, 1H), 6.90 (s, 0H), 6.85 (t, *J* = 7.8 Hz, 1H), 6.76 – 6.64 (m, 1H), 5.24 (s, 2H), 3.87 (s, 2H), 2.86 (s, 4H), 1.35 (s, 1H).
^{13}C NMR (126 MHz, DMSO-d_6) δ 145.00, 137.45, 136.21, 128.69, 128.35, 127.67, 127.54, 127.49, 127.27, 126.52, 123.03, 122.51, 120.78, 118.71, 118.32, 118.08, 114.30, 112.66, 111.84, 111.29, 102.86, 69.06, 49.72, 44.38, 25.39.

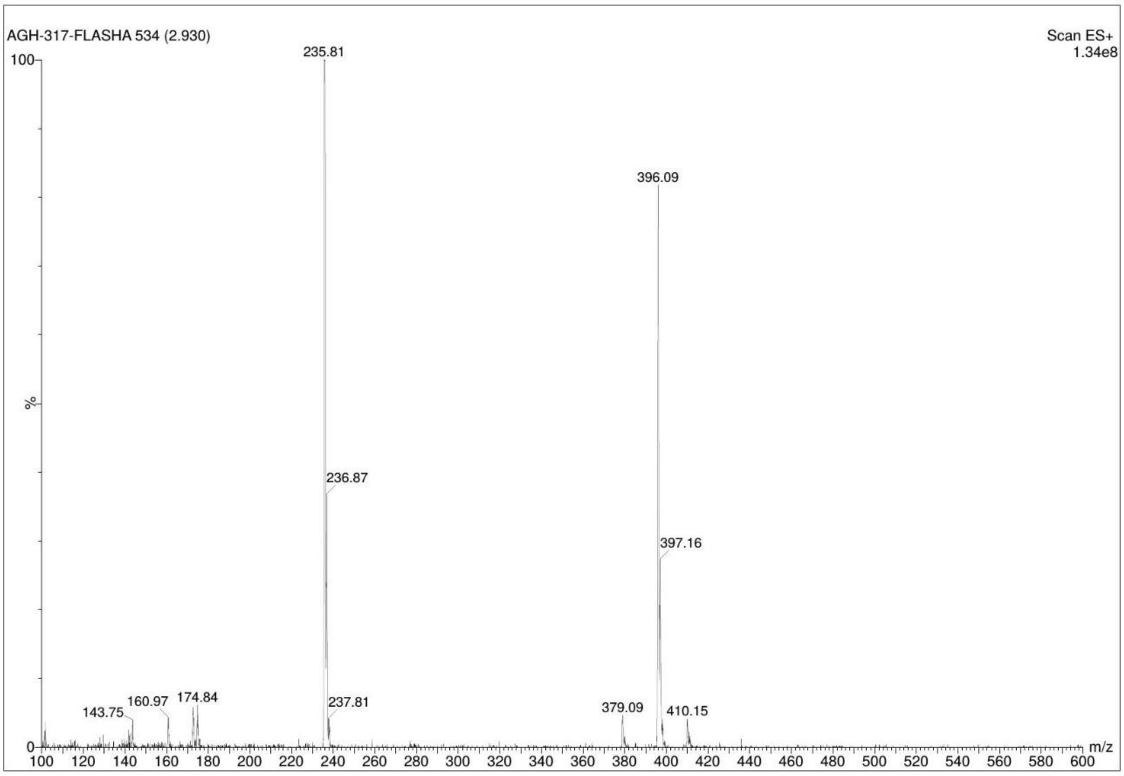

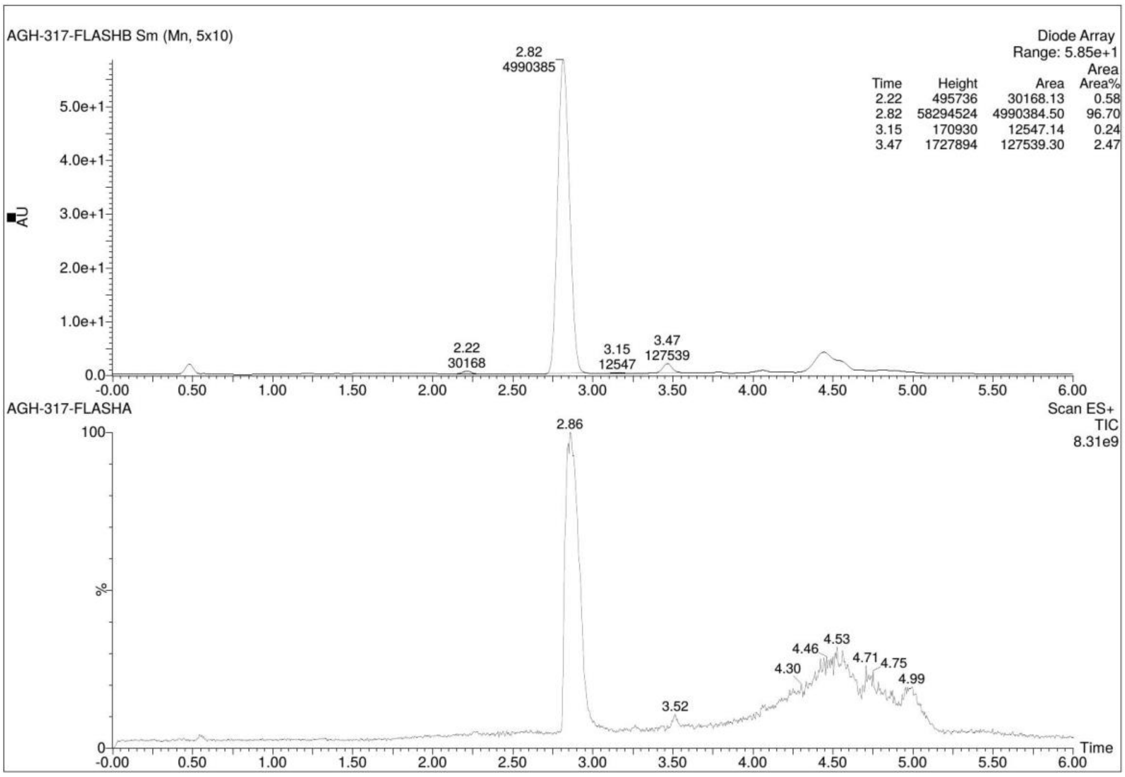

Time	Height	Area	Area%
2.22	495736	30168.13	0.58
2.82	58294524	4990384.50	96.70
3.15	170930	12547.14	0.24
3.47	1727894	127539.30	2.47

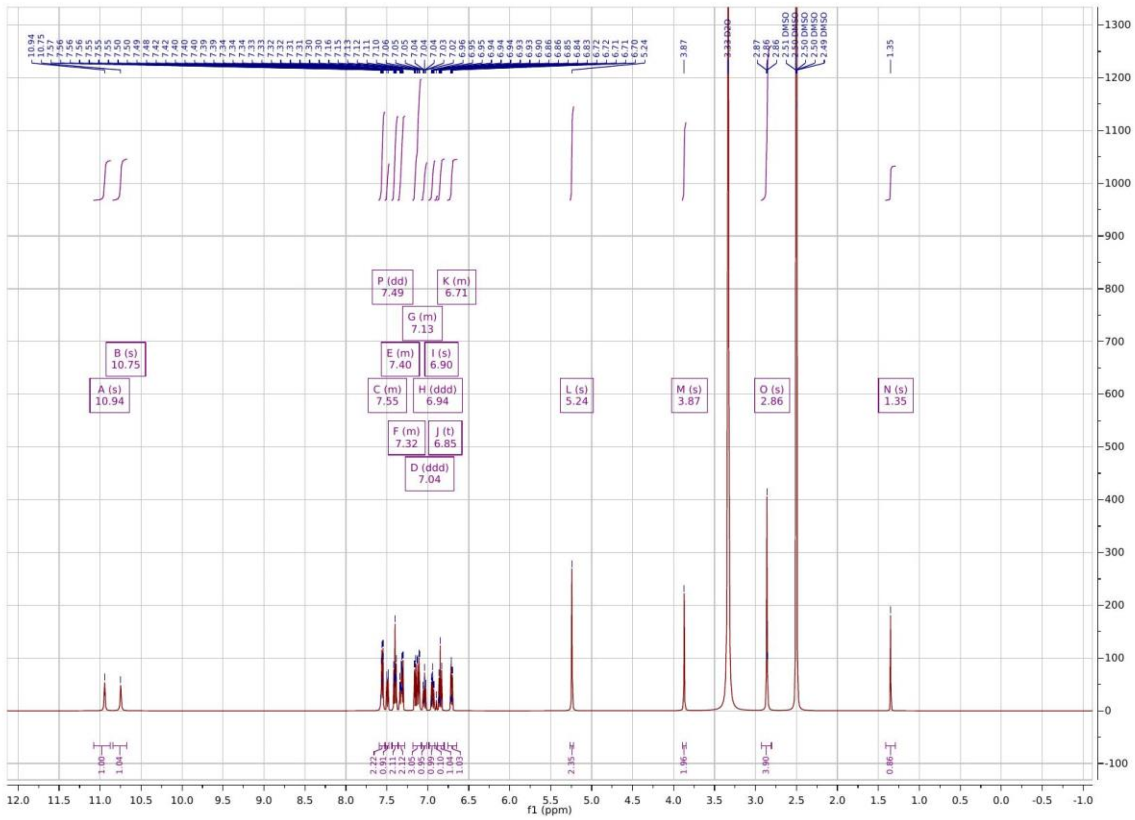

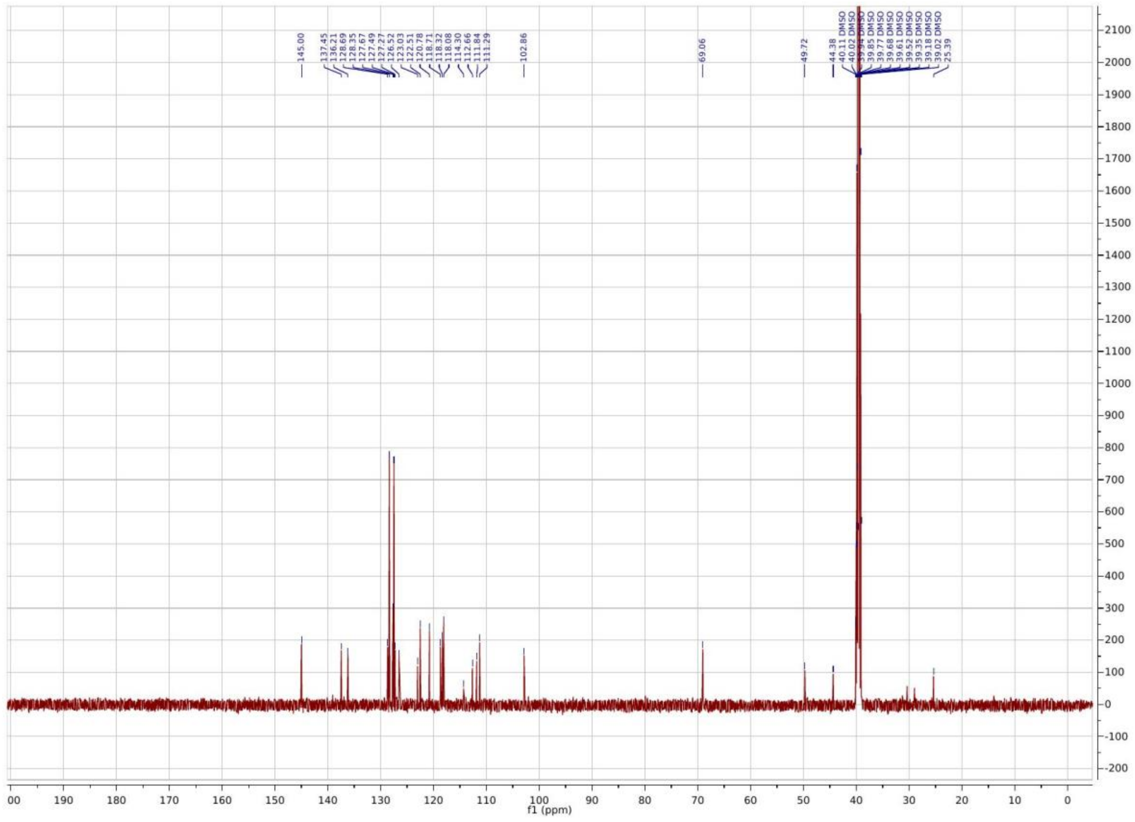

[(7-chloro-1*H*-indol-3-yl)methyl][2-(5-methoxy-1*H*-indol-3-yl)ethyl]amine (20)

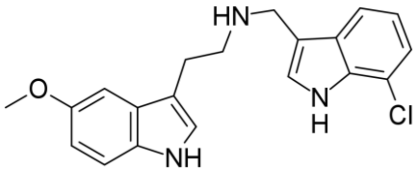

The product was obtained as a light brown solid. Yield: 37%.
LCMS [M+1] = 354,03 (354,14 calculated).

^{1}H NMR (500 MHz, DMSO-d_6) δ 11.76 (d, J = 2.6 Hz, 1H), 10.81 (d, J = 2.4 Hz, 1H), 7.78 – 7.72 (m, 1H), 7.64 (d, J = 2.5 Hz, 1H), 7.28 – 7.20 (m, 2H), 7.15 (d, J = 2.4 Hz, 1H), 7.08 (t, J = 7.8 Hz, 1H), 6.99 (d, J = 2.4 Hz, 1H), 6.72 (dd, J = 8.7, 2.4 Hz, 1H), 4.38 (s, 2H), 3.73 (s, 3H), 3.17 (dd, J = 9.8, 6.4 Hz, 2H), 3.11 – 3.00 (m, 2H).
^{13}C NMR (126 MHz, DMSO-d_6) δ 164.85, 153.12, 132.74, 131.37, 128.76, 128.50, 127.04, 123.87, 121.16, 120.25, 117.84, 116.06, 112.22, 111.30, 109.22, 106.67, 99.94, 55.36, 46.37, 40.98, 21.76.

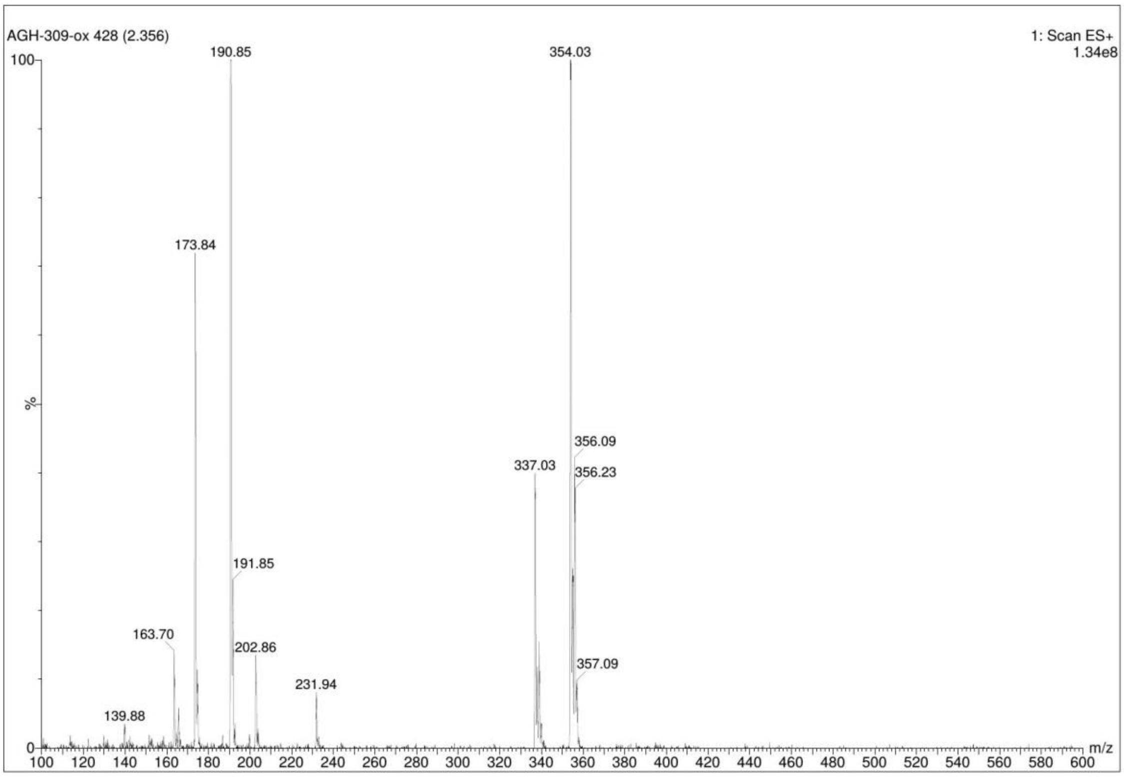

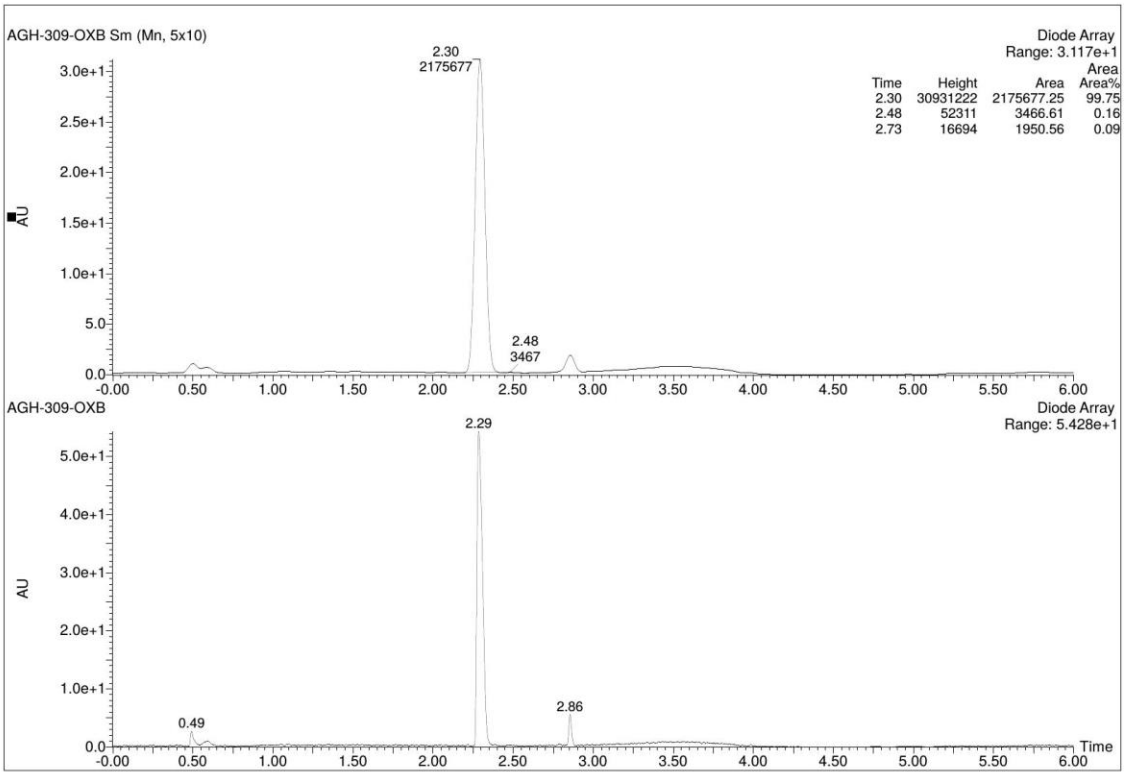
AGH-309-OXB Sm (Mn, 5x10)
Diode Array
Range: 3.117e+1
Time Height Area Area%
2.30 30931222 2175677.25 99.75
2.48 52311 3466.61 0.16
2.73 16694 1950.56 0.09
2.30
2175677
2.48
3467
AU
AGH-309-OXB
Diode Array
Range: 5.428e+1
2.29
0.49
2.86
Time

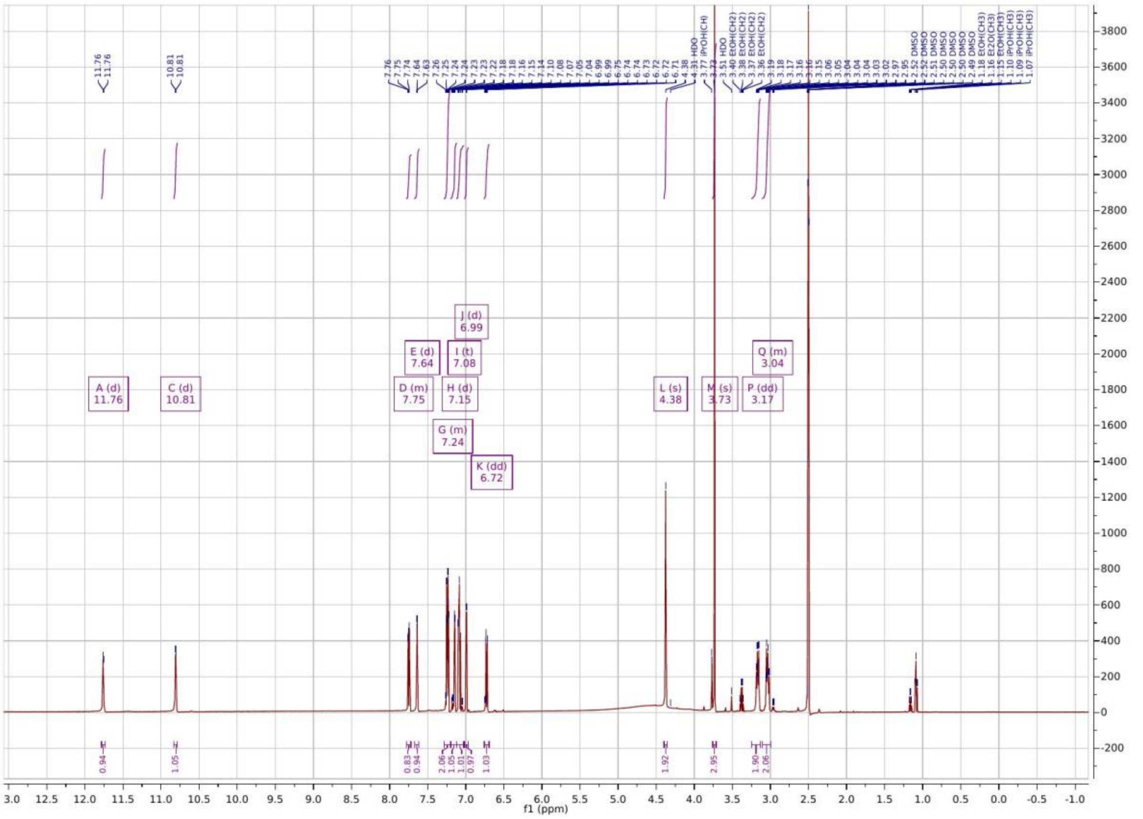
A (d)
11.76
C (d)
10.81
D (m)
7.75
E (d)
7.64
G (m)
7.24
H (d)
7.15
I (t)
7.08
J (d)
6.99
K (dd)
6.72
L (s)
4.38
M (s)
3.73
P (dd)
3.17
Q (m)
3.04
f1 (ppm)

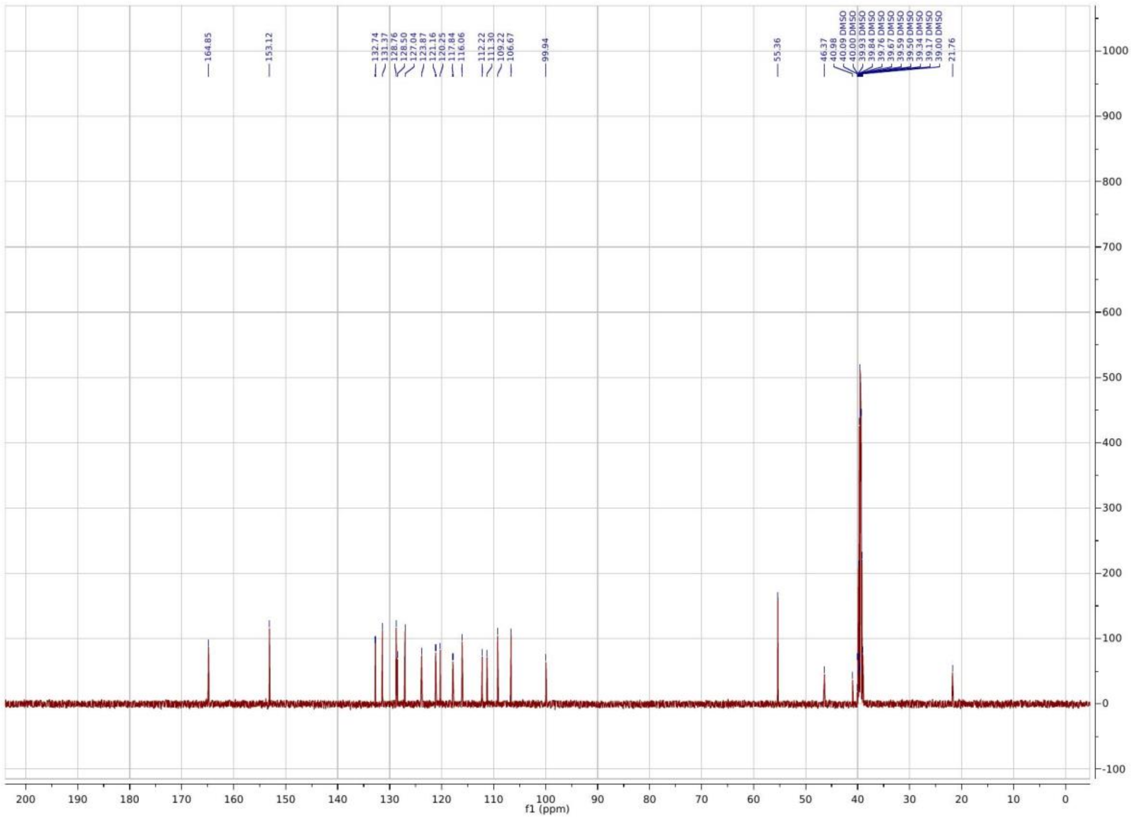

[(6-chloro-1*H*-indol-3-yl)methyl][2-(5-methoxy-1*H*-indol-3-yl)ethyl]amine (21)

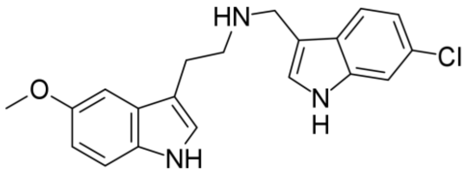

The product was obtained as a beige solid. Yield: 44%.
LCMS [M+1] = 354,03 (354,14 calculated).

^{1}H NMR (500 MHz, DMSO-d_6) δ 11.63 (d, *J* = 2.5 Hz, 1H), 10.82 (d, *J* = 2.4 Hz, 1H), 7.77 (d, *J* = 8.5 Hz, 1H), 7.59 (d, *J* = 2.5 Hz, 1H), 7.49 (d, *J* = 1.9 Hz, 1H), 7.25 (d, *J* = 8.7 Hz, 1H), 7.14 (d, *J* = 2.4 Hz, 1H), 7.08 (dd, *J* = 8.5, 1.9 Hz, 1H), 7.00 (d, *J* = 2.4 Hz, 1H), 6.73 (dd, *J* = 8.7, 2.4 Hz, 1H), 4.36 (s, 2H), 3.74 (s, 3H), 3.16 (dd, *J* = 10.1, 6.2 Hz, 2H), 3.04 (dd, *J* = 9.9, 6.4 Hz, 2H).
^{13}C NMR (126 MHz, DMSO-d_6) δ 165.00, 153.12, 136.36, 131.38, 128.41, 127.05, 126.32, 125.61, 123.85, 120.12, 119.47, 112.22, 111.39, 111.30, 109.24, 105.54, 99.94, 59.76, 55.35, 46.35, 40.91, 21.76.

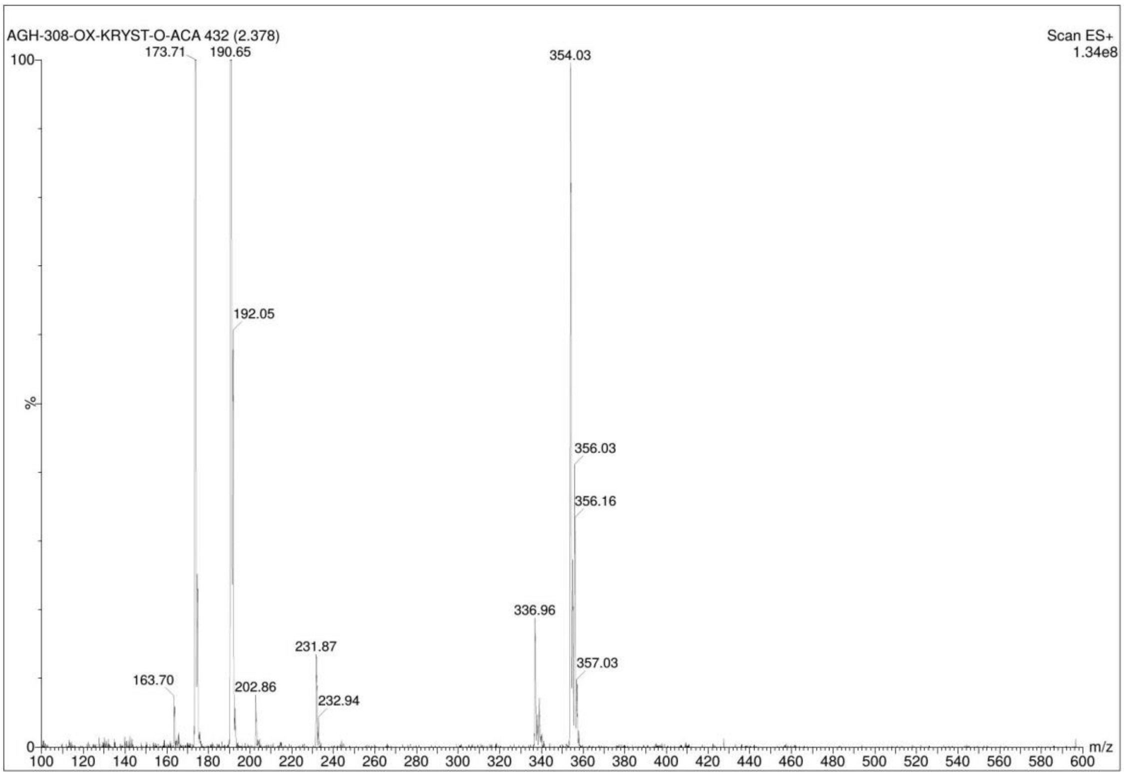

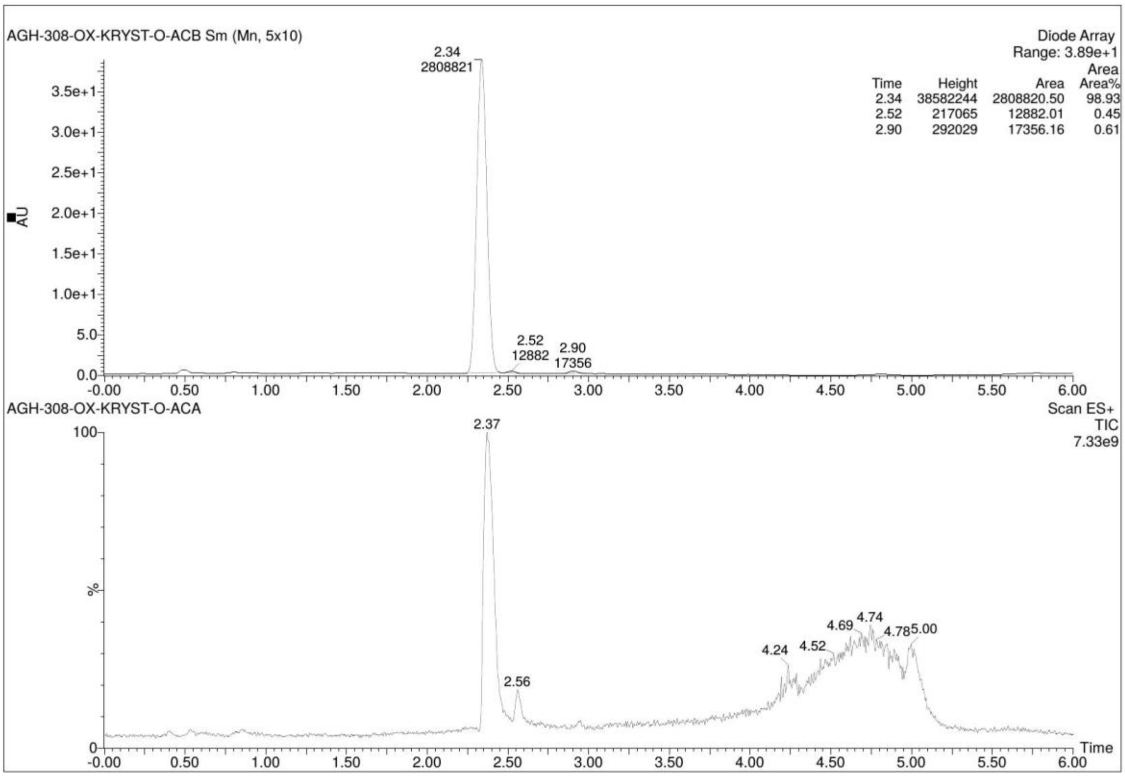

Time	Height	Area	Area Area%
2.34	38582244	2808820.50	98.93
2.52	217065	12882.01	0.45
2.90	292029	17356.16	0.61

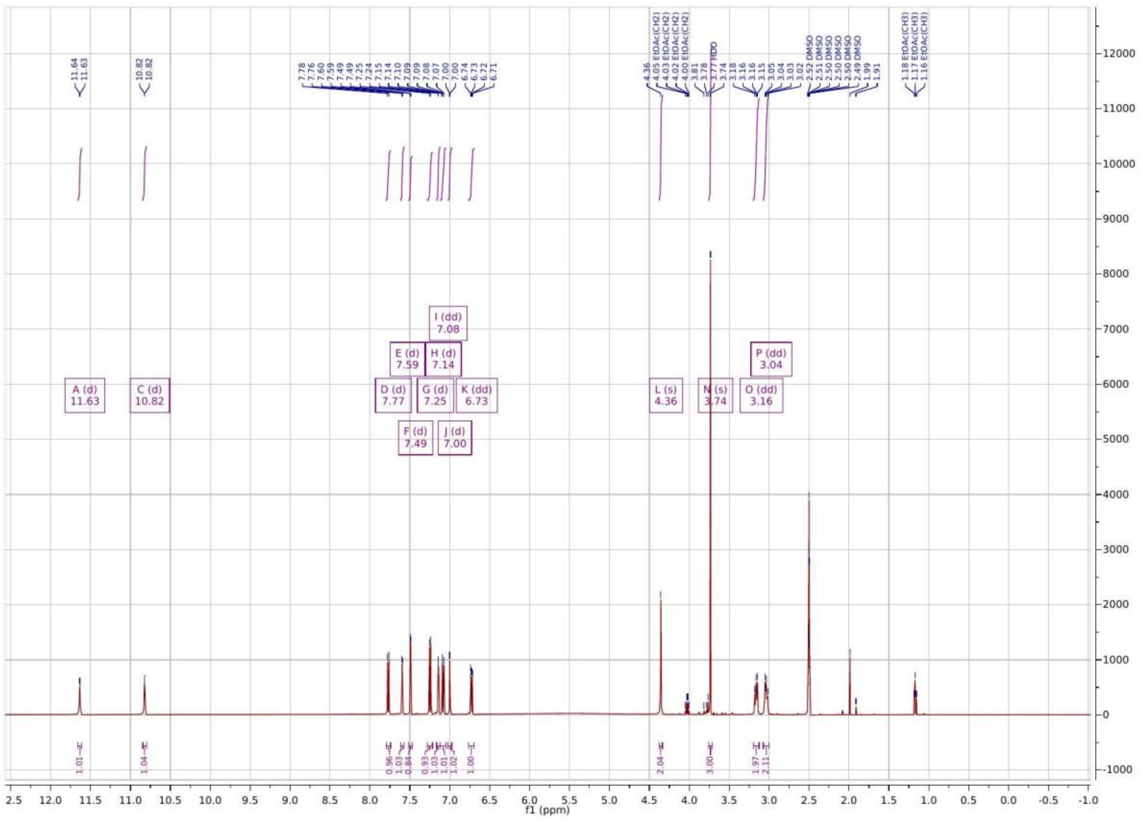

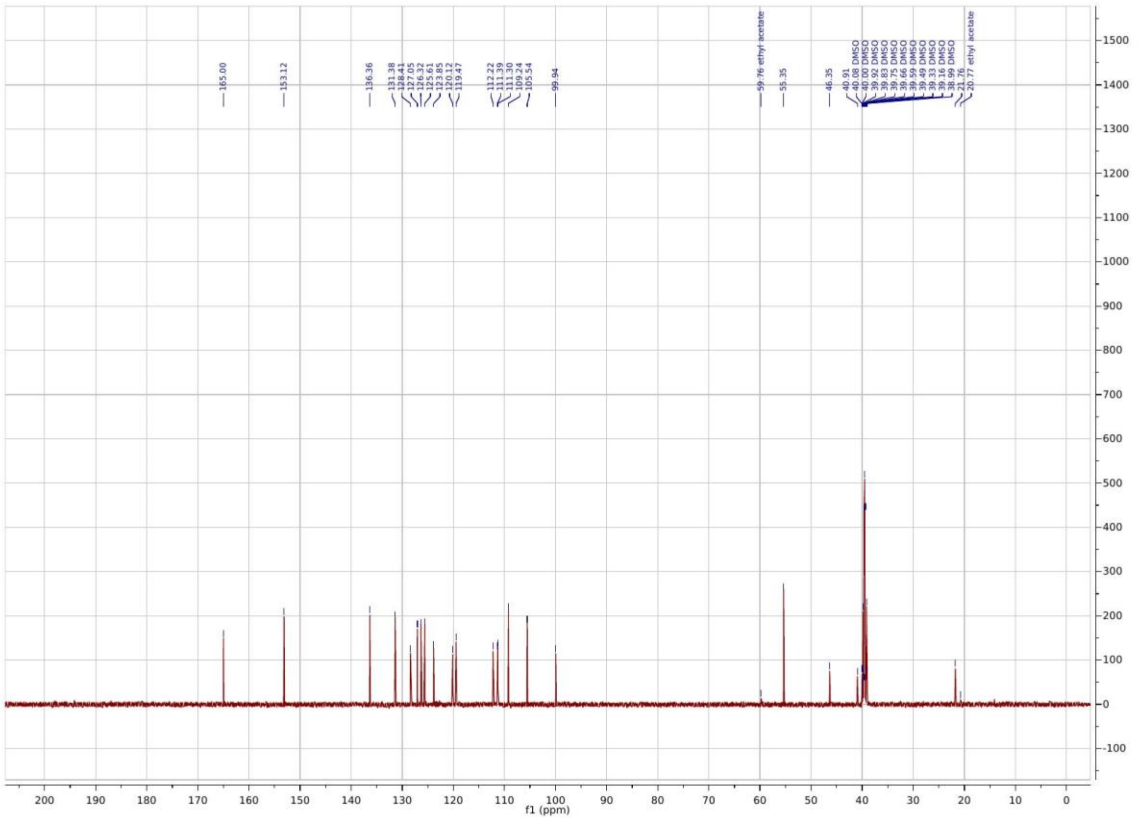

[2-(6-chloro-1*H*-indol-3-yl)ethyl][(7-chloro-1*H*-indol-3-yl)methyl]amine (22)

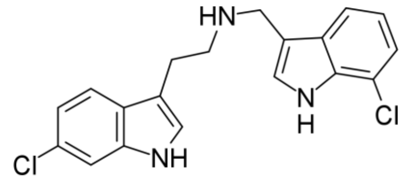

The product was obtained as a beige solid. Yield: 59%.
LCMS [M+1] = 357,78 (358,09 calculated).

^{1}H NMR (500 MHz, DMSO-d_6) δ 11.22 – 11.18 (m, 1H), 10.95 – 10.91 (m, 1H), 7.56 (dd, *J* = 4.1, 0.9 Hz, 1H), 7.57 – 7.46 (m, 2H), 7.35 (t, *J* = 2.3 Hz, 1H), 7.29 (t, *J* = 1.9 Hz, 1H), 7.16 (t, *J* = 2.4 Hz, 1H), 7.14 (dd, *J* = 7.4, 1.0 Hz, 1H), 7.00 – 6.90 (m, 3H), 3.89 (d, *J* = 0.8 Hz, 2H), 3.83 (s, 1H), 2.08 (s, 1H).

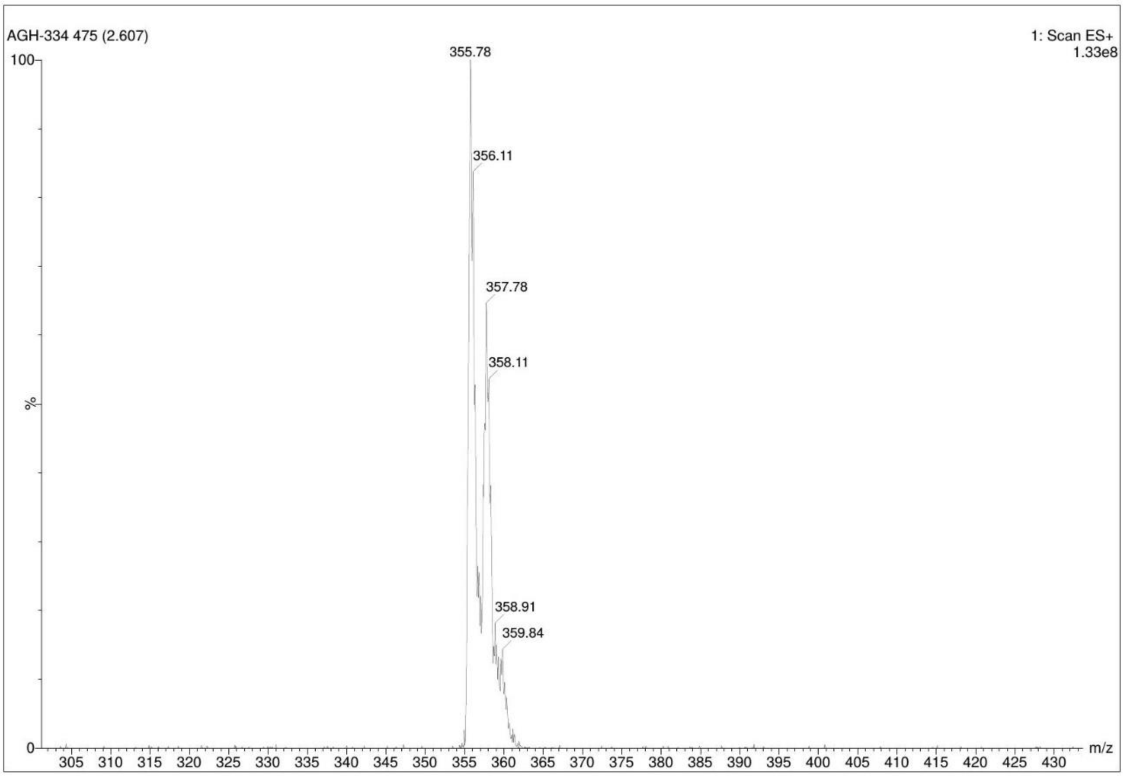

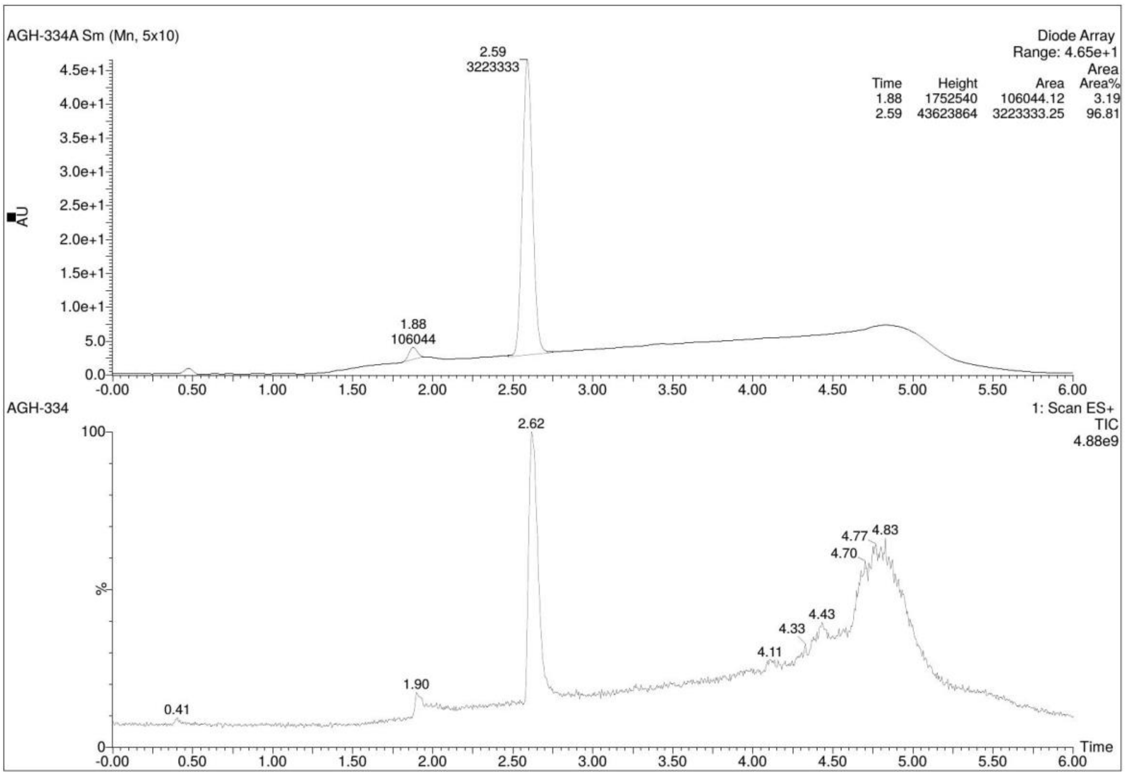
AGH-334A Sm (Mn, 5x10)
Diode Array
Range: 4.65e+1
Time	Height	Area	Area%
1.88	1752540	106044.12	3.19
2.59	43623864	3223333.25	96.81
2.59
3223333
1.88
106044
AU
AGH-334
1: Scan ES+
TIC
4.88e9
2.62
4.77
4.83
4.70
4.43
4.33
4.11
1.90
0.41
%
Time

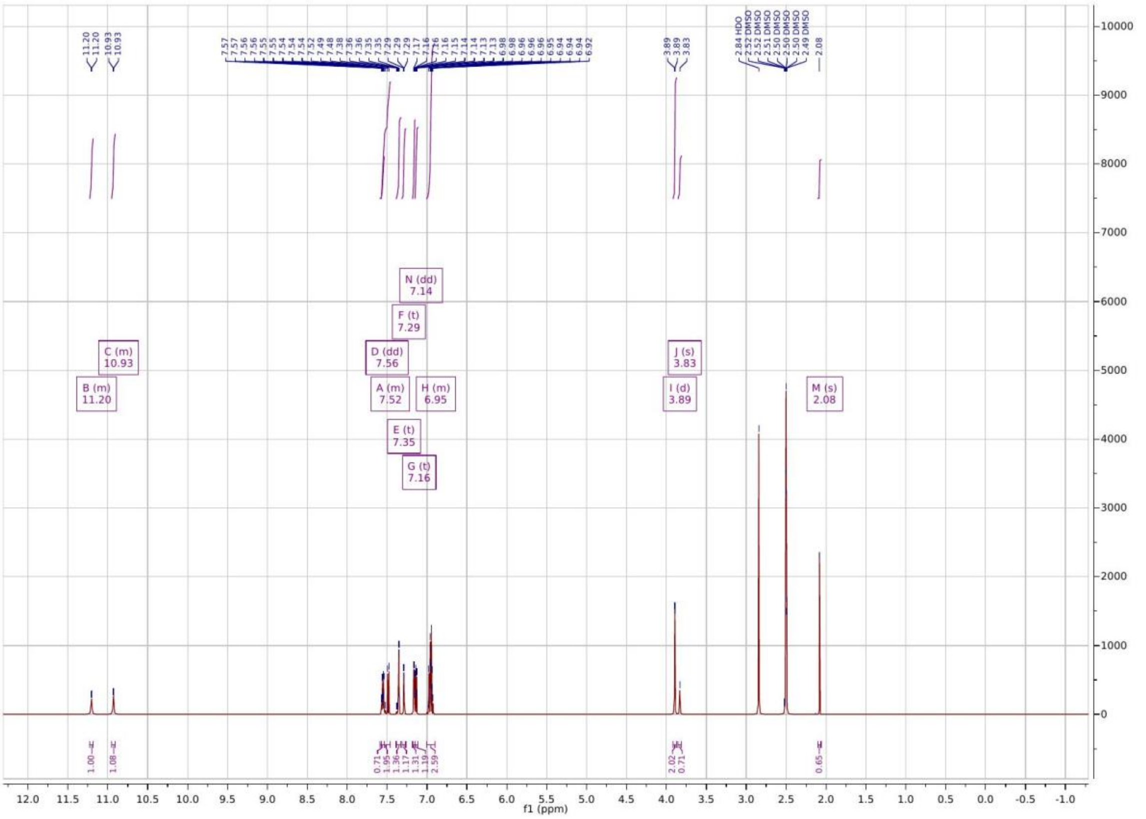
C (m)
10.93
B (m)
11.20
N (dd)
7.14
F (t)
7.29
D (dd)
7.56
A (m)
7.52
H (m)
6.95
E (t)
7.35
G (t)
7.16
J (s)
3.83
I (d)
3.89
M (s)
2.08
f1 (ppm)

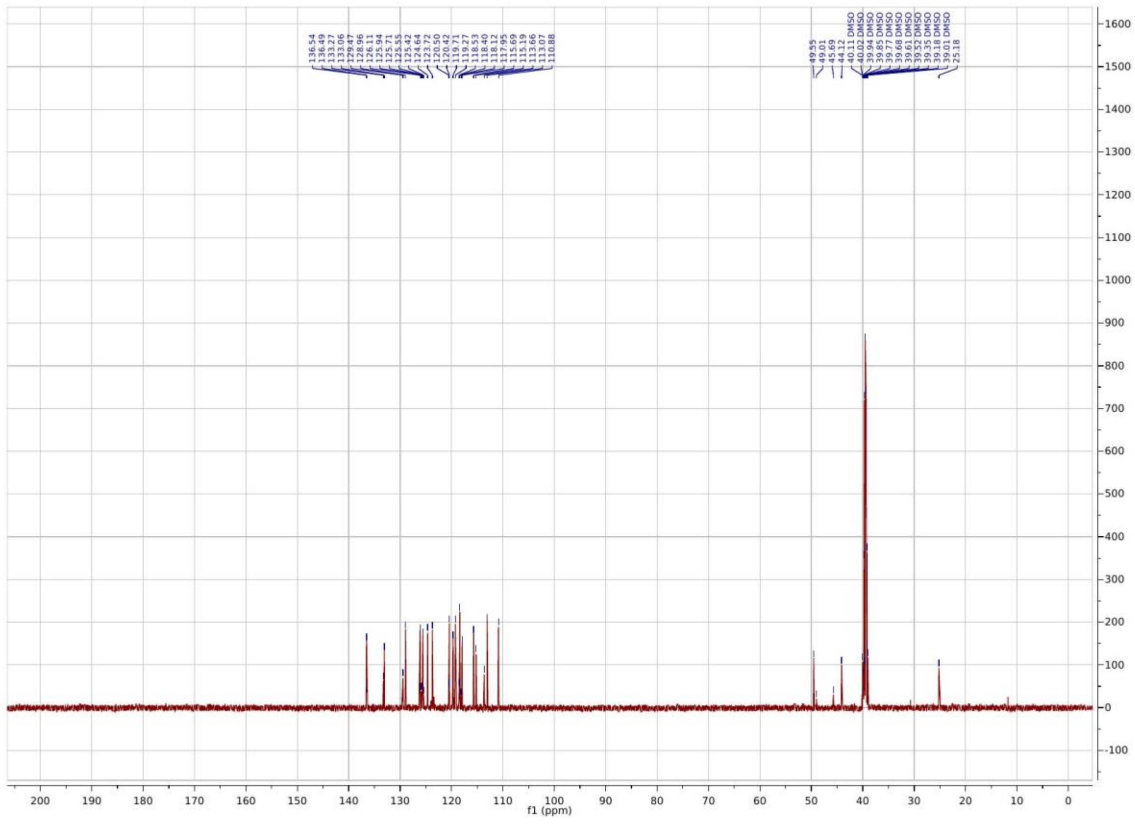

2. In vitro pharmacology

2.1 Radioligand binding assay

$5\text{-}HT_{1A}$/5-HT2A/$5\text{-}HT_6$/5-HT7/D_2 Radioligand Binding Assays. The membrane preparation and general assay procedures for the cloned receptors were adjusted to 96-microwell format as described in our former papers.58-61 The cell pellets were thawed and homogenized in 10 volumes of assay buffer using an Ultra Turrax tissue homogenizer and were centrifuged twice at 35,000 g for 15 min at 4°C, with incubation for 15 min at 37°C in between the rounds of centrifugation. The composition of the assay buffers was as follows: for 5-HT1AR: 50 mM Tris–HCl, 0.1 mM EDTA, 4 mM MgCl2, 10 μM pargyline and 0.1% ascorbate; for 5-HT2AR: 50 mM Tris–HCl, 0.1 mM EDTA, 4 mM MgCl2 and 0.1% ascorbate; for 5-HT6R: 50 mM Tris–HCl, 0.5 mM EDTA and 4 mM MgCl2, and for 5-HT7bR: 50 mM Tris–HCl, 4 mM MgCl2, 10 μM pargyline and 0.1% ascorbate; for dopamine D2LR: 50 mM Tris–HCl, 1 mM EDTA, 4 mM MgCl2, 120 mM NaCl, 5 mM KCl, 1.5 mM CaCl2 and 0.1% ascorbate.

All assays were incubated in a total volume of 200 μL in 96-well microtiter plates for 1 h at 37°C, except for 5-HT1AR and 5-HT2AR, which were incubated at room temperature and 27°C, respectively. The equilibration process was terminated by rapid filtration through Unifilter plates with a 96-well cell harvester and the radioactivity retained on the filters was quantified using a Microbeta plate reader (PerkinElmer, USA).

3 Metabolic stability

In silico AGH-292 (**14**)

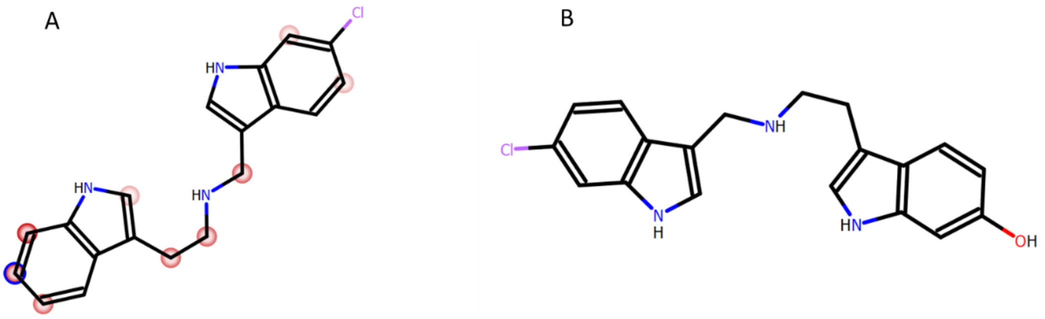

Figure S1. **A:** Prediction of the sites of metabolism by MetaSite 6.01. Blue circle marked on the functional group structures indicates the highest biotransformation probability. The fading red color shows decreasing of the metabolism probability. **B:** The most probable structure of **14** main metabolite.

In silico (**17**)

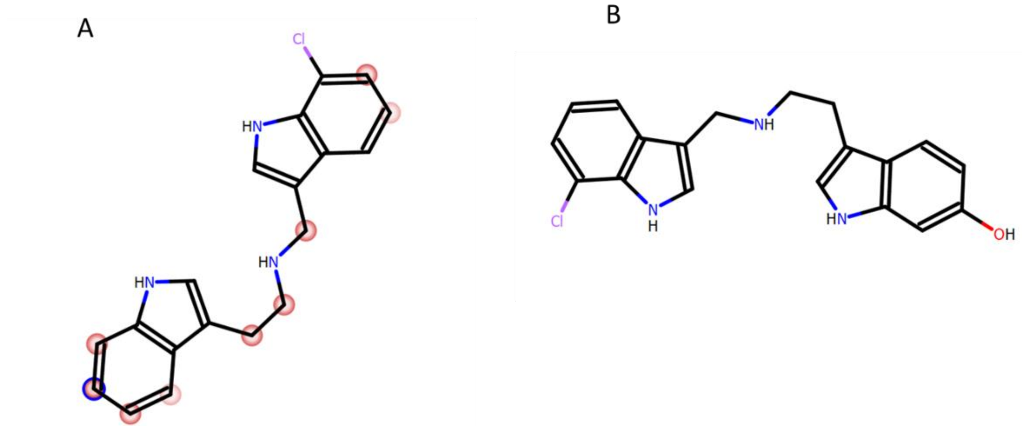

Figure S2. **A:** Prediction of the sites of metabolism by MetaSite 6.01. Blue circle marked on the functional group structures indicates the highest biotransformation probability. The fading red color shows decreasing of the metabolism probability. **B:** The most probable structure of **17** main metabolite.

In silico (**18**)

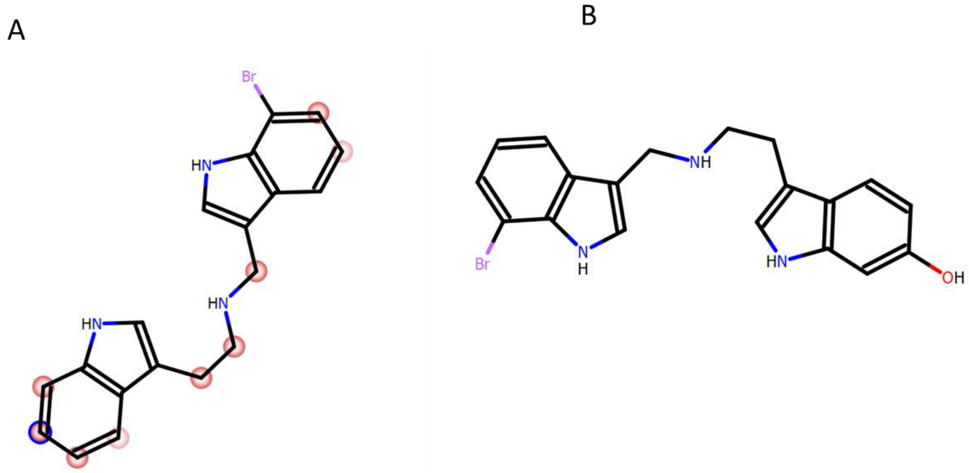

Figure S3. **A:** Prediction of the sites of metabolism by MetaSite 6.01. Blue circle marked on the functional group structures indicates the highest biotransformation probability. The fading red color shows decreasing of the metabolism probability. **B:** The most probable structure of **18** main metabolite.

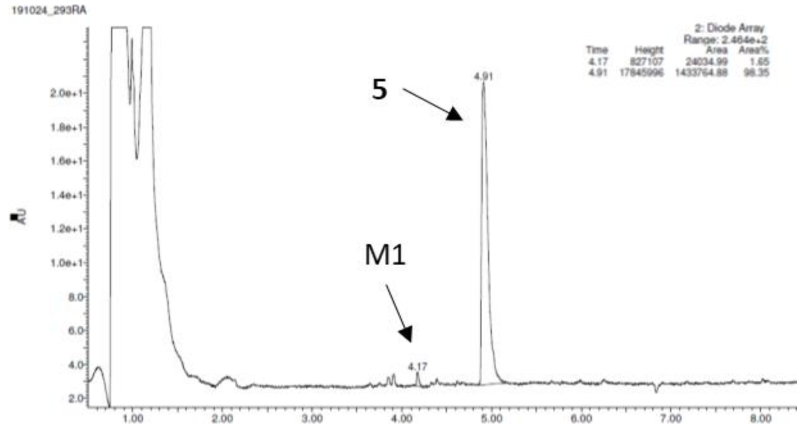

Figure S4. The UPLC spectra after 120 min reaction of **5** with mouse liver microsomes (MLMs).

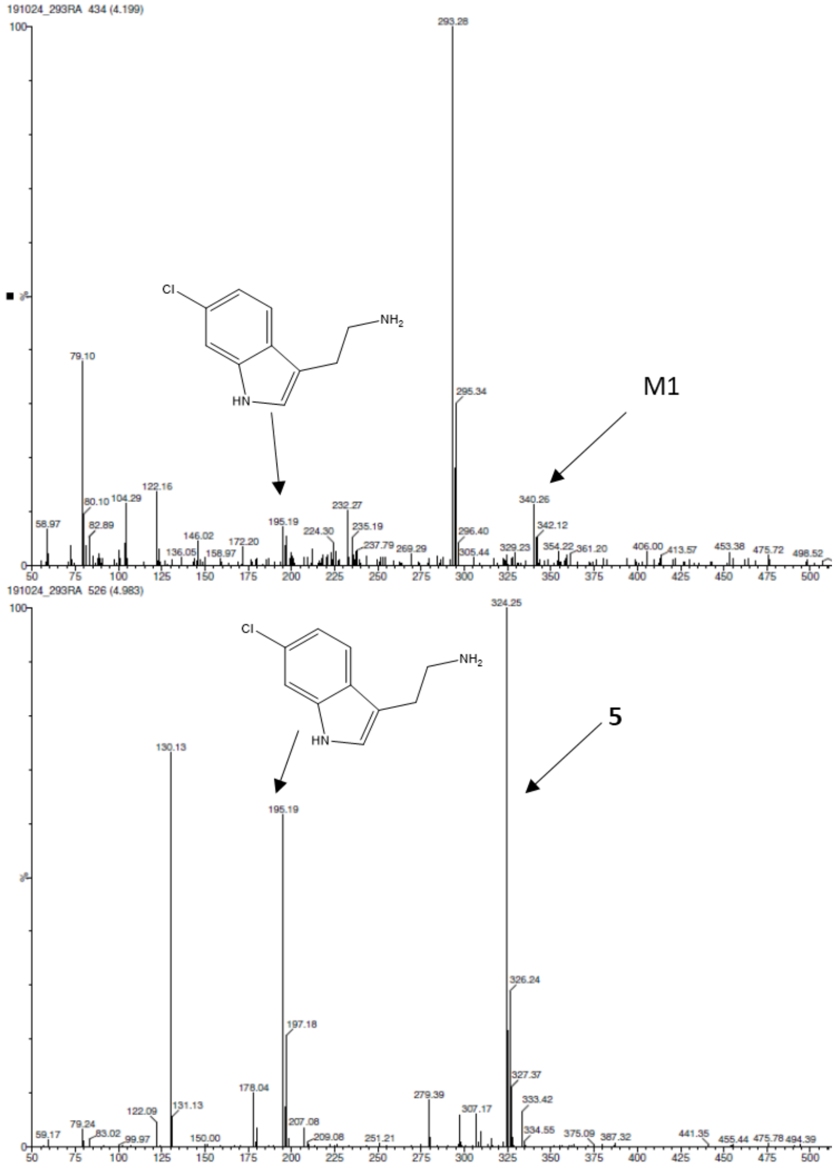

Figure S5. The MS spectra of **5** and its metabolite.

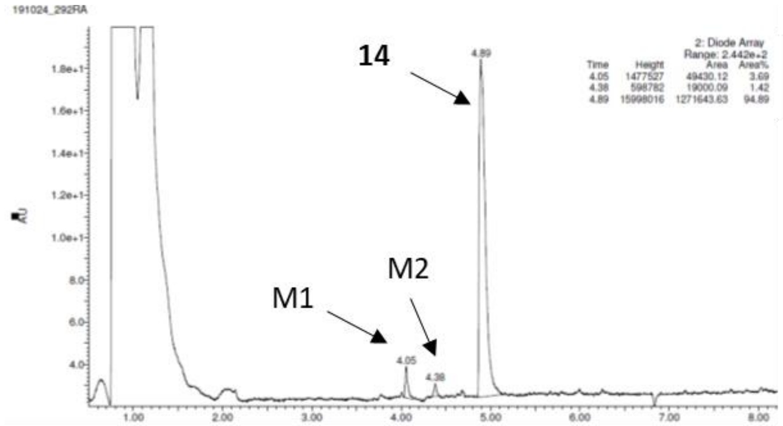

Figure S6. The UPLC spectra after 120 min reaction of **14** with mouse liver microsomes (MLMs).

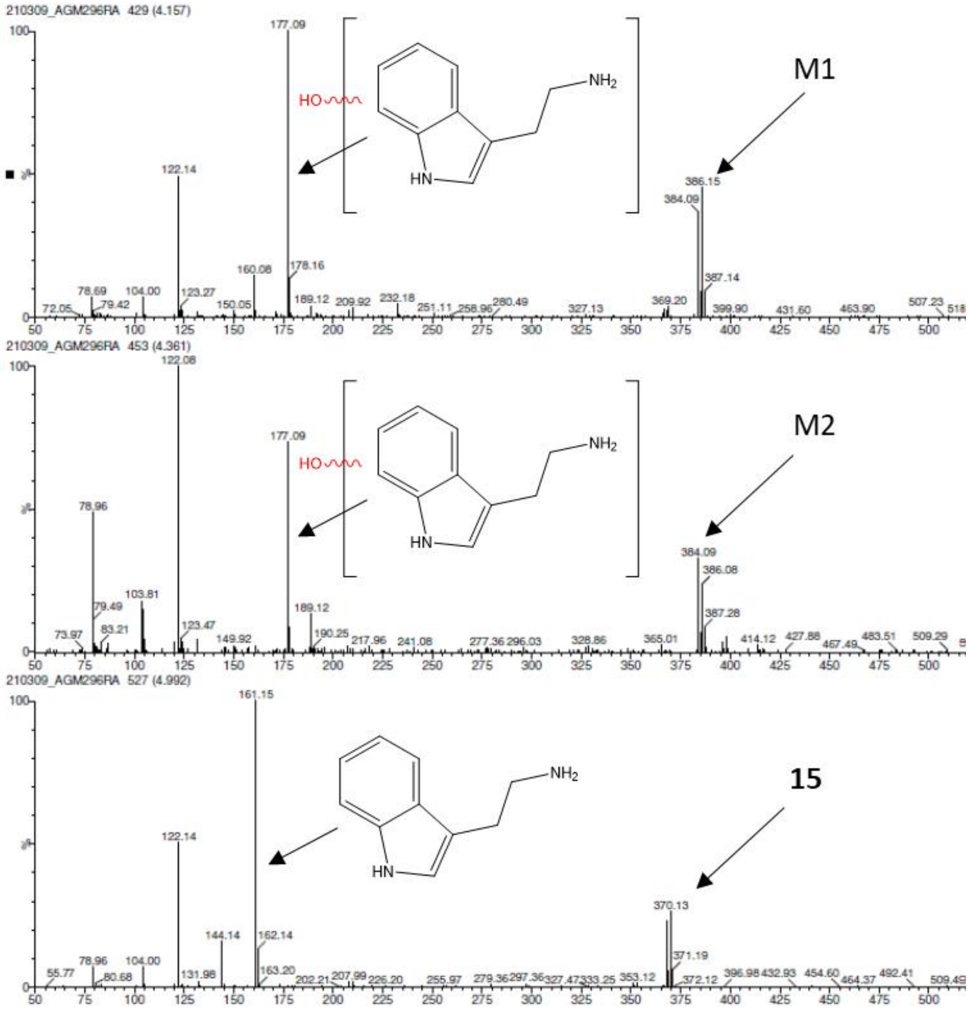

Figure S7. The MS spectra of **14** and its metabolites.

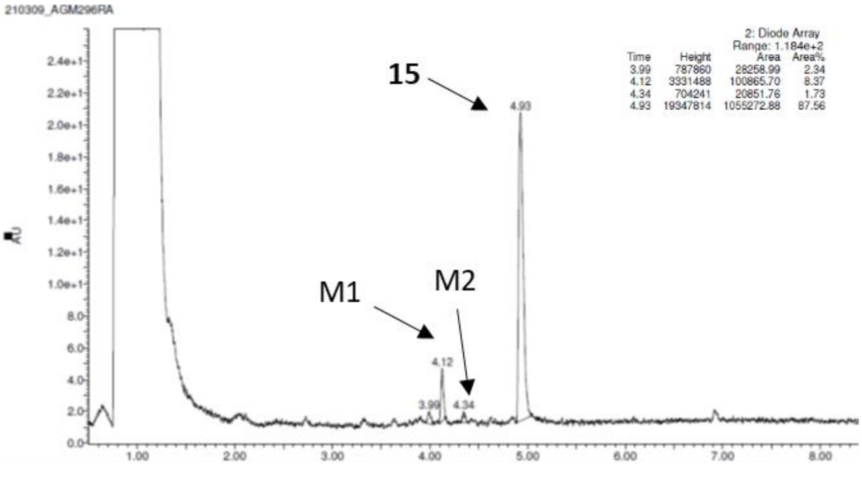

Figure S8. The UPLC spectra after 120 min reaction of **15** with mouse liver microsomes (MLMs).

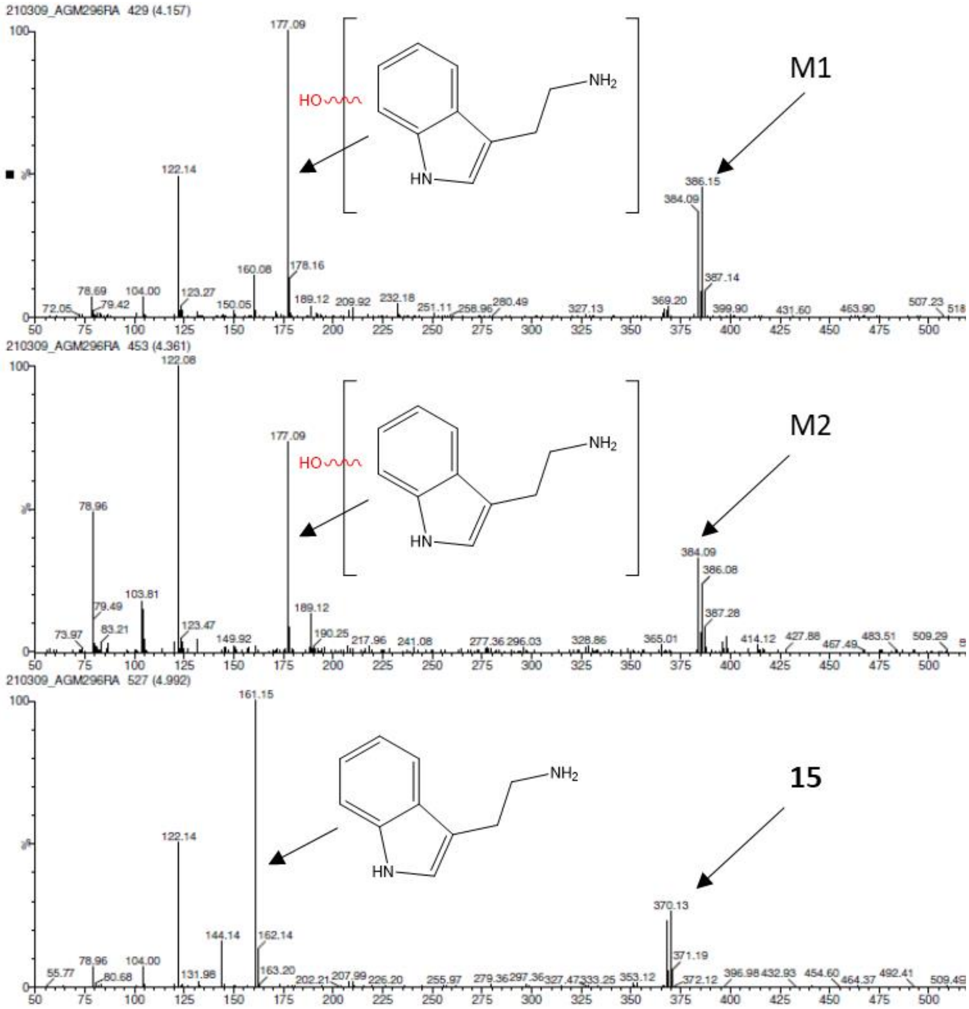

Figure S9. The MS spectra of **15** and its metabolites.

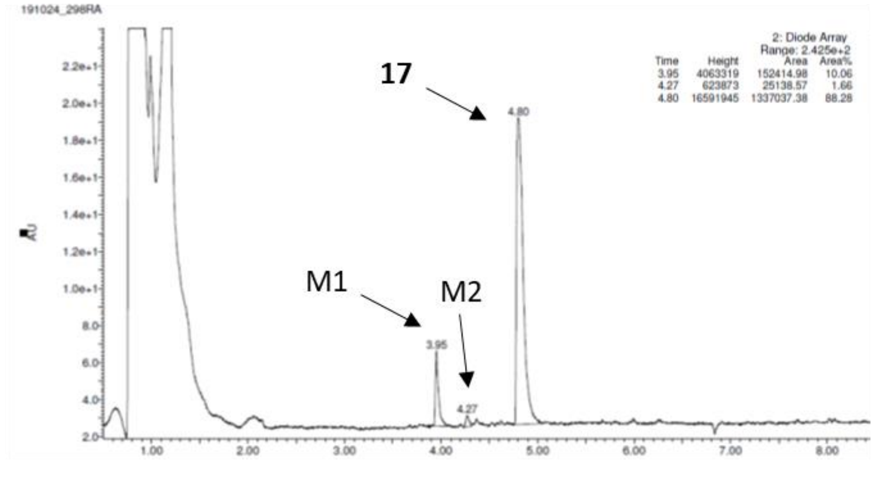

Figure S10. The UPLC spectra after 120 min reaction of **17** with mouse liver microsomes (MLMs).

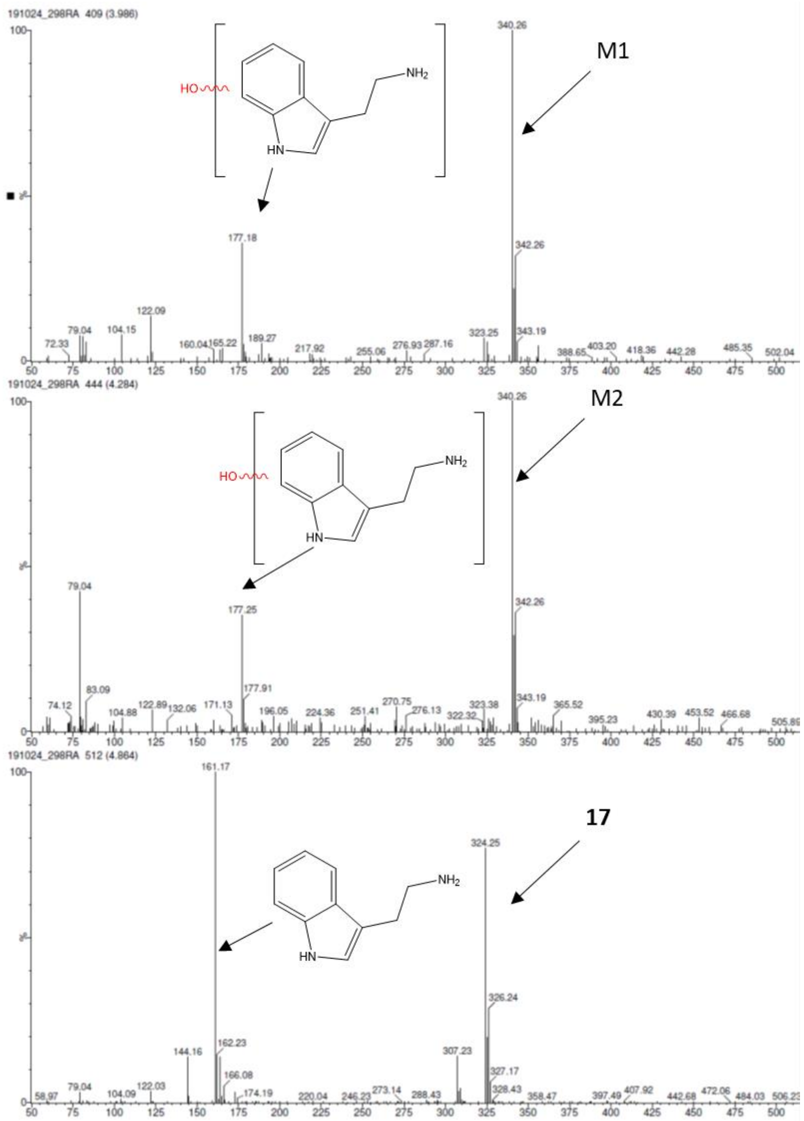

Figure S11. The MS spectra of **17** and its metabolites.

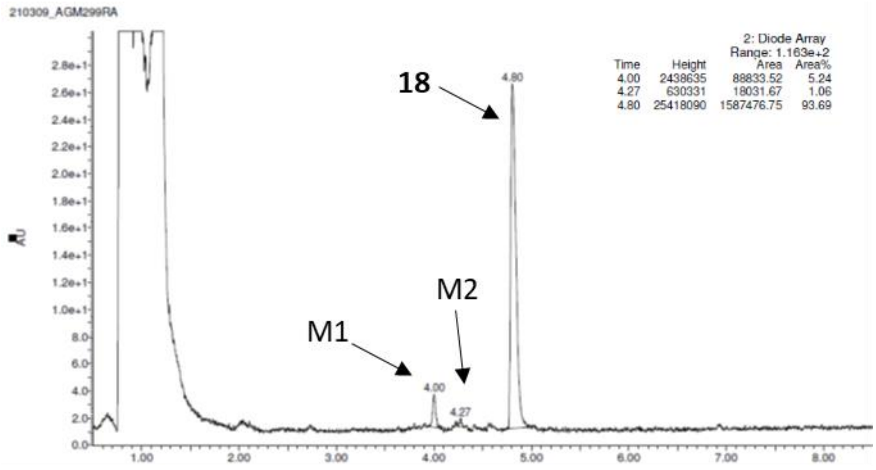

Figure S12. The UPLC spectra after 120 min reaction of **18** with mouse liver microsomes (MLMs).

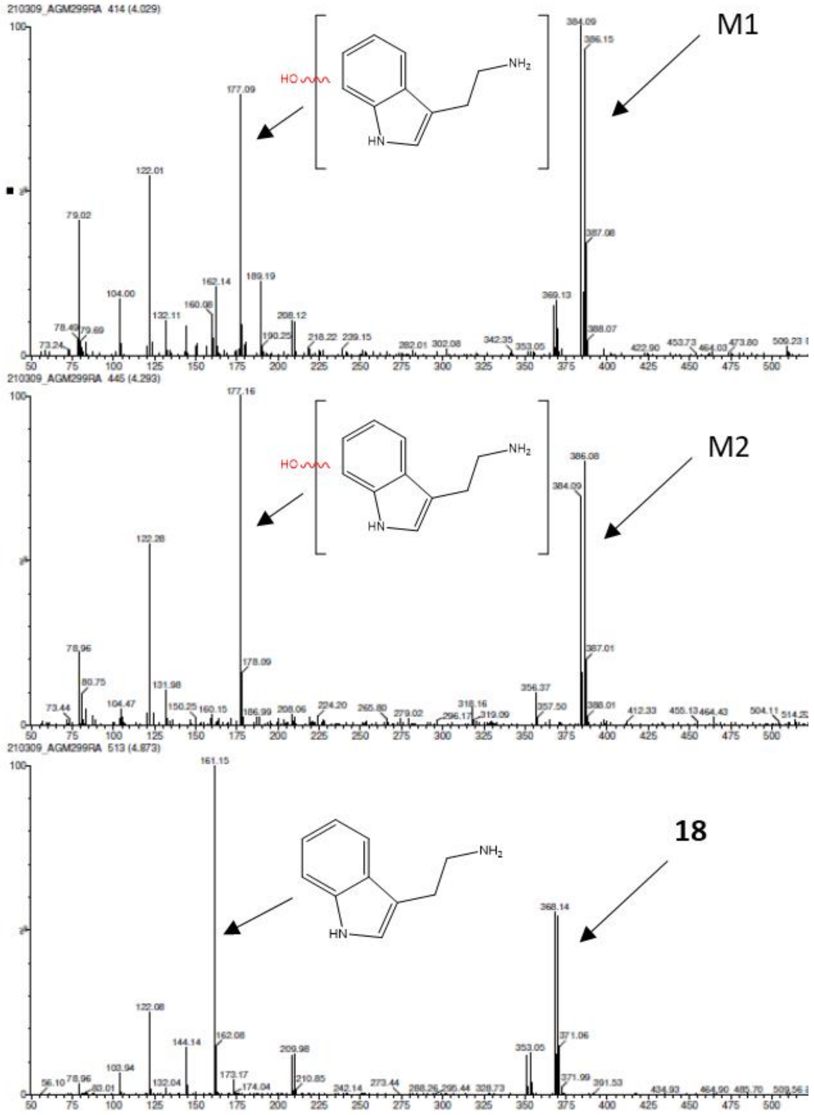

Figure S12. The MS spectra of **18** and its metabolites.
